# LTβR controls thymic portal endothelial cells for haematopoietic progenitor cell homing and T-cell regeneration

**DOI:** 10.1038/ncomms12369

**Published:** 2016-08-05

**Authors:** Yaoyao Shi, Weiwei Wu, Qian Chai, Qingqing Li, Yu Hou, Huan Xia, Boyang Ren, Hairong Xu, Xiaohuan Guo, Caiwei Jin, Mengjie Lv, Zhongnan Wang, Yang-Xin Fu, Mingzhao Zhu

**Affiliations:** 1 Key Laboratory of Infection and Immunity, Institute of Biophysics, Chinese Academy of Sciences, Beijing 100101, China; 2 University of Chinese Academy of Sciences, Beijing 100049, China; 3 Biodynamic Optical Imaging Center, College of Life Sciences, Peking University, Beijing 100871, China; 4 School of Medicine, Tsinghua University, Beijing 100084 China; 5 Department of Pathology and Immunology, UT Southwestern Medical Center, Dallas, Texas 75235-9072, USA

## Abstract

Continuous thymic homing of haematopoietic progenitor cells (HPCs) via the blood is critical for normal T-cell development. However, the nature and the differentiation programme of specialized thymic endothelial cells (ECs) controlling this process remain poorly understood. Here using conditional gene-deficient mice, we find that lymphotoxin beta receptor (LTβR) directly controls thymic ECs to guide HPC homing. Interestingly, T-cell deficiency or conditional ablation of T-cell-engaged LTβR signalling results in a defect in thymic HPC homing, suggesting the feedback regulation of thymic progenitor homing by thymic products. Furthermore, we identify and characterize a special thymic portal EC population with features that guide HPC homing. LTβR is essential for the differentiation and homeostasis of these thymic portal ECs. Finally, we show that LTβR is required for T-cell regeneration on irradiation-induced thymic injury. Together, these results uncover a cellular and molecular pathway that governs thymic EC differentiation for HPC homing.

## Abstract (web summary)

Lymphoid progenitors migrate from the bone marrow into the thymus to give rise to T and NK cell lineages. Here the authors characterize a lymphotoxin receptor beta-dependent population of thymic endothelial cells that guide lymphoid progenitor homing in the thymus.

## Graphical abstract

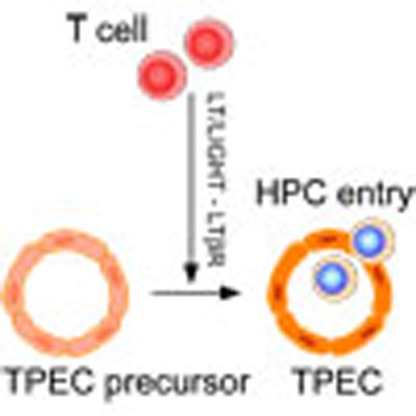

Normal thymus function depends on the continuous thymic homing of haematopoietic progenitor cells (HPCs) derived from the bone marrow. Although resident thymic progenitor cells have been reported to be able to maintain autonomous T-cell development for months when the bone marrow is deprived of progenitors^1^,^2^, a lack of competition during the self-renewal of resident thymic progenitor cells may lead to T-lineage acute lymphoblastic leukaemia^3^. However, on thymic injury, which is frequently observed during various stresses such as infection, ionizing radiation and chemotherapy, the thymic homing of HPCs appears to be a critical step for efficient thymic regeneration and T-cell recovery^4^,^5^,^6^. Given the markedly reduced thymic HPC homing efficiency on irradiation^7^, the proper manipulation of this process may have notable clinical benefits. In fact, a pilot study using pretreatment of bone marrow progenitor cells with CCL25 and CCL21 before transplantation has demonstrated increased thymic HPC homing and T-cell regeneration in mice^7^. Even so, the relatively low efficiency observed in this study demands further improvement.

Thymic endothelial cells (ECs), especially those located within the perivascular spaces (PVSs) at the corticomedullary junction area^8^,^9^,^10^,^11^,^12^, are believed to play critical roles in thymic cell homing. While a cascade of adhesion and signalling events, mainly involving P-selectin, VCAM-1 and ICAM-1, and CCL25 and CCL21/19, has been suggested to mediate the thymic homing progress^7^,^13^,^14^,^15^,^16^, their cellular basis has not been well defined. Therefore, the nature of thymic ECs, especially PVS-associated thymic portal ECs, remains largely elusive. In addition, how thymic ECs are regulated is also unknown. Further understanding of the cellular and molecular mechanisms controlling thymic ECs may provide novel insight into thymic HPC homing, and T-cell development and regeneration.

The lymphotoxin beta receptor (LTβR) signalling pathway, engaged by the ligands of lymphotoxin (LT) and/or LIGHT, plays a crucial role in the development and function of high ECs (HECs) for the lymph node (LN) homing of lymphocytes^17^,^18^,^19^,^20^,^21^. On the cellular level, strategically located dendritic cells (DCs), but likely not T or B cells, provide LT signalling to control the differentiation and function of HECs^22^. Whether and how the LTβR signalling axis coordinates the basic thymic homing process remain intriguing questions. In this study, we uncovered an interesting cellular and molecular pathway whereby positively selected T cells, but not other cells, orchestrate thymic HPC homing in an LTβR-dependent manner via thymic ECs.

## Results

### Endothelial LTβR controls thymic homing of progenitors

**Figure 1:**
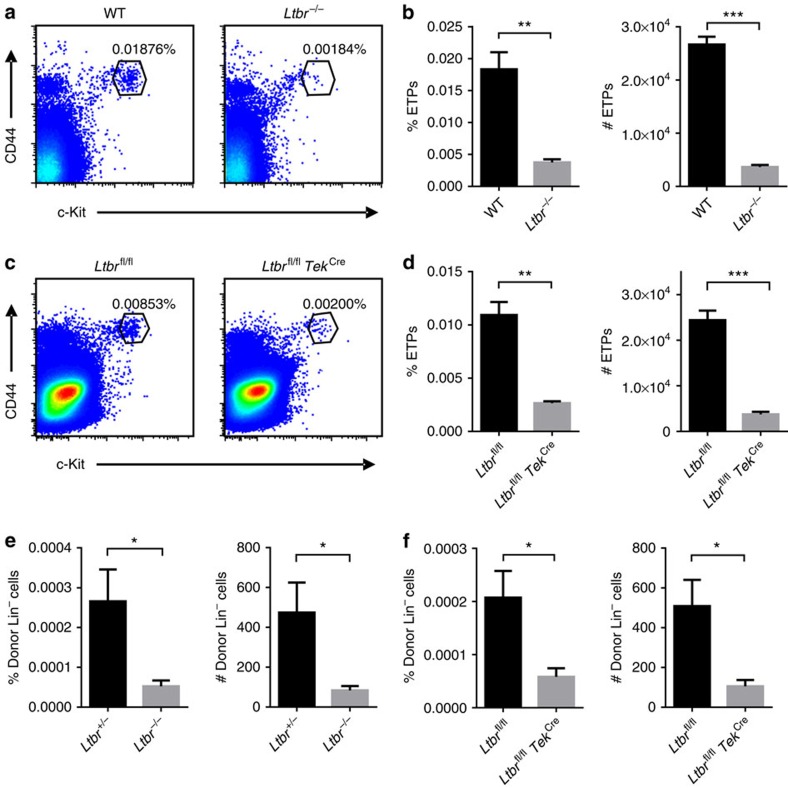
Endothelial LTβR is required for thymic progenitor cell homing. (**a**,**b**) Flow cytometric analysis of ETPs (Lin^−^CD25^−^CD44^+^c-Kit^+^) in *Ltbr*^−/−^ and control mice. (**a**) Representative dot plots are shown, gated on Lin^−^CD25^−^ thymic cells (the same below). (**b**) The graphs display the statistical analysis of the frequency and number of ETPs among total thymocytes. (**c**,**d**) Flow cytometric analysis of ETPs (Lin^−^CD25^−^CD44^+^c-Kit^+^) in *Ltbr*^fl/fl^ *Tek*^Cre^ and control mice. (**c**) Representative dot plots are shown. (**d**) The graphs display the statistical analysis of the frequency and number of ETPs among total thymocytes. (**e**,**f**) Short-term thymic homing assay in *Ltbr*^−/−^ (**e**), *Ltbr*^fl/fl^ *Tek*^Cre^ (**f**) and littermate control mice. The frequency (**e**) and number (**f**) of donor-derived lineage-negative cells among total thymocytes were analysed by flow cytometry. The data are representative of at least two independent experiments with three or more mice per group in each experiment. Error bars represent s.e.m. Asterisks mark statistically significant difference (*P<0.05, **P<0.01 and ***P<0.001 determined by two-tailed unpaired Student's *t*-test).

Thymic homing HPCs differentiate into early T-cell progenitors (ETPs), which then undergo T-cell development and maturation. Previous studies suggest that impaired thymic progenitor cell homing leads to a reduced ETP population^13^,^14^,^16^. To study whether LTβR is required for thymic progenitor cell homing, we first examined the ETP population in the thymi of *Ltbr*^−/−^ mice. Remarkably, a marked reduction in the ETP population was found in *Ltbr*^−/−^ mice compared with the wild-type (WT) control mice (Fig. 1a,b; Supplementary Fig. 1a). The data from bone marrow chimeric mice suggested that LTβR expression on radioresistant cells, but not haematopoietic cells, is required for a normal thymic ETP population (Supplementary Fig. 1b,c).

LTβR is well known to be involved in HEC differentiation and function. LTβR is also expressed on thymic ECs (Supplementary Fig. 1d). To determine whether LTβR may directly regulate ECs during thymic ETP population, we generated *Ltbr*^fl/fl^ *Tek*^Cre^ mice, in which LTβR is specifically deleted on ECs and haematopoietic cells. A marked reduction in the ETP population was observed in *Ltbr*^fl/fl^ *Tek*^Cre^ mice (Fig. 1c,d), recapitulating the phenotype of globally LTβR-deficient mice. Because LTβR deficiency in haematopoietic cells did not influence thymic ETP population, these data indicate that LTβR may control thymic ETP population via ECs.

To directly test whether LTβR is required for thymic progenitor cell homing, a short-term thymic homing assay was performed as previously described^7^,^16^,^23^. Two days after bone marrow transfer, the donor-derived thymic settling progenitor cells were examined by flow cytometry. A small but consistent number of donor-derived progenitor cells could be detected in the thymi of WT or heterozygous hosts (Supplementary Fig. 2a,b) at a level comparable to that reported previously^16^,^23^. Strikingly, the percentage and number of the donor-derived thymic settling progenitor cells were significantly reduced in *Ltbr*^−/−^ mice (Fig. 1e). A similar defect was confirmed in *Ltbr*^fl/fl^ *Tek*^Cre^ mice (Fig. 1f). The reduced thymic homing of transferred HPCs is unlikely due to their preferential distribution elsewhere. A comparable percentage and number of donor-derived HPCs were found between *Ltbr*^fl/fl^ *Tek*^Cre^ and control animals in the blood circulation, spleen and bone marrow (Supplementary Fig. 3a–c). Thus, endothelial LTβR plays a crucial role in thymus-specific progenitor cell homing.

### LT and LIGHT control thymic homing of progenitors

**Figure 2:**
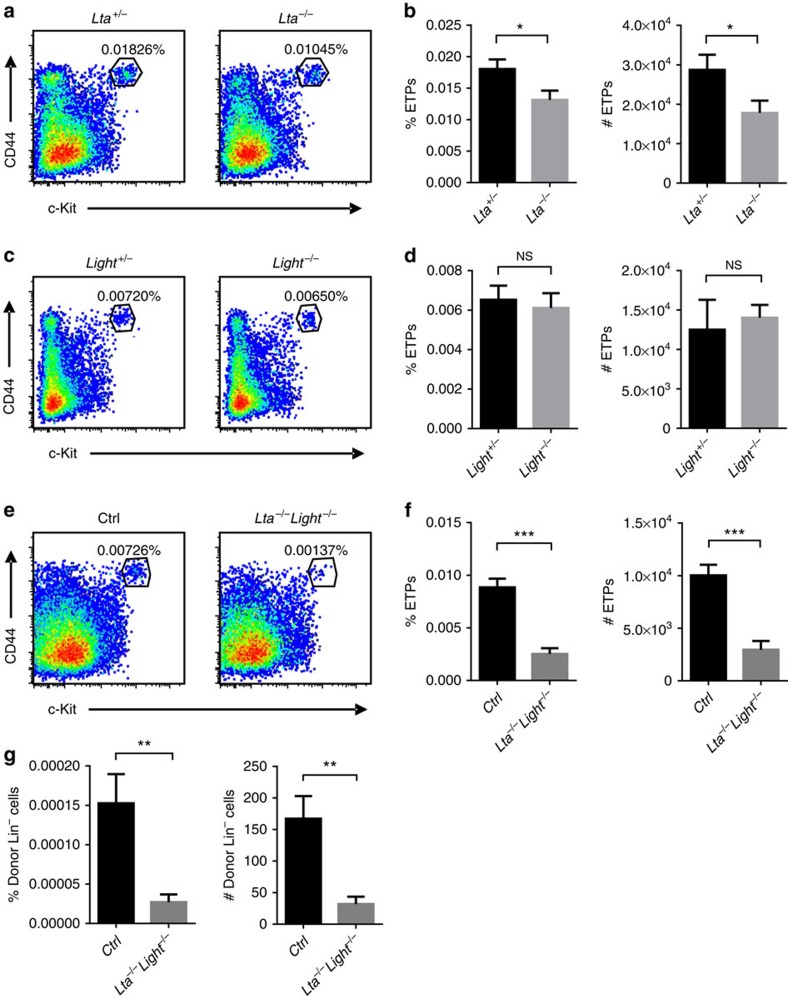
LT and LIGHT coordinate to control thymic ETP population and progenitor homing. (**a**,**b**) Flow cytometric analysis of ETPs (Lin^−^CD25^−^CD44^+^c-Kit^+^) in *Lta*^*+/*−^ and *Lta*^−*/*−^ mice. (**a**) Representative dot plots are shown. (**b**) The graphs display the statistical analysis of the frequency and number of ETPs among total thymocytes. (**c**,**d**) Flow cytometric analysis of ETPs (Lin^−^CD25^−^CD44^+^c-Kit^+^) in *Light*^*+/*−^ and *Light*^−*/*−^mice. (**c**) Representative dot plots are shown. (**d**) The graphs display the statistical analysis of the frequency and number of ETPs among total thymocytes. (**e**,**f**) Flow cytometric analysis of ETPs (Lin^−^CD25^−^CD44^+^c-Kit^+^) in *Lta*^−*/*−^ *Light*^−*/*−^ and littermate control mice. (**e**) Representative dot plots are shown. (**f**) The graphs display the statistical analysis of the frequency and number of ETPs among total thymocytes. (**g**) Short-term thymic homing assay in *Lta*^−*/*−^ *Light*^−*/*−^ and littermate control mice. The frequency and number of donor-derived lineage-negative cells among total thymocytes were analysed by flow cytometry. The data are representative of at least two independent experiments with three or more mice per group in each experiment. Error bars represent s.e.m. Asterisks mark statistically significant difference (*P<0.05, **P<0.01 and ***P<0.001 determined by two-tailed unpaired Student's *t*-test).

LTβR signalling can be activated by either LT or LIGHT. To study which ligand is required for thymic progenitor cell homing, we first evaluated the thymic ETP population in *Lta*^−/−^ and *Light*^−/−^ mice. While LT usually delivers the primary LTβR signalling in other scenarios, single LT deficiency resulted in only a minor defect in the thymic ETP population compared with the heterozygous mice (Fig. 2a,b). A dosage effect of LT was not observed because the thymic ETP population was comparable between WT and *Lta*^+/−^ mice (Supplementary Fig. 4a,b). However, LIGHT alone did not appear to be essential for maintaining a normal thymic ETP population (Fig. 2c,d). To test whether LT and LIGHT may play a coordinated role, we generated *Lta*^−/−^*Light*^−/−^ mice. Double deficiency of both LT and LIGHT in these mice recapitulated the thymic ETP defect in *Ltbr*^−/−^ mice (Fig. 2e,f). A short-term homing assay further confirmed the impaired thymic progenitor cell homing in *Lta*^−/−^*Light*^−/−^ mice (Fig. 2g) to a degree similar to that in *Ltbr*^−/−^ mice. These data suggest that while LT seems to play a relatively more important role than LIGHT, they coordinate to safeguard normal thymic progenitor cell homing and ETP population maintenance.

### T cells control thymic homing of progenitors

We next wondered which cells regulate thymic EC function for progenitor cell homing. DC-derived LT signalling has been reported to control HECs in the LNs^22^. In addition, thymic DCs were reported to be abundantly present in the PVSs^24^, the key site for thymic progenitor cell homing. CD11c-DTR bone marrow chimeric mice allow the sustained depletion of CD11c^+^ cells with continuous diphtheria toxin (DT) treatment. Thymic DCs were efficiently depleted after DT treatment (Supplementary Fig. 5a,b). However, the thymic ETP population was largely maintained in the continuous depletion of CD11c^+^ cells over 4 weeks (Supplementary Fig. 5c,d). B cells are another type of cells that have been reported to be important for LN homeostasis, especially for lymphatic ECs and follicular DCs, via LTβR signalling^25^,^26^. Bone marrow chimeric mice generated with WT or μMT mice demonstrated comparable thymic ETP populations (Supplementary Fig. 5e,f). These data suggest that DCs and B cells are not required for thymic progenitor cell homing.

**Figure 3:**
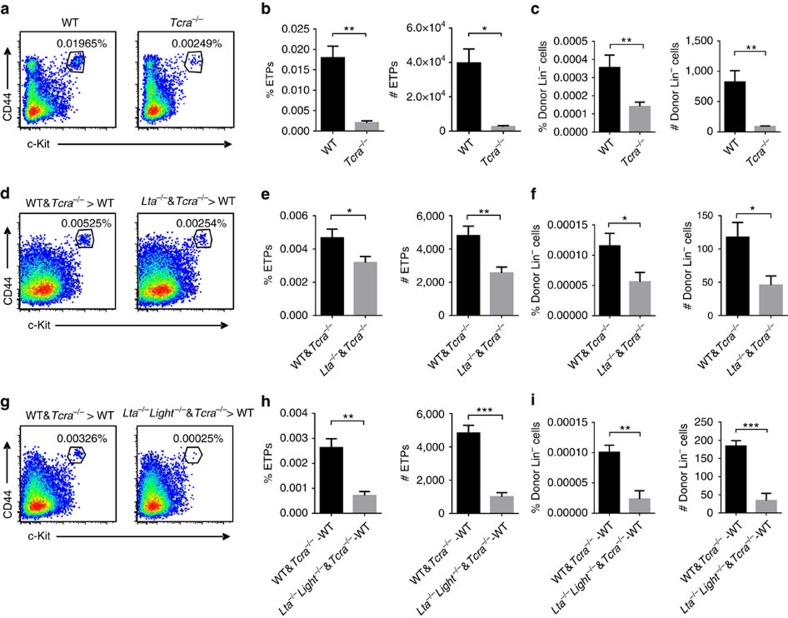
T cells deliver LTβR signalling to control thymic ETP population and progenitor homing. (**a**,**b**) Flow cytometric analysis of ETPs (Lin^−^CD25^−^CD44^+^c-Kit^+^) in WT and *Tcra*^−*/*−^ mice. (**a**) Representative dot plots are shown. (**b**) The graphs display the statistical analysis of the frequency and number of ETPs among total thymocytes. (**c**) Short-term thymic homing assay in WT and *Tcra*^−*/*−^ mice. The frequency and number of donor-derived lineage-negative cells among total thymocytes were analysed by flow cytometry. (**d**,**e**) Flow cytometric analysis of ETPs (Lin^−^CD25^−^CD44^+^c-Kit^+^) in WT and *Tcra*^−*/*−^, and *Lta*^−*/*−^ and *Tcra*^−*/*−^ mixed bone marrow chimeric mice. (**d**) Representative dot plots are shown. (**e**) The graphs display the statistical analysis of the frequency and number of ETPs among total thymocytes. (**f**) Short-term thymic homing assay in WT and *Tcra*^−*/*−^, and *Lta*^−*/*−^ and *Tcra*^−*/*−^ mixed bone marrow chimeric mice. The frequency and number of donor-derived lineage-negative cells among total thymocytes were analysed by flow cytometry. (**g**,**h**) Flow cytometric analysis of ETPs (Lin^−^CD25^−^CD44^+^c-Kit^+^) in WT and *Tcra*^−*/*−^, and *Lta*^−*/*−^*Light*^−*/*−^ and *Tcra*^−*/*−^ mixed bone marrow chimeric mice. (**g**) Representative dot plots are shown. (**h**) The graphs display the statistical analysis of the frequency and number of ETPs among total thymocytes. (**i**) Short-term thymic homing assay in WT and *Tcra*^−*/*−^, and *Lta*^−*/*−^*Light*^−*/*−^ and *Tcra*^−*/*−^ mixed bone marrow chimeric mice. The frequency and number of donor-derived lineage-negative cells among total thymocytes were analysed by flow cytometry. The data are representative of at least two independent experiments with three or more mice per group in each experiment. Error bars represent s.e.m. Asterisks mark statistically significant difference (*P<0.05, **P<0.01 and ***P<0.001 determined by two-tailed unpaired Student's *t*-test).

T cells are another major type of cells expressing both LT and LIGHT. We next wondered whether T cells may be the deliverers of LTβR signalling. A previous study suggested that a lack of a peripheral lymphocyte pool, possibly T cells, may provide feedback control of thymic progenitor cell homing indirectly via the regulation of P-selectin and CCL25 expression in the thymus by sphingosine 1-phosphate^23^. However, this model does not explain why *Rag1*^−/−^ mice also have reduced thymic HPC homing efficiency^23^. Interestingly, we found that *Tcra*^−/−^ mice, in which thymocyte development stops at the double-positive stage, also demonstrated a markedly reduced thymic ETP population compared with WT control mice (Fig. 3a, b). This is also consistent with the reduced ETP population as reported in *Zap70*^−/−^ mice^27^,^28^, which are also deficient for thymocyte positive selection. The short-term homing assay confirmed the impaired thymic homing defect in *Tcra*^−/−^ mice (Fig. 3c). Together, these results indicate a new mechanism of thymic progenitor cell homing mediated by positively selected T cells probably within the thymus.

To determine whether T cells may directly deliver LTβR ligands for thymic ETP maintenance and thymic progenitor cell homing, we generated bone marrow chimeric mice with mixed WT:*Tcra*^−/−^ or *Lta*^−/−^:*Tcra*^−/−^ bone marrow cells (at a ratio of 1:1). Thus, all T cells since the single-positive stage lack LT expression in the chimeric mice generated with *Lta*^−/−^:*Tcra*^−/−^ mixed bone marrow cells. When thymic ETPs were evaluated in these mice 10 weeks later, a significant defect was found (Fig. 3d,e). A short-term homing assay also confirmed the defective thymic progenitor homing in these mice (Fig. 3f). To further investigate the coordination between LT and LIGHT on T cells, we also made bone marrow chimeric mice similar to those described above, but with *Lta*^−/−^*Light*^−/−^ instead of *Lta*^−/−^ bone marrow. A more marked decrease in the thymic ETP population and in homing efficiency was found when both LT and LIGHT were lacking on single-positive T cells (Fig. 3g–i). Since largely comparable thymic ETP defect was found in the T-*Lta*^−/−^*Light*^−/−^ in bone marrow chimeric scenario and in the straight *Lta*^−/−^*Light*^−/−^ mice at steady state (Fig. 2e–g), we suspect that the major role of LT/LIGHT on thymic ETP is likely independent of the bone marrow chimeric condition. In addition, these data indicate that single-positive T cells, but not other cells, are the major deliverer of LT/LIGHT signalling. Thus, positively selected T cells may directly control thymic ECs via LT/LIGHT-coordinated signals during thymic progenitor cell homing. The contribution of different LTβR ligands and types of cells on thymic ETP population control is summarized in Supplementary Table 1.

### The Ly6C^−^Selp^+^ thymic ECs are specialized for thymic homing

We next explored how the T-cell-mediated LTβR signalling pathway may control thymic ECs during HPC homing. The expression of the known molecules involved in thymic progenitor cell homing was first evaluated. However, quantitative PCR with reverse transcription analysis demonstrated comparable expression levels of P-selectin, VCAM-1 and ICAM-1 in purified thymic ECs from WT and *Ltbr*^−/−^ mice (Supplementary Fig. 6a). Normal thymic vasculature and a normal EC population were also preserved in *Ltbr*^−/−^ mice (Supplementary Fig. 6b). Thus, LTβR appears not to primarily control the development and function of thymic ECs.

**Figure 4:**
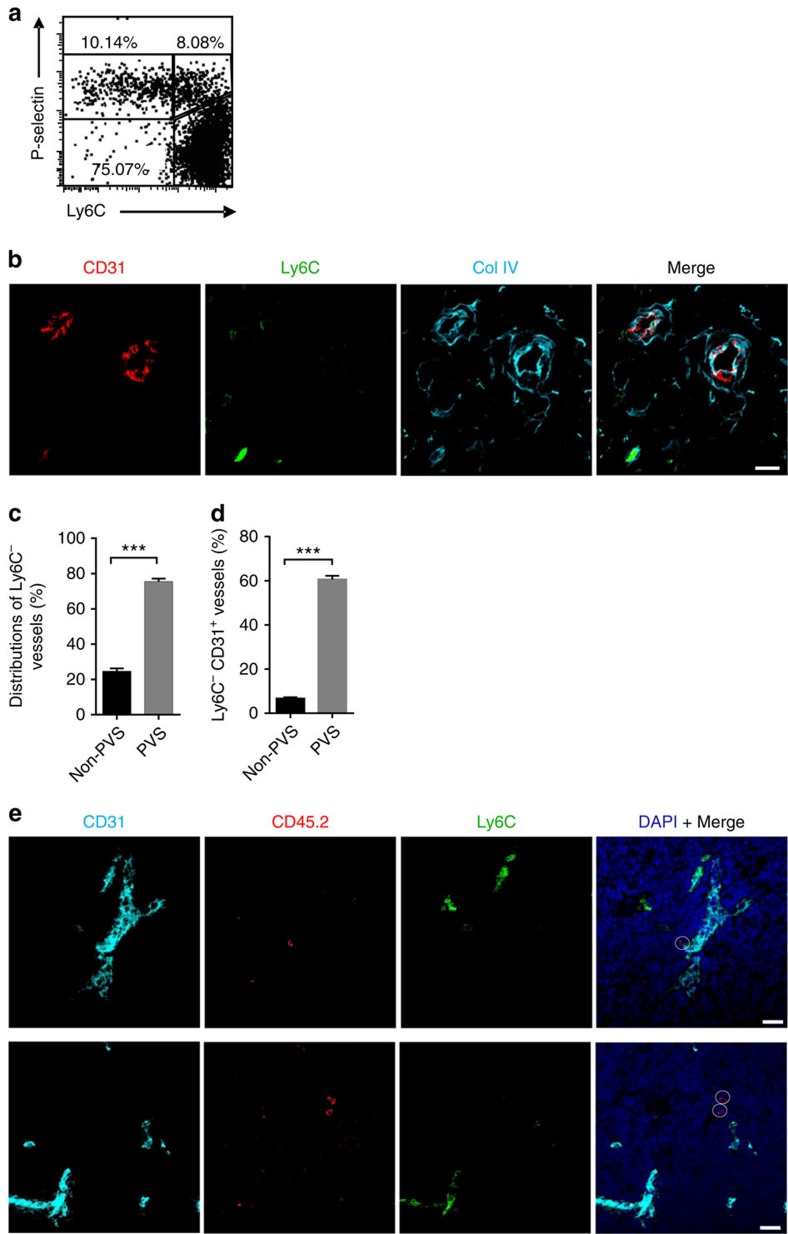
Ly6C^−^Selp^+^ thymic ECs are the specialized population associated with perivascular spaces and immigrating progenitor cells. (**a**) Flow cytometric analysis of P-selectin and Ly6C expression on thymic ECs from WT mice, gated on the CD45^−^EpCAM^−^CD31^+^ cell population, as shown in Supplementary Fig. 7a. (**b**) Immunofluorescence analysis of Ly6C (green) expression on CD31^+^ (red) thymic ECs associated with perivascular spaces, which were defined with double-basement membrane staining of collagen IV (cyan). Scale bars, 20 μm. (**c**) Statistical analysis of the distribution of Ly6C^−^CD31^+^ thymic ECs in PVS and non-PVS areas. (**d**) Statistical analysis of the percentages of PVS or non-PVS associated with Ly6C^−^CD31^+^ thymic ECs. (**e**) Representative images showing the location of thymic seeding progenitor cells (CD45.2^+^, circled red) closer to Ly6C^−^ vessels. Scale bars, 20 μm. The data are representatives of at least three independent experiments (**a**,**b**,**e**); for statistical analysis (**c**,**d**), >40 fields (× 100) containing at least six non-PVS- or PVS-associated vessels from six WT mice were included for analysis. Error bars represent s.e.m. Asterisks mark statistically significant difference (***P<0.001 determined by two-tailed unpaired Student's *t*-test).

The corticomedullary junction has been long recognized as the major place for both thymic homing and egress^8^,^9^,^10^,^11^,^12^,^29^. More specifically, PVSs, compartmentalized by double-basement membranes usually around large blood vessels, have been suggested as critical structures for cellular interchange between the thymus and the blood circulation^11^,^29^. PVS-associated thymic ECs have thus long been suggested to be the key specialized ECs controlling thymic homing. We wondered whether LTβR may control the differentiation of these specialized thymic ECs as it does for HECs in LNs. Because P-selectin^+^ thymic ECs do not seem to only localize in the PVS area, where thymic homing mainly occurs^16^, we hypothesized that a subset of ‘mature' P-selectin^+^ thymic ECs may exist associated with PVSs and be controlled by LTβR. However, the specialized PVS-associated ECs are thus far not well characterized. By analogy, this special population of thymic ECs may possess post-capillary features and share some common characteristics with HECs^30^. Ly6C has been reported as a negative EC differentiation marker that is also reduced on HECs compared with the capillary ECs in the LNs^30^. In the thymus, a subset of P-selectin^+^ thymic ECs (CD31^+^CD45^−^EpCAM^−^) were indeed found to be Ly6C^−^ (Fig. 4a, Supplementary Fig. 7a). The population of CD31^+^CD45^−^EpCAM^−^ cells is mostly blood vascular ECs and are unlikely to contain lymphatic ECs or fibroblasts because they are podoplanin-negative (Supplementary Fig. 7b). In fact, LYVE1^+^ lymphatic vessels were rarely detected in cross-sections of adult C57BL/6 mouse thymi^29^. Further characterization of the different subsets of thymic ECs (Ly6C^+^Selp^−^, Ly6C^+^Selp^+^ and Ly6C^−^Selp^+^) by RNA sequencing (RNA-seq) and transcriptome analysis revealed a total of 806 differentially expressed genes between Ly6C^+^Selp^−^ and Ly6C^−^Selp^+^ ECs (Supplementary Table 2). Some of them have been confirmed by quantitative real-time PCR (Supplementary Fig. 8). Further analysis of these differential genes showed striking similarities between Ly6C^−^Selp^+^ ECs and LN HECs, and between Ly6C^+^Selp^−^ ECs and LN capillary ECs (Supplementary Fig. 9a,b). Gene ontology analysis of the differential genes between Ly6C^−^Selp^+^ ECs and other thymic EC subsets also demonstrated their distinct features, similar to the comparison between LN HECs and capillary ECs (Supplementary Fig. 9c,d)^30^. These data suggest the specialization of thymic Ly6C^−^Selp^+^ ECs and their function in thymic cell homing on the transcriptome level.

To further test whether the aforementioned Ly6C^−^Selp^+^ ECs are the portal ECs associated with PVSs in the thymus during progenitor cell homing, immunofluorescence staining was performed to determine their location. The results demonstrated that CD31^+^Ly6C^−^ vessels were enriched (∼60%) in the corticomedullary junction area. Further co-staining with collagen IV showed that these CD31^+^Ly6C^−^ECs were mainly (∼75%) associated with PVSs (Fig. 4b,c). In addition, ∼60% of the PVSs were associated with CD31^+^Ly6C^−^ ECs, while only ∼6% of the non-PVS vessels were Ly6C^−^ (Fig. 4d). To further determine whether the Ly6C^−^ PVSs are where thymic progenitor entry takes place, a short-term thymic homing assay was performed using fluorescence-activated cell sorting (FACS)-sorted Lin^−^c-Kit^+^ bone marrow cells. Immunofluorescence staining revealed that the majority (∼80%, 28 out of 35) of the thymic seeding progenitor cells were closer to the Ly6C^−^ vessels than to the Ly6C^+^ vessels in the corticomedullary junction (Fig. 4e); this placement was not dependent on the volume of the vessel because large vessels that were Ly6C^+^ did not co-localize with thymic seeding progenitor cells (bottom panel of Fig. 4e). Thus, together with the previous finding that PVSs are where thymic progenitor cell entry takes places^11^, these data provide further evidence that Ly6C^−^Selp^+^ thymic ECs are the portal ECs that mediate thymic progenitor cell entry. These thymic ECs were therefore named thymic portal ECs (TPECs).

### LTβR and T cells are required for the development of TPECs

**Figure 5:**
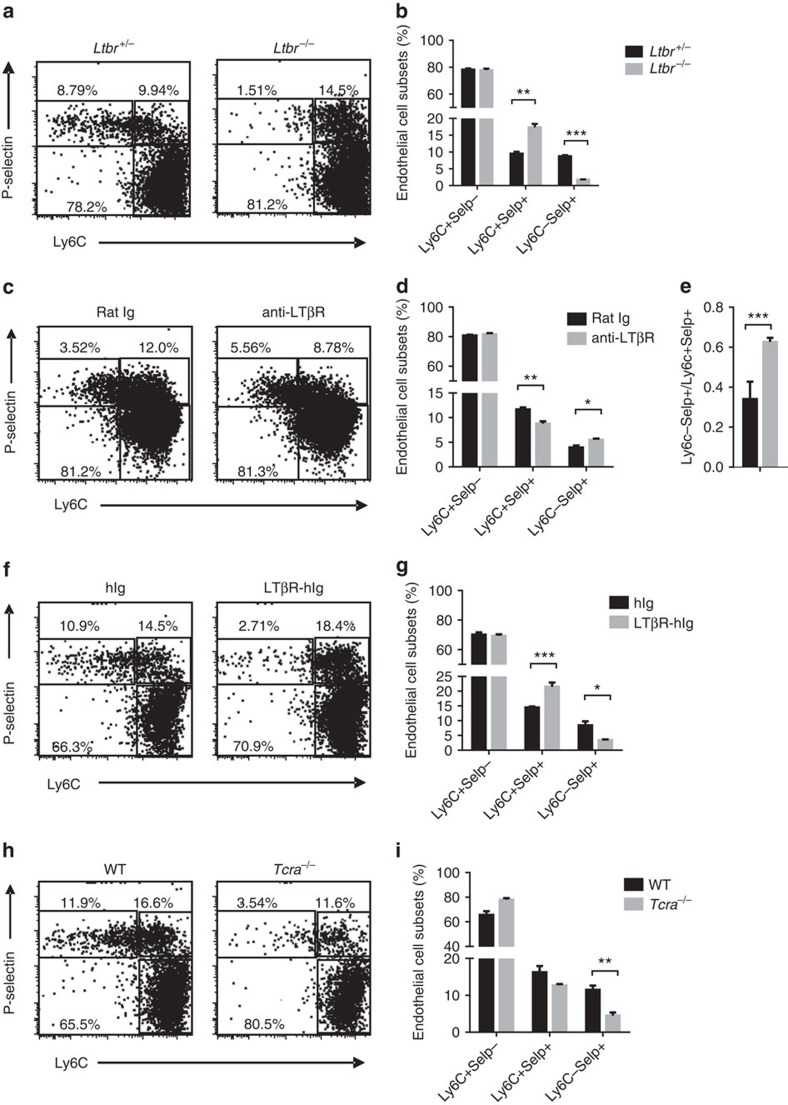
LTβR signalling and T cells are required for the development and homeostasis of Ly6C^−^Selp^+^ thymic ECs. (**a**,**b**) Flow cytometric analysis of the thymic EC subsets in *Ltbr*^−/−^ and littermate control mice. The ECs were gated on the CD45^−^EpCAM^−^CD31^+^ population, as shown in Supplementary Fig. 7a. (**a**) Representative dot plots are shown. (**b**) The graph displays the statistical analysis of the frequency of each endothelial subset. (**c**–**e**) Six-day-old neonatal WT mice were treated with LTβR agonistic antibody (clone 9B10) or control antibody twice with an interval of 5 days between treatments; the thymic EC subsets were analysed by flow cytometry. (**c**) Representative dot plots are shown. The graph displays the statistical analysis of the frequency of each endothelial subset (**d**) and the ratio of Ly6C^−^Selp^+^ and Ly6C^−^Selp^+^ thymic ECs (**e**). (**f**,**g**) Adult WT mice (4–6 weeks old) were treated with LTβR-hIgG or hIgG once a week for 4 weeks; the thymic EC subsets were analysed by flow cytometry. (**f**) Representative dot plots are shown. (**g**) The graph displays the statistical analysis of the frequency of each endothelial subset. (**h**,**i**) Flow cytometric analysis of the thymic EC subsets in WT and *Tcra*^−/−^ mice. The thymic ECs were gated on the CD45^−^EpCAM^−^CD31^+^ population. (**h**) Representative dot plots are shown. (**i**) The graph displays the statistical analysis of the frequency of each endothelial subset. The data are representative of at least two independent experiments with three or more mice per group in each experiment. Error bars represent s.e.m. Asterisks mark statistically significant difference (*P<0.05, **P<0.01 and ***P<0.001 determined by two-tailed unpaired Student's *t*-test).

To test whether LTβR controls the differentiation of TPECs, thymic EC subsets of WT and *Ltbr*^−/−^ mice were analysed by flow cytometry. Strikingly, the population of TPECs was markedly reduced in the absence of LTβR (Fig. 5a,b). Interestingly, the population of Ly6C^+^Selp^+^ thymic ECs was significantly enriched, suggesting a differentiation block and a precursor-progeny or maturation relationship between Ly6C^+^Selp^+^ ECs and TPECs (Fig. 5a,b). Similar changes were confirmed in *Ltbr*^fl/fl^ *Tek*^Cre^ mice (Supplementary Fig. 10a,b), suggesting an endothelial intrinsic function of LTβR signalling in TPEC differentiation/maturation.

To further test whether LTβR signalling constantly regulates TPECs, 6-day-old neonatal WT mice were treated with agonistic LTβR antibody (clone 9B10) or a control antibody. Ten days later, the TPEC population was found to be significantly increased in the 9B10 treatment group compared with that in the control group (Fig. 5c–e). The increase in the TPEC population is likely due to the enhanced differentiation from their precursors because the population of Ly6C^+^Selp^+^ ECs was accordingly reduced. In contrast, LTβR-Ig blockade in adult mice significantly decreased the TPEC population and resulted in the accumulation of Ly6C^+^Selp^+^ ECs within 4 weeks (Fig. 5f,g). Together, these data suggest that LTβR signalling is constantly required for the differentiation and homeostasis of TPECs.

Because positively selected T cells were found to be required for thymic progenitor cell homing (Fig. 3c), we asked whether they are also required for TPEC development. Consistently, the TPEC population was indeed reduced in *Tcra*^−/−^ mice (Fig. 5h,i), further suggesting that positively selected T cells may directly control TPECs for thymic progenitor cell homing.

### LTβR is required for thymic regeneration on injury

**Figure 6:**
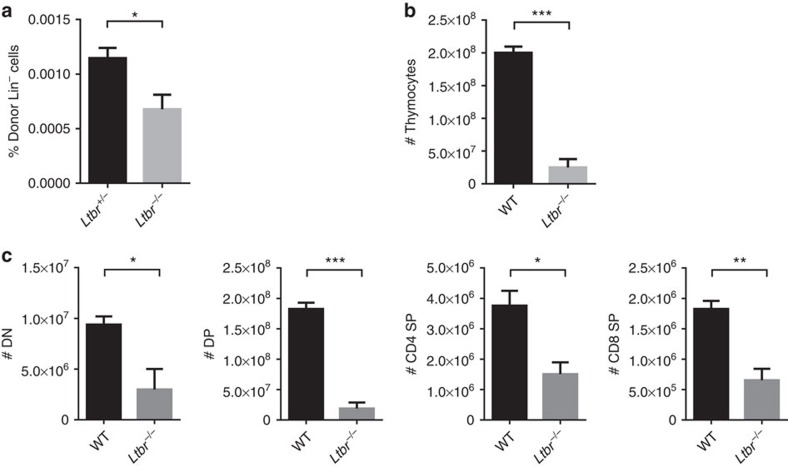
LTβR is required for thymic regeneration upon injury. (**a**) Short-term thymic homing assay in WT and *Ltbr*^−/−^ mice on SL-TBI. The frequency of donor-derived lineage-negative cells was analysed by flow cytometry. (**b**) The total thymic cellularity of WT and *Ltbr*^−/−^ mice 4 weeks after thymic injury induced by SL-TBI. (**c**) The number of developing thymocyte subsets in WT and *Ltbr*^−/−^ mice 4 weeks after thymic injury induced by SL-TBI. The data are representative of at least two independent experiments with three or more mice per group in each experiment. Error bars represent s.e.m. Asterisks mark statistically significant difference (*P<0.05, **P<0.01 and ***P<0.001 determined by two-tailed unpaired Student's *t*-test).

Thymic injury is a common clinical problem and regeneration of the thymus is essential for the re-establishment of the competent naive T-cell compartment^31^. Administration of HPCs or boosting HPC thymic homing has been reported to be able to promote thymic regeneration^4^,^5^,^6^,^7^. To study whether LTβR is required for thymic progenitor cell homing, and thus thymic regeneration, after thymic injury, sublethal total-body irradiation (SL-TBI) was applied to induce thymic injury. A short-term thymic homing assay was performed on day 3 after irradiation. Consistent with the finding in *Ltbr*^−/−^ mice at steady state, a significant reduction in the thymic homing of progenitor cells was found in *Ltbr*^−/−^ mice after SL-TBI (Fig. 6a). To test whether this would result in defective thymic regeneration, the total thymic cellularity and subset distribution were determined 28 days after irradiation. Remarkably, while completely recovered in WT mice, the total cellularity and the number of all major developing thymocyte subsets were markedly reduced in *Ltbr*^−/−^ mice (Fig. 6b, c). Thus, LTβR is required for thymic progenitor homing not only at steady state but also on thymic injury. Deficiency of LTβR resulted in impaired thymic regeneration on SL-TBI-induced injury.

## Discussion

The trafficking of haematopoietic cells to lymphoid tissues is a basic biological process underlying many important immunological functions under both physiological and pathological conditions. In the thymus, the homing of HPCs is critical for normal T-cell development at steady state and for T-cell regeneration on injury. While cell trafficking to secondary lymphoid tissues is well understood, the underlying cellular and molecular mechanisms for thymic homing remain largely unclear. Here we have identified a critical role for the LTβR signalling pathway in the specialized differentiation of thymic ECs (TPECs) for HPC thymic homing and T-cell regeneration.

Our study characterized the PVS-associated TPECs on the transcriptome level for the first time and revealed their high levels of similarity with HECs in peripheral LNs. In fact, the comparison between thymic EC and LN EC subsets suggests highly conserved programmes for EC differentiation and function in different lymphoid tissues. First, LTβR appears to play a critical role in both scenarios. Second, some transcriptional factors related to cardiovascular development, such as Hey1, Id1, Msx1 and Sox17, are all less represented in both TPECs and HECs compared with capillary ECs (Supplementary Table 3). Third, the transmigration machinery may also be shared by TPECs and HECs. Supporting this, Bst1, which encodes CD157, a receptor that has been linked to neutrophil transendothelial migration^32^, is preferentially expressed in both TPECs and HECs (Supplementary Table 3). In contrast, CD97, which may inhibit leukocyte transmigration by strengthening the adherens junctions^33^, was downregulated in both TPECs and HECs (Supplementary Table 3). Our data may provide a useful source for further study on lymphoid vascular differentiation and function, although further validation is still required.

Notably, while significant conservation is found between thymic TPECs and LN HECs, tissue specificity exists. (Supplementary Fig. 9e; Supplementary Table 3). Whether this suggests any selective recruitment of different cells to different organs remains to be investigated. Consistent with this, our data showed that in the short-term homing assay, on bone marrow cell intravenous transfer, the percentage of donor-derived lineage-negative cells among total donor cells enriched about five- to six-fold in the thymus compared with the percentage in the LNs (Supplementary Fig. 11). Even so, it should be noted that the homing selectivity may also be affected by the tissue microenvironment. For example, in the LNs, perivascular CCL19 is transcytosed to the luminal surfaces of HEVs and enables T-cell homing^34^. Similarly, thymic epithelial CCL25 has been suggested to be transcytosed across endothelium to mediated thymic progenitor homing^7^. Further confirmation and subsequent detailed studies on thymic TPEC and LN HEC signature genes may lead to better understanding of the trafficking process in both the thymi and the LNs.

Our data strongly suggest that endothelial LTβR is required for thymic progenitor cell homing and the ETP population. Because TPEC appears to be a critical population for thymic progenitor cell homing and it is highly dependent on LTβR signalling, we hypothesize that LTβR controls this process by promoting thymic EC differentiation/maturation to TPECs. Consistent with this, Ly6C^+^Selp^+^ thymic ECs accumulate in the absence of LTβR. In addition, many TPEC signature genes, such as GlyCAM-1 (refs 17, 22), Enpp2 (refs 35, 36) and VCAM-1 (refs 37, 38), are known to be downstream of LTβR signalling. In fact, one of these genes (that is, VCAM-1) has already been demonstrated to have an important role in thymic progenitor cell homing^15^. The precise downstream mechanism for the LTβR-mediated control of TPEC differentiation/maturation is now an important question.

While LN HECs are mainly controlled by DCs, our study found that positively selected T cells instead control thymic TPEC differentiation for progenitor cell homing. A previous study has suggested an important role of the peripheral T-cell pool in the feedback control of the thymic endothelium^23^. However, it remains elusive how a small change of sphingosine 1-phosphate in the plasma could influence thymic endothelial behaviour^39^. In addition, the model does not explain the decreased thymic progenitor cell receptivity in *Rag1*^−/−^, *Tcra*^−/−^ or *Zap70*^−/−^ mice, all of which severely lack peripheral T cells, suggesting an additional mechanism. Our data presented here indicate a new model, where single-positive T cells may directly control TPECs via intrathymic LTβR signalling for thymic progenitor cell homing. Even so, it remains unclear which population of T-cell controls. The mature single-positive thymocytes have been reported to traffic through PVSs in the corticomedullary junction area during their egress^11^,^29^. In addition, the recirculating T cells from periphery are also speculated to migrate back to the thymus through the corticomedullary junction area^40^. Therefore, both populations are possible candidates. It would be an intriguing question to test in future whether thymic progenitor cell homing is regulated by egressing mature thymocytes or recirculating T cells, indicating either a thymus intrinsic or a thymus extrinsic manner, respectively.

Although thymic ETP population is markedly reduced in adult *Ltbr*^−/−^ mice, the total thymic cellularity is largely comparable to WT mice^41^. This is likely due to the compensational proliferation of T cells at later developmental stages. However, at neonatal stage, significant reduction of total thymic cellularity and number of ETPs were consistently found in *Ltbr*^−/−^ mice (Supplementary Fig. 12a,b). This is in line with the previous finding that the defect of embryonic thymic progenitor colonization resulted in smaller thymic size^42^. However, it is worth to note that the underlying mechanism of LTβR controlling thymic progenitor cell co-lonization at embryonic and postnatal stage may be different. At the embryonic stage, when thymic vascularization is less developed, thymic epithelial cells appear to play a major role for recruitment of progenitor cells directly via CCL21 and CCL25 or indirectly via ephrin B signalling^43^,^44^. LTβR may control embryonic thymic progenitor co-lonization through thymic epithelial cells. In fact, previous work, including ours, have shown that CCL21 expression on thymic epithelial cells is regulated by LTβR signalling^45^,^46^. In the adult mice, LTβR expression on thymic epithelial cells seems to play only minor role compared with endothelial LTβR. The thymic ETP population in *Ltbr*^fl/fl^ K14^Cre^ is only partially reduced compared with the littermate controls (Supplementary Table 1).

Thymic injuries are common phenomena during various pathological conditions^47^. Due to the important role of T cells in adaptive immunity to combat infections and tumours, boosting thymic regeneration is highly desired in the clinic. Numerous methods targeting different stages and different cellular components during T-cell development have been tested in preclinical or clinical studies^47^,^48^,^49^,^50^. Most of these methods aim to expand the population of HPCs or thymocytes/T cells directly or indirectly via thymic stromal cells. Because thymic homing is a limiting factor for T-cell regeneration during bone marrow transplantation^5^, it has recently become a new targeting process for boosting thymic regeneration^7^. In our study, deficiency of LTβR resulted in significantly impaired thymic regeneration on SL-TBI-induced injury, associated with defective thymic progenitor cell homing. Since thymic progenitor replenishment has been shown important for thymic regeneration on injury, the defective thymic progenitor homing defect in *Ltbr*^−/−^ mice is likely a significant contributing factor leading to the defective thymic regeneration^4^,^5^,^6^, although we cannot formally rule out other possibilities. Another important contributing factor for thymic regeneration is thymocyte expansion, which is actually the targeting step for most current strategies for thymic regeneration, as mentioned above. However, so far, there is no indication that LTβR regulates this step. Marked thymocyte expansion occurs at thymic cortex, where cortical epithelial cells play an important role^51^,^52^. However, LTβR seems not to affect cortical epithelial cells, while it is required for medullary epithelial cell development and function according to many studies including ours^53^,^54^. Further evaluation and investigation of the detailed mechanisms may be necessary for proper targeting LTβR signalling pathway for improving immune reconstitution.

In summary, we have uncovered a new mechanism governing thymic EC differentiation to promote thymic progenitor homing and T-cell regeneration. In addition, given the increasingly acknowledged importance of the thymic homing of various haematopoietic cells (including DCs, T-regulatory cells and B cells) for T-cell development and central tolerance^3^,^55^,^56^,^57^,^58^,^59^, our characterization of thymic ECs and identification of their regulation by the LTβR signalling pathway may shed new light to improve our understanding of the general thymic homing process and provide novel strategies for improving immune reconstitution.

## Methods

### Mice

WT C57BL/6 mice were purchased from Vital River, a Charles River company in China. *Tek*-Cre mice were obtained from Nanjing Biomedical Research Institute. *Tcra*^−/−^, CD11c-DTR, *K14*-Cre and CD45.1 mice were obtained from The Jackson Laboratory. *Ltbr*^−/−^, *Ltbr*^fl/fl^ and *Light*^−/−^ mice were as previously described^60^. μMT mice were provided by Hai Qi (Tsinghua University, China) and Baidong Hou (Institute of Biophysics, Chinese Academy of Sciences). *Lta*^−/−^ mice were provided by Burkhard Ludewig (Kantonal Hospital, Switzerland). Five- to seven-week-old sex-matched mice were used unless described otherwise. All mice are on the C57BL/6 background and were maintained under specific pathogen-free conditions with approval by the institutional committee of the Institute of Biophysics, Chinese Academy of Sciences.

### Isolation of thymic ECs

Thymus tissues were digested with 0.2 mg ml^−1^ collagenase I (Sigma), 1 U ml^−1^ dispase I (Corning) and 0.06 mg ml^−1^ DNase I (Roche) in RPMI 1640 medium with 2% fetal bovine serum (FBS) for 1 h at 37 °C. The digestion was washed with cold PBS and filtered through a 70-μm cell strainer (Biologix Group). The stromal cells were enriched by discontinuous density gradient centrifugation in Percoll (GE Healthcare; bottom layer=1.115 g ml^−1^; middle layer=1.065 g ml^−1^; top layer=2% FBS RPMI 1640). The cells recovered from the upper interface were washed and stained with antibodies for flow cytometric analysis or cell sorting.

### Immunofluorescence microscopy

Thymus tissues were embedded in OCT compound and snap-frozen in liquid nitrogen. Ten-micrometre-thick cryosections were air-dried and fixed for 10 min in cold acetone. The cryosections were blocked for 1 h in PBS containing 2% FBS and 1 mg ml^−1^ anti-FcγRII/CD16 (2.4G2; in-house production). The cryosections were incubated overnight at 4 °C with the following antibodies at 2.5 μg ml^−1^ working concentration: anti-CD31 (MEC13.3; eBioscience), anti-Ly6C (HK1.4; BioLegend) and anti-CD45.2 (104; eBioscience). Anti-collagen IV (LSL-LB-1407; Cosmo Bio Co., LTD) was diluted 1:1,000. Unconjugated antibodies were detected with the following secondary antibodies: AlexaFluor594-conjugated goat anti-rabbit IgG (Jackson, ZF-0516) and TRITC-conjugated streptavidin (Jackson, 016-020-084). The detailed information of these antibodies is also listed in Supplementary Table 4. Microscopical analysis was performed using a confocal microscope (Zeiss LSM-710) and the images were processed with ZEN 2010 software (Carl Zeiss, Inc.).

### Flow cytometry and cell sorting

All antibodies used for flow cytometry were from BD Biosciences, eBioscience or BioLegend. The detailed information of antibodies is also listed in Supplementary Table 4. Flow cytometry data were acquired on an LSRFortessa (BD) with FACSDiva software (BD), and FlowJo software (TreeStar) was used for further analysis. Cell sorting was performed on a FACSAriaII or FACSAriaIII (BD). The working concentration of antibodies is 2.5 μg ml^−1^. For ETP analysis, single-cell suspensions (5 × 10^6^ cells in 100 μl FACS buffer) were stained with a lineage marker mix (anti-CD4 (GK1.5), anti-CD8 (53-6.7), anti-CD11b (M1/70), anti-Ter-119 (Ter-119), anti-Gr-1 (RB6-8C5) and anti-B220 (RA3-6B2)), anti-CD44 (IM7), anti-CD25 (PC61) and anti-c-Kit (2B8). For short-term homing analysis, single-cell suspensions (1 × 10^7^ cells in 200 μl FACS buffer) were stained with the lineage marker mix (anti-CD4, anti-CD8, anti-B220, anti-Gr-1, anti-CD11b, anti-CD11c (N418), anti-NK1.1 (PK136) and anti-Ter-119) and stained for the congenic markers CD45.1 (A20) and CD45.2 (104) when the donor cells were not labelled with carboxyfluorescein succinimidyl ester (CFSE). For thymic EC staining, the samples were stained with antibodies against CD45 (30-F11), CD31 (MEC13.3), EpCAM (G8.8), P-selectin (RB40.34) and Ly6C (HK1.4). Dead cells were excluded by staining with propidium iodide (Sigma).

### Bone marrow chimeras and short-term homing assay

For the bone marrow chimeras, 5 × 10^6^ bone marrow cells from donor mice were injected intravenously into congenic C57BL/6 host mice that had been lethally irradiated (1,000 rad). The chimeras were given prophylactic water-containing antibiotics for 4 weeks following bone marrow transfer. The chimeras were analysed 6–10 weeks after transplantation. For the short-term thymic homing assay, congenically marked bone marrow cells were injected intravenously into the mice (5 × 10^7^ cells per mouse of which ∼2% are lineage-negative progenitors). Forty-eight hours later, the thymic cells were collected, stained and analysed by flow cytometry as described above to determine the percentage and number of donor-derived thymic seeding progenitor cells. To directly visualize the thymic seeding progenitor cells, CD45.2^+^Lin^−^c-Kit^+^ bone marrow progenitor cells were sorted with purity of ∼98% and injected intravenously into the CD45.1^+^ recipients (0.5–2 × 10^6^ cells per mouse). Twenty hours later, the recipient mice were killed and the thymi were removed and frozen in OCT for immunofluorescence staining.

### Quantitative real-time PCR

RNA from sorted thymic ECs was extracted using an RNeasy mini kit (Qiagen) according to the manufacturer's instructions. The quality and quantity of the total RNA was assessed using a Nanodrop spectrophotometer (ND 2000C; Thermo Fisher Scientific). The total RNA was reverse-transcribed using a RevertAid First Strand cDNA Synthesis Kit (Fermentas) according to the manufacturer's instructions. The primers used are listed in Supplementary Table 5. Quantitative real-time PCR was performed using SYBR Premix Ex Taq mix (Takara) and the reactions were run on a real-time PCR system (7500, Applied Biosystems). The relative messenger RNA expression levels were calculated using 7500 software v2.0.6 (Applied Biosystems).

### Whole-transcriptome sequencing and analysis

Subsets of thymic ECs were sorted by FACS as described above. The purity of each sample was at least 90%. RNA was extracted using an RNeasy Plus Micro kit (Qiagen) according to the manufacturer's instructions. The quality and quantity of the total RNA was assessed using a High Sensitivity NGS Fragment Analysis kit (DNF-486-0500). The sequencing library was prepared using the NEBNext Ultra RNA Library Prep Kit for Illumina (NEB). The RNA-seq data were sequenced using a Illumina HiSeq 2500 platform to generate ∼4 million 100 bp paired-end reads for each sample. Low-quality reads and sequencing adapters were trimmed from the sequencing data, and then the clean reads were mapped to the genome (mm9) using Tophat. The gene expression level was calculated using Cufflinks and the differential gene expression between samples was calculated using Cuffdiff software. The Pearson correlation of the transcriptomes from each sample was calculated using the ‘cor' function with the method ‘pairwise.complete.obs' in the statistical programming language R. Eight hundred and six differentially expressed genes between Ly6C^+^Selp^−^ and Ly6C^−^Selp^+^ ECs were normalized and shown by *Z*-score value. The 806 differentially expressed genes were also normalized and shown by *Z*-score value in the published RNA microarray data (GSE58056)^30^. Gene ontology pathway analysis was performed using David software.

### LTβR agonist treatment and LTβR blockade

For LTβR agonist treatment, the mice were treated with LTβR agonistic antibody (clone 9B10) or a control antibody intraperitoneally twice with an interval of 5 days between treatments (150 μg per mouse each time). Five days after the last treatment, the thymic ECs were prepared and analysed by flow cytometry. For LTβR blockade, WT mice were treated intraperitoneally with LTβR-hIgG or hIgG (150 μg per mouse) once a week for 4 weeks, and then the thymic ECs were prepared and analysed by flow cytometry.

### DC depletion

CD11c-DTR bone marrow chimeras were generated as described above. For systemic DC depletion, 6 weeks after bone marrow transfer, the chimeric mice were given DT (Sigma) intraperitoneally at a dose of 6 ng g^−1^ body weight every 2 days for 4 weeks.

### Thymic regeneration

Mice were given SL-TBI (550 rad) with no haematopoietic rescue. All TBI experiments were performed using a Co-60 γ-irradiation source.

### Statistical analysis

The statistical significance of the differences between sets of data was assessed by a two-tailed unpaired Student's *t*-test unless stated otherwise. The results are expressed as the mean±s.e.m. Differences with a *P* value<0.05 are marked with asterisks. NS, no significant; *P<0.05; **P<0.01; ***P<0.001.

### Data availability

RNA-seq data set was deposited in gene expression omnibus, with an accession number of GSE83114, which is available via the repository's data access request procedures. All other data are available within the article (as figure source data or Supplementary Information files) or from the authors upon a reasonable request.

## Additional information

**How to cite this article:** Shi, Y. *et al*. LTβR controls thymic portal endothelial cells for haematopoietic progenitor cell homing and T-cell regeneration. *Nat. Commun.* 7:12369 doi: 10.1038/ncomms12369 (2016).

## Supplementary Material

Supplementary Information Supplementary Figures 1-12 and Supplementary Tables 1-5

## References

[b1] Peaudecerf L. . Thymocytes may persist and differentiate without any input from bone marrow progenitors. J. Exp. Med. 209, 1401–1408 (2012). 22778388 10.1084/jem.20120845 PMC3420331

[b2] Martins V. C. . Thymus-autonomous T cell development in the absence of progenitor import. J. Exp. Med. 209, 1409–1417 (2012). 22778389 10.1084/jem.20120846 PMC3420332

[b3] Martins V. C. . Cell competition is a tumour suppressor mechanism in the thymus. Nature 509, 465–470 (2014). 24828041 10.1038/nature13317

[b4] Penit C. & Ezine S. Cell proliferation and thymocyte subset reconstitution in sublethally irradiated mice: compared kinetics of endogenous and intrathymically transferred progenitors. Proc. Natl Acad. Sci. USA 86, 5547–5551 (1989). 2501790 10.1073/pnas.86.14.5547 PMC297660

[b5] Zlotoff D. A. . Delivery of progenitors to the thymus limits T-lineage reconstitution after bone marrow transplantation. Blood 118, 1962–1970 (2011). 21659540 10.1182/blood-2010-12-324954 PMC3158723

[b6] Chen B. J., Cui X., Sempowski G. D., Domen J. & Chao N. J. Hematopoietic stem cell dose correlates with the speed of immune reconstitution after stem cell transplantation. Blood 103, 4344–4352 (2004). 14976038 10.1182/blood-2003-07-2534

[b7] Zhang S. L. . Chemokine treatment rescues profound T-lineage progenitor homing defect after bone marrow transplant conditioning in mice. Blood 124, 296–304 (2014). 24876562 10.1182/blood-2014-01-552794 PMC4093685

[b8] Ito T. & Hoshino T. Light and electron microscopic observations on the vascular pattern of the thymus of the mouse. Arch. Histol. Jap. 27, 351–361 (1966). 6008503 10.1679/aohc1950.27.351

[b9] Bearman R. M., Bensch K. G. & Levine G. D. The normal human thymic vasculature: an ultrastructural study. Anat. Rec. 183, 485–497 (1975). 1200406 10.1002/ar.1091830402

[b10] Lind E. F., Prockop S. E., Porritt H. E. & Petrie H. T. Mapping precursor movement through the postnatal thymus reveals specific microenvironments supporting defined stages of early lymphoid development. J. Exp. Med. 194, 127–134 (2001). 11457887 10.1084/jem.194.2.127 PMC2193450

[b11] Mori K., Itoi M., Tsukamoto N., Kubo H. & Amagai T. The perivascular space as a path of hematopoietic progenitor cells and mature T cells between the blood circulation and the thymic parenchyma. Int. Immunol. 19, 745–753 (2007). 17493961 10.1093/intimm/dxm041

[b12] Zhang S. L. & Bhandoola A. Trafficking to the thymus. Curr. Top. Microbiol. Immunol. 373, 87–111 (2014). 23624945 10.1007/82_2013_324

[b13] Zlotoff D. A. . CCR7 and CCR9 together recruit hematopoietic progenitors to the adult thymus. Blood 115, 1897–1905 (2010). 19965655 10.1182/blood-2009-08-237784 PMC2837318

[b14] Krueger A., Willenzon S., Lyszkiewicz M., Kremmer E. & Forster R. CC chemokine receptor 7 and 9 double-deficient hematopoietic progenitors are severely impaired in seeding the adult thymus. Blood 115, 1906–1912 (2010). 20040757 10.1182/blood-2009-07-235721

[b15] Scimone M. L., Aifantis I., Apostolou I., von Boehmer H. & von Andrian U. H. A multistep adhesion cascade for lymphoid progenitor cell homing to the thymus. Proc. Natl Acad. Sci. USA 103, 7006–7011 (2006). 16641096 10.1073/pnas.0602024103 PMC1459009

[b16] Rossi F. M. . Recruitment of adult thymic progenitors is regulated by P-selectin and its ligand PSGL-1. Nat. Immunol. 6, 626–634 (2005). 15880112 10.1038/ni1203

[b17] Browning J. L. . Lymphotoxin-beta receptor signaling is required for the homeostatic control of HEV differentiation and function. Immunity 23, 539–550 (2005). 16286021 10.1016/j.immuni.2005.10.002

[b18] Onder L. . Endothelial cell-specific lymphotoxin-beta receptor signaling is critical for lymph node and high endothelial venule formation. J. Exp. Med. 210, 465–473 (2013). 23420877 10.1084/jem.20121462 PMC3600902

[b19] Lu T. T. & Browning J. L. Role of the lymphotoxin/light system in the development and maintenance of reticular networks and vasculature in lymphoid tissues. Front. Immunol. 5, 47 (2014). 24575096 10.3389/fimmu.2014.00047 PMC3920476

[b20] Zhu M., Yang Y., Wang Y., Wang Z. & Fu Y. X. LIGHT regulates inflamed draining lymph node hypertrophy. J. Immunol. 186, 7156–7163 (2011). 21572030 10.4049/jimmunol.1002097 PMC3110546

[b21] Zhu M. & Fu Y. X. The role of core TNF/LIGHT family members in lymph node homeostasis and remodelling. Immunol. Rev. 244, 75–84 (2011). 22017432 10.1111/j.1600-065X.2011.01061.x

[b22] Moussion C. & Girard J. P. Dendritic cells control lymphocyte entry to lymph nodes through high endothelial venules. Nature 479, 542–546 (2011). 22080953 10.1038/nature10540

[b23] Gossens K. . Thymic progenitor homing and lymphocyte homeostasis are linked via S1P-controlled expression of thymic P-selectin/CCL25. J. Exp. Med. 206, 761–778 (2009). 19289576 10.1084/jem.20082502 PMC2715120

[b24] Baba T., Nakamoto Y. & Mukaida N. Crucial contribution of thymic Sirp alpha+ conventional dendritic cells to central tolerance against blood-borne antigens in a CCR2-dependent manner. J. Immunol. 183, 3053–3063 (2009). 19675159 10.4049/jimmunol.0900438

[b25] Angeli V. . B cell-driven lymphangiogenesis in inflamed lymph nodes enhances dendritic cell mobilization. Immunity 24, 203–215 (2006). 16473832 10.1016/j.immuni.2006.01.003

[b26] Kumar V. . Global lymphoid tissue remodeling during a viral infection is orchestrated by a B cell-lymphotoxin-dependent pathway. Blood 115, 4725–4733 (2010). 20185585 10.1182/blood-2009-10-250118

[b27] Vicente R., Adjali O., Jacquet C., Zimmermann V. S. & Taylor N. Intrathymic transplantation of bone marrow-derived progenitors provides long-term thymopoiesis. Blood 115, 1913–1920 (2010). 20040762 10.1182/blood-2009-06-229724 PMC2837328

[b28] de Barros S. C. . Intrathymic progenitor cell transplantation across histocompatibility barriers results in the persistence of early thymic progenitors and T-cell differentiation. Blood 121, 2144–2153 (2013). 23305740 10.1182/blood-2012-08-447417 PMC3596972

[b29] Zachariah M. A. & Cyster J. G. Neural crest-derived pericytes promote egress of mature thymocytes at the corticomedullary junction. Science 328, 1129–1135 (2010). 20413455 10.1126/science.1188222 PMC3107339

[b30] Lee M. . Transcriptional programs of lymphoid tissue capillary and high endothelium reveal control mechanisms for lymphocyte homing. Nat. Immunol. 15, 982–995 (2014). 25173345 10.1038/ni.2983 PMC4222088

[b31] Lynch H. E. . Thymic involution and immune reconstitution. Trends Immunol. 30, 366–373 (2009). 19540807 10.1016/j.it.2009.04.003 PMC2750859

[b32] Ortolan E. . CD157 plays a pivotal role in neutrophil transendothelial migration. Blood 108, 4214–4222 (2006). 16917007 10.1182/blood-2006-04-017160

[b33] Becker S. . Overexpression of CD97 in intestinal epithelial cells of transgenic mice attenuates colitis by strengthening adherens junctions. Plos ONE 5, e8507 (2010). 20084281 10.1371/journal.pone.0008507 PMC2801611

[b34] Baekkevold E. S. . The CCR7 ligand elc (CCL19) is transcytosed in high endothelial venules and mediates T cell recruitment. J. Exp. Med. 193, 1105–1112 (2001). 11342595 10.1084/jem.193.9.1105 PMC2193428

[b35] Bai Z. . Constitutive lymphocyte transmigration across the basal lamina of high endothelial venules is regulated by the autotaxin/lysophosphatidic acid axis. J. Immunol. 190, 2036–2048 (2013). 23365076 10.4049/jimmunol.1202025

[b36] Kanda H. . Autotaxin, an ectoenzyme that produces lysophosphatidic acid, promotes the entry of lymphocytes into secondary lymphoid organs. Nat. Immunol. 9, 415–423 (2008). 18327261 10.1038/ni1573 PMC2783613

[b37] Dejardin E. . The lymphotoxin-beta receptor induces different patterns of gene expression via two NF-kappaB pathways. Immunity 17, 525–535 (2002). 12387745 10.1016/s1074-7613(02)00423-5

[b38] Mebius R. E. Organogenesis of lymphoid tissues. Nat. Rev. Immunol. 3, 292–303 (2003). 12669020 10.1038/nri1054

[b39] Cyster J. G. Settling the thymus: immigration requirements. J. Exp. Med. 206, 731–734 (2009). 19349460 10.1084/jem.20090458 PMC2715109

[b40] Hale J. S. & Fink P. J. Back to the thymus: peripheral T cells come home. Immunol. Cell Biol. 87, 58–64 (2009). 19030016 10.1038/icb.2008.87 PMC2679673

[b41] Venanzi E. S., Gray D. H., Benoist C. & Mathis D. Lymphotoxin pathway and Aire influences on thymic medullary epithelial cells are unconnected. J. Immunol. 179, 5693–5700 (2007). 17947641 10.4049/jimmunol.179.9.5693

[b42] Liu C. . Coordination between CCR7- and CCR9-mediated chemokine signals in prevascular fetal thymus colonization. Blood 108, 2531–2539 (2006). 16809609 10.1182/blood-2006-05-024190

[b43] Liu C. . The role of CCL21 in recruitment of T-precursor cells to fetal thymi. Blood 105, 31–39 (2005). 15358618 10.1182/blood-2004-04-1369

[b44] Stimamiglio M. A. . EphB2-mediated interactions are essential for proper migration of T cell progenitors during fetal thymus colonization. J. Leukoc. Biol. 88, 483–494 (2010). 20504947 10.1189/jlb.0210079

[b45] Zhu M., Chin R. K., Tumanov A. V., Liu X. & Fu Y. X. Lymphotoxin beta receptor is required for the migration and selection of autoreactive T cells in thymic medulla. J. Immunol. 179, 8069–8075 (2007). 18056347 10.4049/jimmunol.179.12.8069

[b46] Seach N. . The lymphotoxin pathway regulates Aire-independent expression of ectopic genes and chemokines in thymic stromal cells. J. Immunol. 180, 5384–5392 (2008). 18390720 10.4049/jimmunol.180.8.5384 PMC2760078

[b47] Gruver A. L. & Sempowski G. D. Cytokines, leptin, and stress-induced thymic atrophy. J. Leukoc. Biol. 84, 915–923 (2008). 18495786 10.1189/jlb.0108025 PMC2538595

[b48] Velardi E., Dudakov J. A. & van den Brink M. R. Clinical strategies to enhance thymic recovery after allogeneic hematopoietic stem cell transplantation. Immunol. Lett. 155, 31–35 (2013). 24120996 10.1016/j.imlet.2013.09.016 PMC3871183

[b49] Awong G., LaMotte-Mohs R. & Zuniga-Pflucker J. C. Key players for T-cell regeneration. Curr. Opin. Hematol. 17, 327–332 (2010). 20571395 10.1097/MOH.0b013e3283395133

[b50] Seach N., Layton D., Lim J., Chidgey A. & Boyd R. Thymic generation and regeneration: a new paradigm for establishing clinical tolerance of stem cell-based therapies. Curr. Opin. Biotechnol. 18, 441–447 (2007). 17702564 10.1016/j.copbio.2007.07.001

[b51] Takada K., Ohigashi I., Kasai M., Nakase H. & Takahama Y. Development and function of cortical thymic epithelial cells. Curr. Top. Microbiol. Immunol. 373, 1–17 (2014). 23612989 10.1007/82_2013_322

[b52] Petrie H. T. & Zuniga-Pflucker J. C. Zoned out: functional mapping of stromal signaling microenvironments in the thymus. Annu. Rev. Immunol. 25, 649–679 (2007). 17291187 10.1146/annurev.immunol.23.021704.115715

[b53] Akiyama T., Shinzawa M. & Akiyama N. TNF receptor family signaling in the development and functions of medullary thymic epithelial cells. Front. Immunol. 3, 278 (2012). 22969770 10.3389/fimmu.2012.00278 PMC3432834

[b54] Zhu M., Brown N. K. & Fu Y. X. Direct and indirect roles of the LTbetaR pathway in central tolerance induction. Trends Immunol. 31, 325–331 (2010). 20675191 10.1016/j.it.2010.06.005 PMC2933296

[b55] Love P. E. & Bhandoola A. Signal integration and crosstalk during thymocyte migration and emigration. Nat. Rev. Immunol. 11, 469–477 (2011). 21701522 10.1038/nri2989 PMC3710714

[b56] Bonasio R. . Clonal deletion of thymocytes by circulating dendritic cells homing to the thymus. Nat. Immunol. 7, 1092–1100 (2006). 16951687 10.1038/ni1385

[b57] Hadeiba H. . Plasmacytoid dendritic cells transport peripheral antigens to the thymus to promote central tolerance. Immunity 36, 438–450 (2012). 22444632 10.1016/j.immuni.2012.01.017 PMC3315699

[b58] Yamano T. . Thymic B cells are licensed to present self antigens for central T cell tolerance induction. Immunity 42, 1048–1061 (2015). 26070482 10.1016/j.immuni.2015.05.013

[b59] Thiault N. . Peripheral regulatory T lymphocytes recirculating to the thymus suppress the development of their precursors. Nat. Immunol. 16, 628–634 (2015). 25939024 10.1038/ni.3150

[b60] Wang Y. . Lymphotoxin beta receptor signaling in intestinal epithelial cells orchestrates innate immune responses against mucosal bacterial infection. Immunity 32, 403–413 (2010). 20226692 10.1016/j.immuni.2010.02.011 PMC2878123

