# Supplementary material for: LTβR controls thymic portal endothelial cells for haematopoietic progenitor cell homing and T-cell regeneration
Source: Nat Commun. 2016 Aug 5;7:12369. doi: 10.1038/ncomms12369 (PMC4980457; doi:10.1038/ncomms12369)
Supplement: Supplementary Information — Supplementary Figures 1-12 and Supplementary Tables 1-5 [file ncomms12369-s1.pdf]

Supplementary Figure 1.

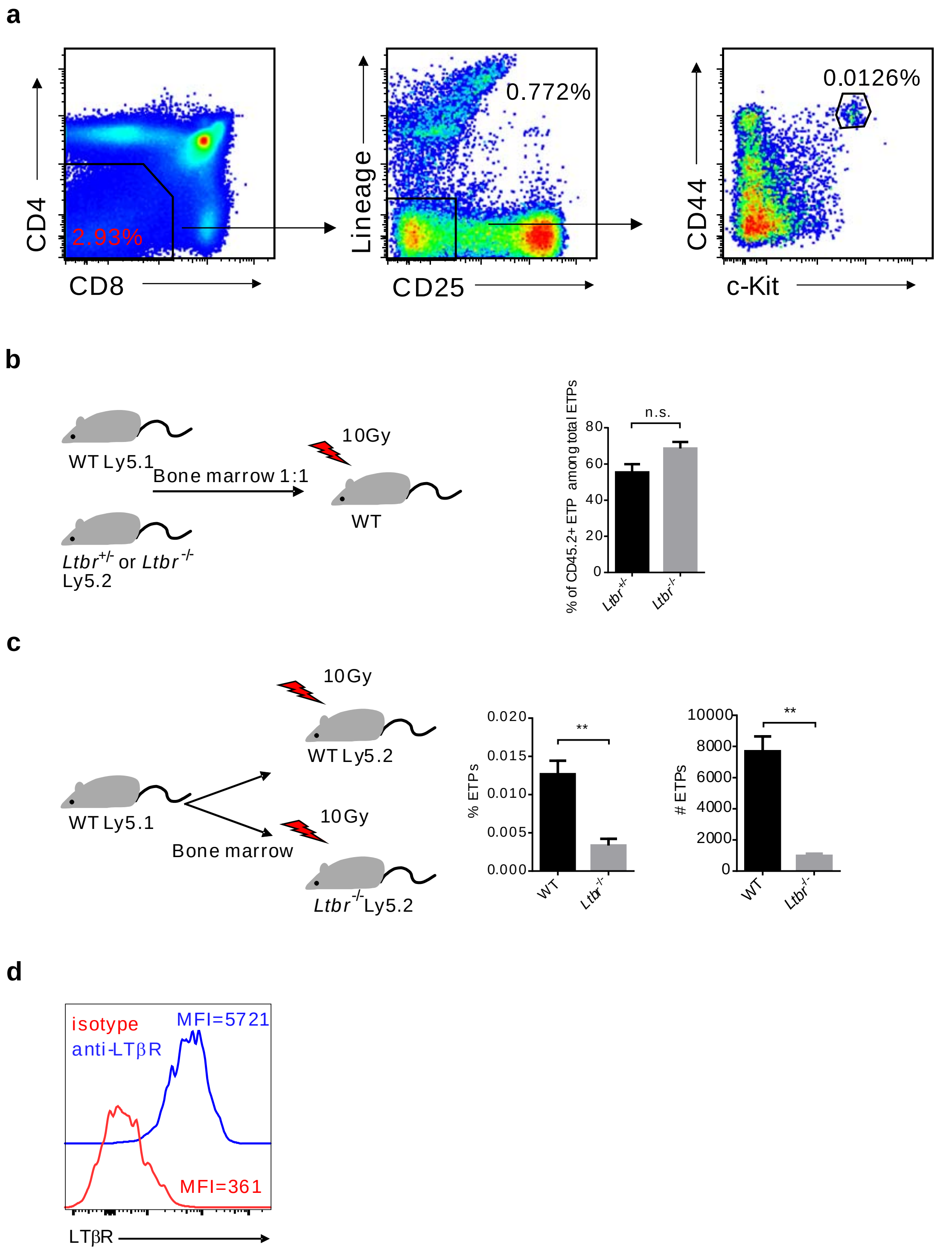

Supplementary Figure 1. Radioresistant cell derived LTβR is required for thymic ETP population.

(**a**) Gating strategy for the detection of thymic ETPs by flow cytometry. FACS plots show the successive gating strategy after standard forward/side scatter and live/dead

gating. A strict forward angle gate was used to exclude doublets.

(**b**) Mixed bone marrow chimeric mice were generated as depicted. 8 weeks later, flow cytometric analysis shows comparable percentages of ETPs derived from *Ltbr*$^{+/-}$ and *Ltbr*$^{-/-}$ bone marrow cells. Results are mean ± SEM (n=6 or 7).

(**c**) Bone marrow chimeric mice were generated as depicted. 8 weeks later, flow cytometric analysis shows dramatically reduced frequency and number of ETP population in *Ltbr*$^{-/-}$ hosts. Results are mean ± SEM (n=3).

(**d**) Thymic tissues were digested as described in Methods. Cells were stained for flow cytometric analysis. The expression of LTβR on total thymic ECs is shown. Representative of two experiments.

Supplementary Figure 2.

a

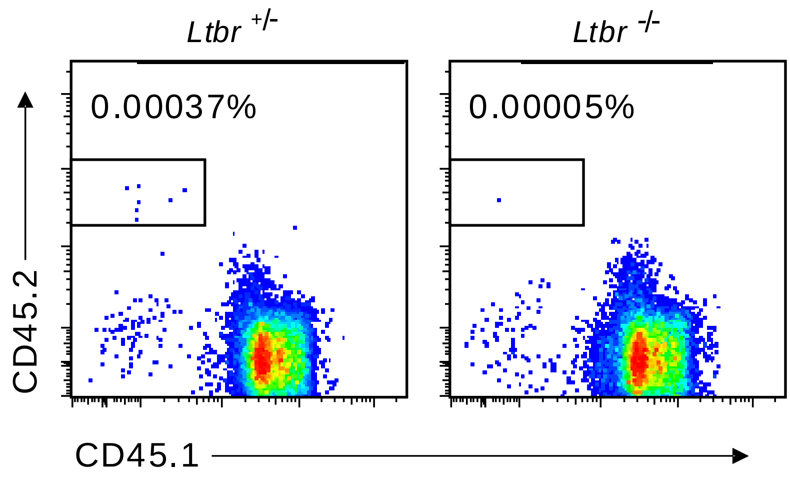

b

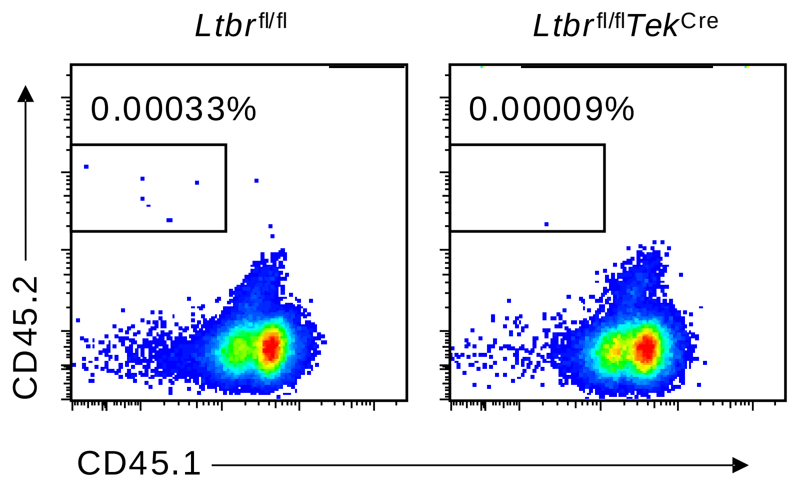

Supplementary Figure 2. Endothelial LTβR controls thymic progenitor homing. (**a, b**) Short-term thymic homing assay in *Ltbr*$^{-/-}$ (**a**), *Ltbr*$^{fl/fl}$ *Tek*^{Cre} (**b**) and littermate control mice. Representative FACS plots are shown. The number indicates the percentage of gated dornor-derived Lin^{-} cells among total thymocytes.

Supplementary Figure 3.

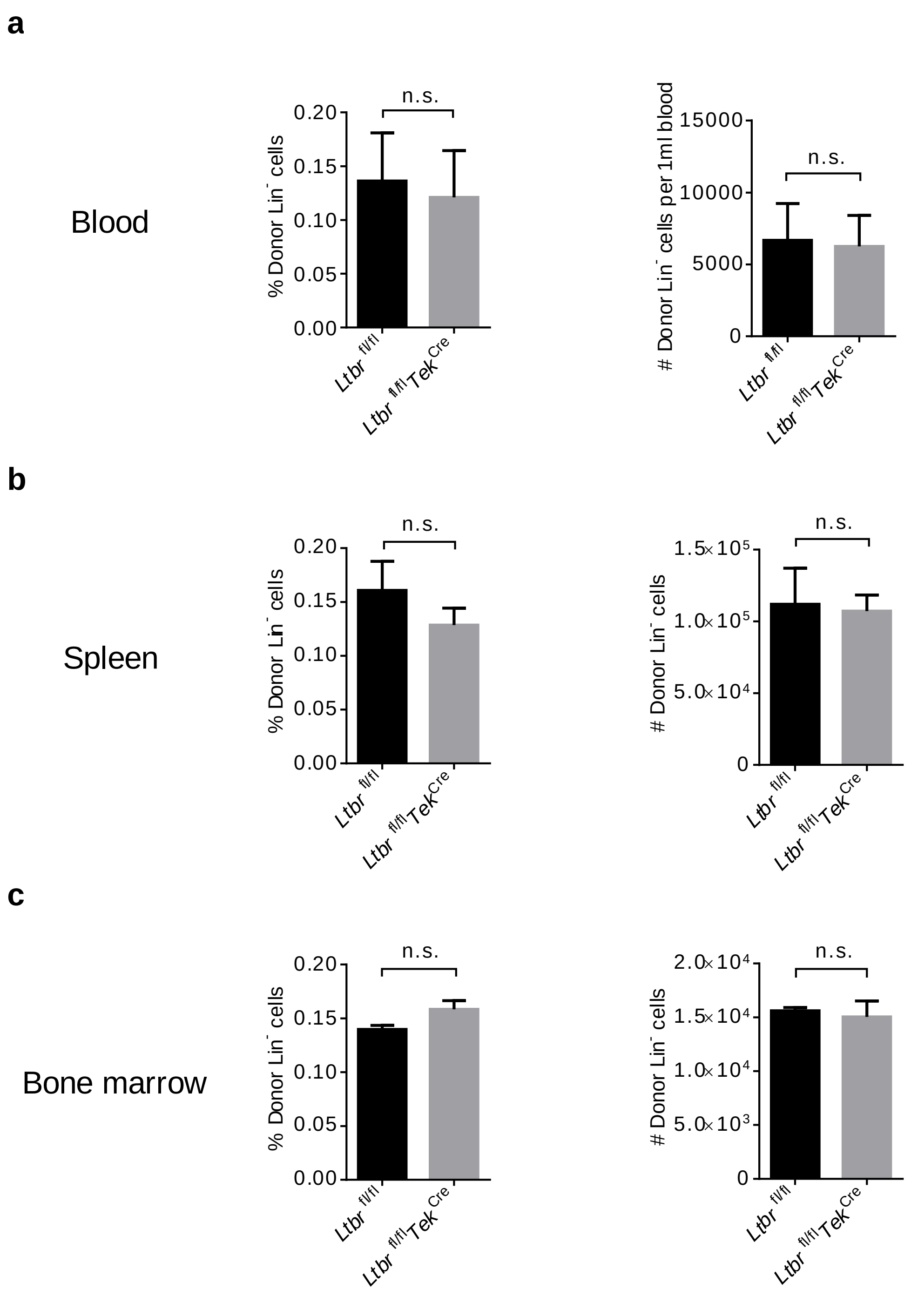

Supplementary Figure 3. Endothelial LTβR deficiency does not influence HPC distribution in the blood, spleen and bone marrow.

(**a, b, c**) Mice of indicated genotypes were injected intravenously with congenically marked bone marrow cells (5×10^7 cells/mouse). Forty-eight hours later, the blood, spleen and bone marrow tissues were collected and cells were stained for flow

cytometric analysis. The percentage and number of donor-derived Lin^- cells in each organ were calculated. (**a**) Blood; (**b**) Spleen; (**c**) Bone marrow. Graphs display the statistical analysis of the frequency (left) and number (right) of ETPs in total spleen, one tibia and femur bone marrow, and 1ml of blood. Results are mean ± SEM (n=3 or 5). Representative of two experiments.

Supplementary Figure 4.

a

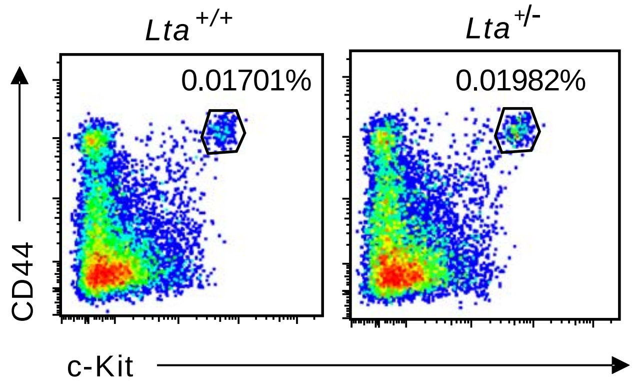

b

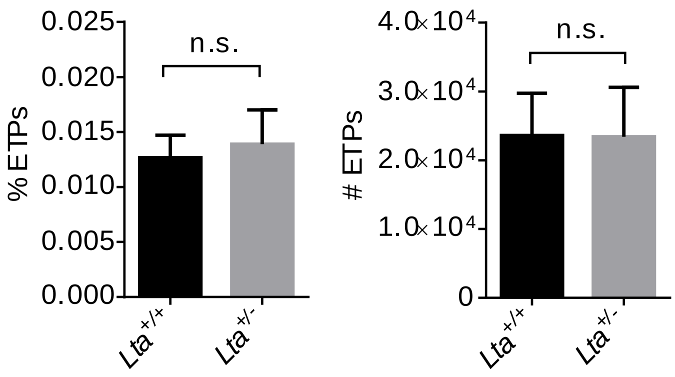

Supplementary Figure 4. Comparable thymic ETP population in *Lta*$^{+/+}$ and *Lta*$^{+/-}$ mice.

(**a, b**) Flow cytometric analysis of ETPs (Lin^{-}CD25^{-}CD44^{+}c-Kit^{+}) in *Lta*$^{+/+}$ and *Lta*$^{-/-}$ mice. (**a**) Representative dot plots are shown. (**b**) The graphs display the statistical analysis of the frequency and number of ETPs among total thymocytes. Results are mean ± SEM (n=7 or 8).

Supplementary Figure 5.

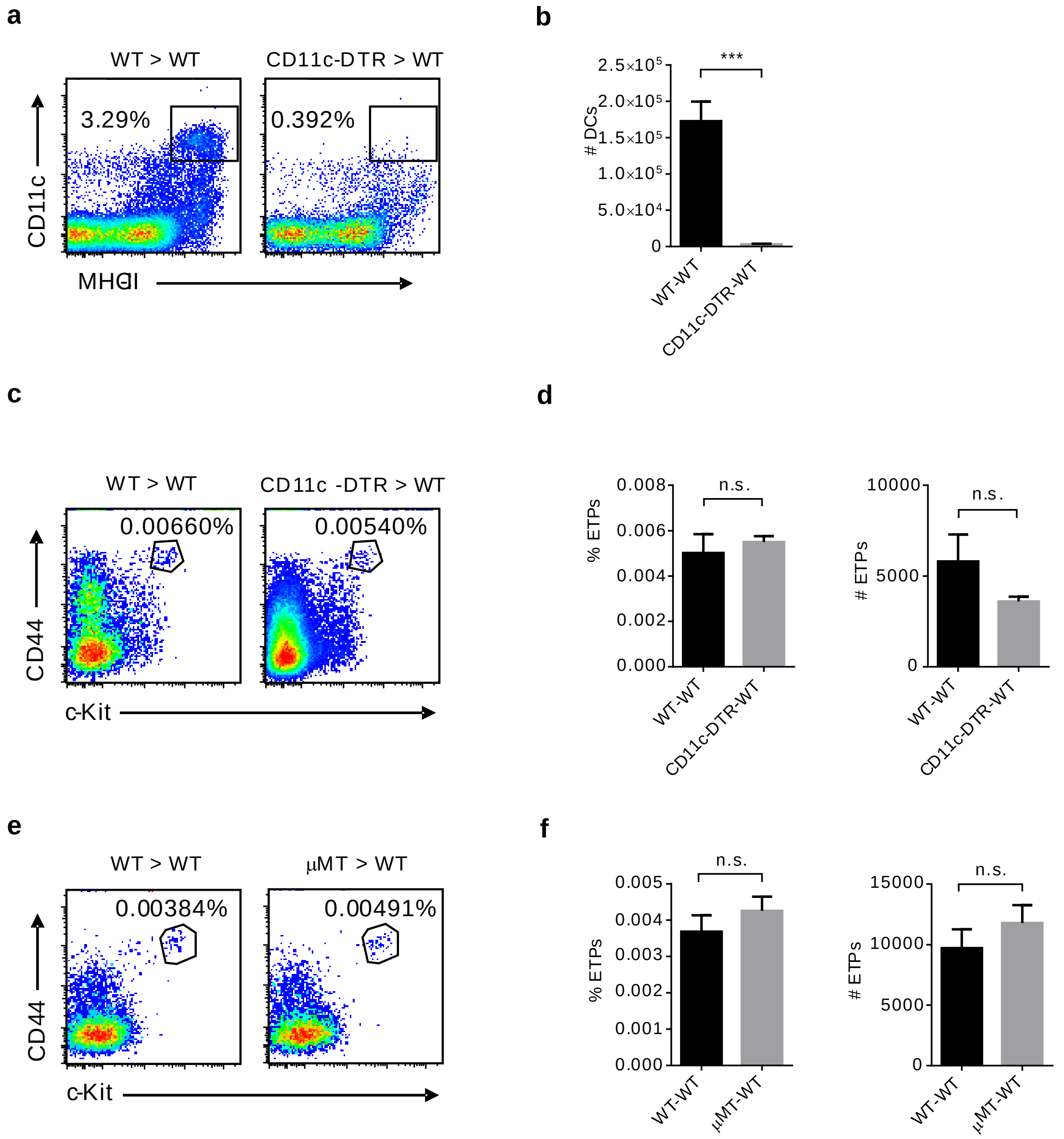

Supplementary Figure 5. DCs and B cells do not control thymic ETP population. (**a, b**) Bone marrow chimeric mice were established in WT mice with WT or CD11c-DTR bone marrow cells. 6 weeks later, mice were treated with diphtheria toxin for 4 weeks. Thymic tissues were digested and cells were stained for flow cytometric analysis. MHC-II^{+}CD11c^{hi} DCs were analyzed. Representative dot plots are shown (**a**). Graphs display the statistical analysis of the number of DCs in total thymus (**b**). Results are mean ± SEM (n= 4).

(**c, d**) Bone marrow chimeric mice were established in WT mice with WT or

CD11c-DTR bone marrow cells. 6 weeks later, mice were treated with diphtheria toxin for 4 weeks before thymic ETP analysis. Representative dot plots are shown (**c**). Graphs display the statistical analysis of the frequency and number of ETPs among total thymocytes (**d**). Results are mean ± SEM (n=4).

(**e, f**) Bone marrow chimeric mice were established in WT mice with WT or μMT bone marrow cells. 8 weeks later, flow cytometric analysis of ETPs was performed. Representative dot plots are shown (**e**). Graphs display the statistical analysis of the frequency and number of ETPs among total thymocytes (**f**). Results are mean ± SEM (n= 3 or 5).

Supplementary Figure 6.

a

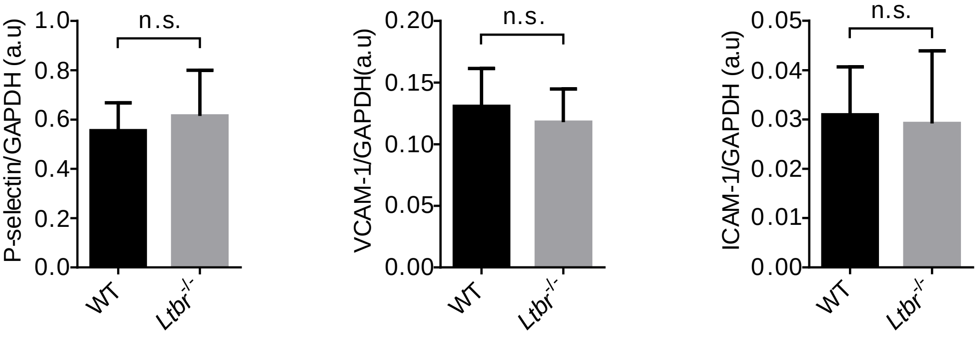

b

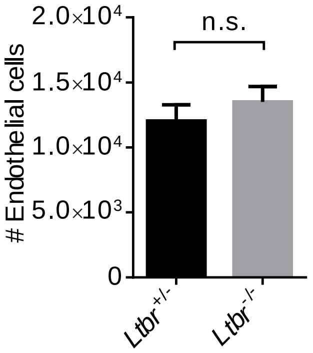

Supplementary Figure 6. Grossly normal endothelial development and function in LTβR deficient mice

(**a**) Quantitative real-time PCR analysis of the expression level of P-selectin, VCAM-1 and ICAM-1 in thymic endothelial cells from WT and *Ltbr*$^{-/-}$ mice. Results are mean ± SEM (n=3).

(b) Normal number of thymic endothelial cells in *Ltbr*$^{-/-}$ mice. Results are mean ± SEM (n=4).

Supplementary Figure 7.

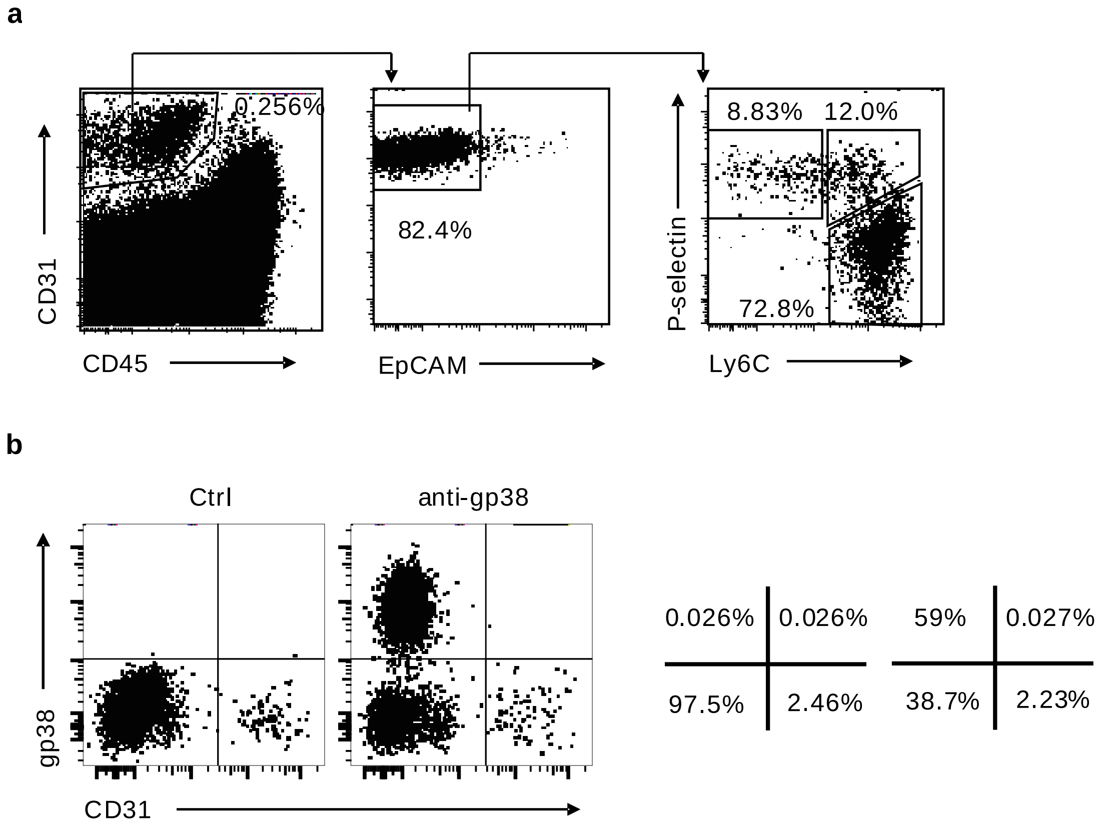

Supplementary Figure 7. Thymic EC characterization by flow cytometry.

(**a**) Gating strategy for the distinction and isolation of Ly6C^{+}Sep^{-}, Ly6C^{+}Selp^{+}, and Ly6C^{-}Selp^{+} thymic EC subsets by flow cytometry. FACS plots show the successive gating strategy after standard forward/side scatter and live/dead gating. A strict forward angle gate was used to exclude doublets.

(**b**) Thymic tissues were digested as described in Methods. Cells were stained for flow cytometric analysis. The expression of podoplanin (gp38) on CD45^{-}EpCAM^{-} thymic stromal cells is shown. Representative of two experiments.

Supplementary Figure 8.

a

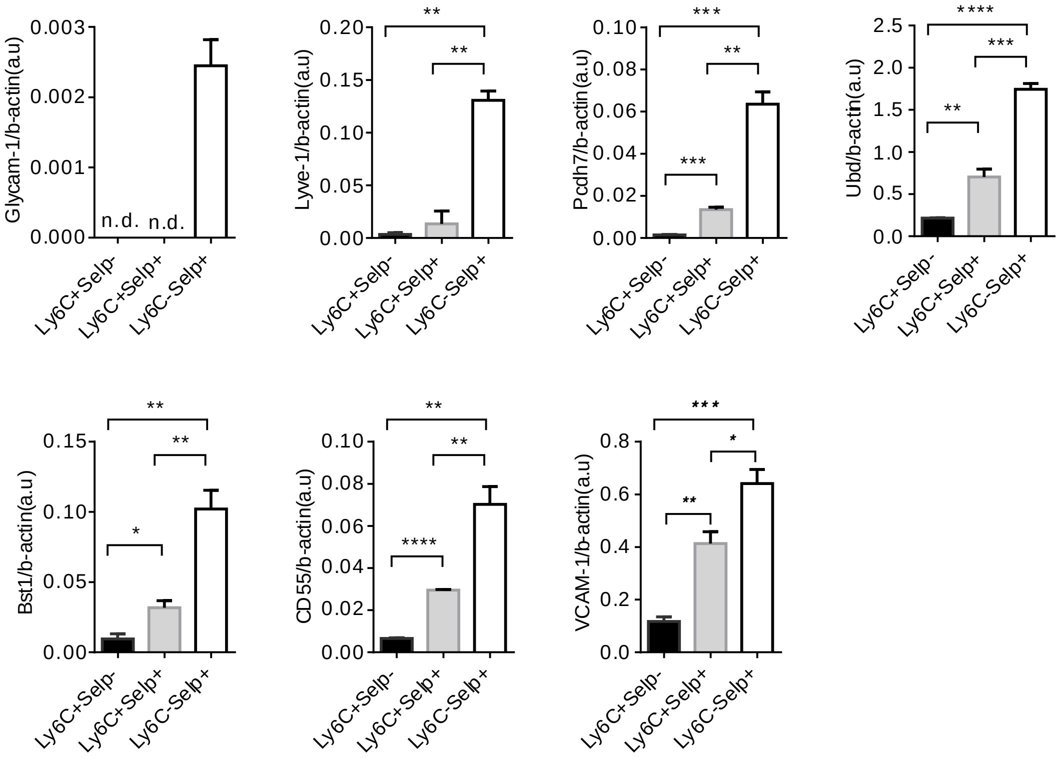

Supplementary Figure 8. Expression of selected genes in thymic EC subsets.

(**a**) Quantitative real-time PCR analysis of the expression of selected HEC signature genes in different subsets of thymic ECs. Results are mean ± SEM of three independent experiments (n=3).

Supplementary Figure 9.

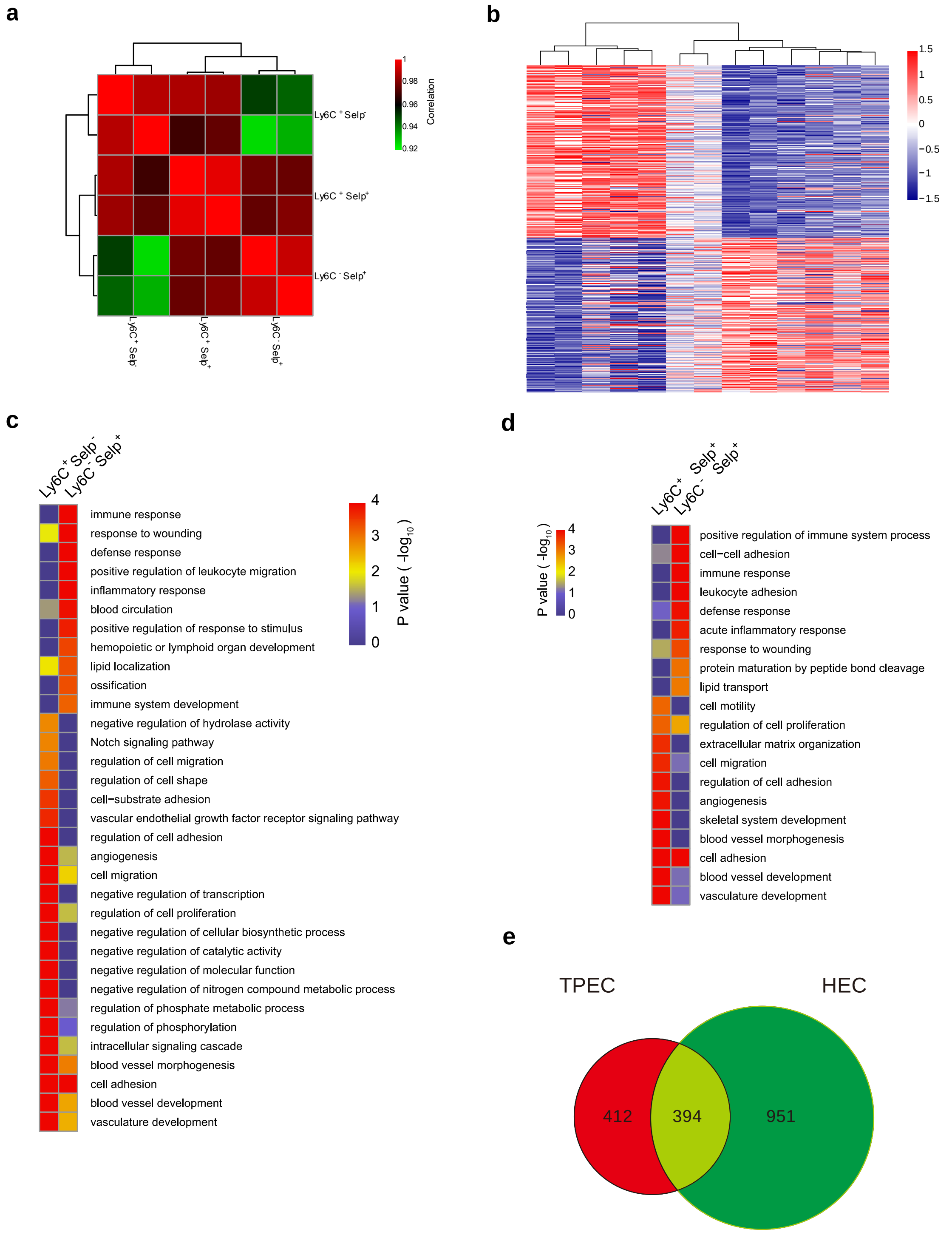

Supplementary Figure 9. Ly6C$^-$Selp$^+$ thymic endothelial cells is a specialized population resembled LN HECs.

(**a**) Unsupervised hierarchical clustering analysis based on the Pearson correlations of transcriptome among samples. Each terminal branch represents the result from a

single sequencing sample.

(**b**) Hierarchical clustering of expression Z-score of sequencing samples (Ly6C$^+$Selp$^-$, Ly6C$^+$Selp$^+$ and Ly6C$^-$Selp$^+$) and published microarray samples (LN HECs and CAP ECs) with the list of genes significantly differentially expressed between Ly6C$^+$Selp$^-$ and Ly6C$^-$Selp$^+$ ECs as determined by Cuffdiff software. Z-score value was used to normalize the expression level of the same gene among sequencing samples and microarray samples, respectively.

(**c, d**) Selected GO terms and pathways (right margin) showing significant enrichment (Benjamini < 0.05, analyzed by DAVID software) in the differential gene sets between Ly6C$^+$Selp$^-$ and Ly6C$^-$Selp$^+$ ECs (**c**), and between Ly6C$^+$Selp$^+$ and Ly6C$^-$Selp$^+$ ECs (**d**). Order and box color are based on −log10 of the corrected P-value.

(**e**) TPECs and HECs share significant number of signature genes.

Supplementary Figure 10.

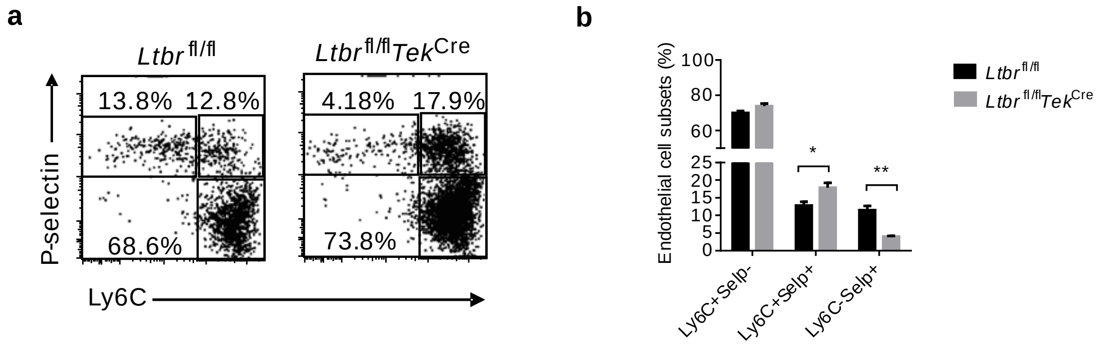

Supplementary Figure 10. Endothelial LTβR signaling is required for the development of Ly6C^{-}Selp^{+} thymic ECs.

(**a, b**) Flow cytometric analysis of thymic EC subsets in *Ltbr*$^{fl/fl}$ *Tek*^{Cre} and their littermate control mice. ECs were gated on CD45^{-}EpCAM^{-}CD31^{+} population. Representative dot plots are shown (**a**). The graph displays the statistical analysis of the frequency of each endothelial subset (**b**). Results are mean ± SEM (n=3).

Supplementary Figure 11.

a

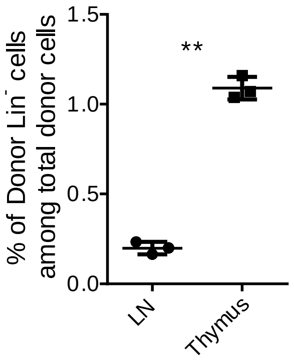

Supplementary Figur e 1 1. Pr eferential mi gration of pr ogenitor cells into the thymus than to the LNs compared to non-progenitor cells.

(**a**) WT mice were injected intravenously with congenically marked bone marrow cells (5×10^7 cells/mouse). Twenty-four hours later, the peripheral LNs and thymus were collected and cells were stained for flow cytometric analysis. The percentage of donor-derived Lin^- cells among total donor-derived cells in each organ was calculated. Results are mean ± SEM (n=3).

Supplementary Figure 12.

a

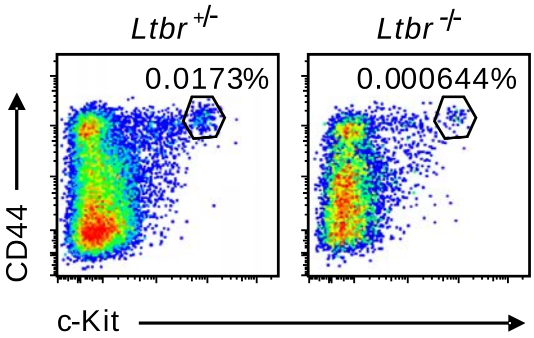

b

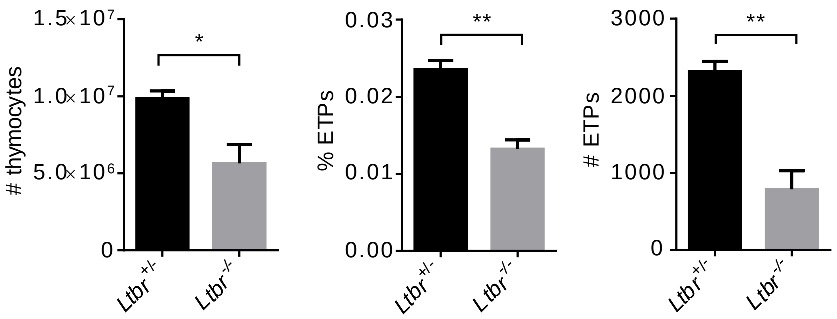

Supplementary Figur e 12. L TβR contr ols thymic ETP population and total thymic cellularity at neonatal stage.

(**a, b**) Flow cytometric analysis of ETPs ($Lin^{-}CD25^{-}CD44^{+}c\text{-}Kit^{+}$) in *Ltbr*$^{-/-}$ and control mice at neonatal stage (postnatal day 1-2). (**a**) Representative dot plots are shown. (**b**) The graphs display the statistical analysis of the total thymoctes and the frequency and number of ETPs among total thymocytes. Results are mean ± SEM (n=3 or 4).

Supplementary Table 1: Comparision of thymic ETP defect in different conditions.

**Experimental condition**	**Degree of thymic ETP integrity (%)***
WT	100
Ltbr $^{-/-}$	20.45 ± 2.61
Ltbr $^{fl/fl}$*Tek* ^{Cre}	24.31 ± 1.60
Ltbr $^{fl/fl}$K14^{Cre}	57.75 ± 9.80
Lta $^{-/-}$	72.79 ± 8.21
Light $^{-/-}$	93.71 ± 11.48
Lta $^{-/-}$*Light* $^{-/-}$	28.39 ± 6.22
Tcra $^{-/-}$	11.33 ± 2.66
T cell-*Lta* $^{-/-}$	68.19 ± 7.93
T cell-*Lta* $^{-/-}$*Light* $^{-/-}$	27.19 ± 5.94
DC deficient mice [1]	109.48 ± 5.22
B cell deficeint mice [2]	115.56 ± 10.52

*, The integrity of thymic ETP population in WT or control mice is set as 100%; the percentage of thymic ETP among total thymic cells in different experimental conditions are normalized to that in WT or control mice. n=3-9.

[1], CD11c-DTR bone marrow chimeric mice were treated with DT for 4 weeks.

[2], μMT bone marrow chimeric mice.

Supplementary Table 2: FPKM of genes differentially expressed between thymic EC subset I and TPECs. *

Gene Symble	EC I-rep1	EC I-rep2	EC II-rep1	EC II-rep2	TPEC-rep1	TPEC-rep2
1700025G04Rik	8.48704	9.02846	8.27555	7.13377	3.97857	2.98442
1810011O10Rik	182.163	196.894	173.627	147.292	110.873	106.705
2200002D01Rik	35.2296	33.5238	133.295	144.303	161.443	248.76
4930506M07Rik	5.59711	4.63864	5.51093	8.84866	14.5375	12.27
6430548M08Rik	13.0552	17.3855	10.5999	8.21288	6.69449	7.30981
9430020K01Rik	55.3194	60.4008	36.7312	38.9923	25.089	31.5479
A4galt	21.6472	19.9673	34.4885	42.2549	44.1547	54.0591
AU021092	16.8394	11.849	38.4798	51.1123	92.8207	106.74
AW112010	138.665	107.52	114.335	78.6404	70.8422	30.1397
Abca1	49.1737	64.2372	92.3077	128.45	110.439	153.051
Abca2	4.37653	2.46476	4.31207	6.43188	6.54856	9.50261
Abca7	9.86607	12.0505	5.10747	7.62677	4.9425	5.9046
Abcb1a	65.4467	62.7956	49.0796	41.2275	28.199	30.7759
Abcc9	56.455	35.0663	47.0757	27.4708	5.88189	8.01901
Abhd17c	7.85292	6.80241	8.67532	20.4325	39.2445	30.9633
Ablim3	86.9617	115.513	63.6029	62.1651	15.0294	18.6796
Acer3	2.6559	1.91439	7.50057	8.19875	10.3305	8.3303
Ackr1	2.57776	4.83629	238.775	320.197	403.512	462.066
Acss1	9.17948	10.8824	4.63635	4.39466	3.28278	1.53886
Acta2	17.6399	13.6329	44.1405	62.1762	22.2912	52.5211
Actn1	65.0945	75.7907	88.1991	120.169	118.123	168.805
Adamts12	9.0974	10.7819	12.5957	8.45652	2.52204	2.86225
Adamts18	0.792235	3.37087	4.86148	7.86028	4.02648	8.37033
Adamts7	8.43956	14.7067	6.59425	7.02148	2.84469	5.93219
Adarb1	20.8374	29.0591	10.2116	10.7821	3.86702	3.84293
Adcy6	31.5291	36.8595	32.9144	30.721	11.1622	21.0452
Adk	4.15339	2.01036	2.40021	6.01443	17.1354	7.82232
Adora2a	14.9398	13.0636	5.40532	4.26447	1.25869	2.06621
Adra2a	25.9531	17.1788	17.9988	12.6756	3.58173	4.35103
Aebp1	13.843	18.7003	38.2331	36.0259	54.0324	96.8205
Afap1l1	80.2542	92.9788	45.8006	35.9883	23.0741	29.854
Agap1	12.3208	16.2922	11.9884	10.9338	4.81959	6.09058
Ahdc1	14.3967	21.4044	11.5755	15.5166	7.6787	10.2888
Akap12	120.721	152.911	110.624	122.169	40.3625	63.0997
Akr1c14	15.5335	12.3478	7.86962	4.83717	5.49743	1.61115
Alpl	16.0633	21.2701	9.50028	15.9634	1.76955	0.518608
Amigo2	4.81763	3.85035	10.4823	15.0626	33.8106	33.5136
Angpt2	16.7149	17.176	31.2857	31.6126	47.2987	50.579
Angptl2	35.7646	22.6302	24.259	30.6554	8.49775	8.66955
Angptl4	7.7149	23.0753	7.05592	12.2345	1.72907	5.66553
Ano1	19.3701	11.2212	16.2865	18.8876	3.46735	5.75274
Anpep	10.3033	4.58423	11.7206	13.1838	16.0148	24.6823

Anxa1	67.4315	50.7408	105.216	99.8654	128.11	94.5843
Apln	20.1679	29.762	11.9653	16.7171	1.99047	2.9985
Aplnr	163.051	183.77	115.263	111.52	34.6908	46.4582
Apoe	1072.48	557.852	1371.7	1281.18	3158.71	3328.18
Apol10b	24.8241	23.0354	17.6974	14.7063	6.92186	8.32795
Aqp7	89.3193	89.8513	30.9438	19.0783	2.27545	0.345851
Arc	7.17134	11.157	4.84781	6.32618	2.66999	2.24866
Arhgap18	35.041	28.2468	28.9279	17.2006	12.5312	7.87991
Arhgap26	8.19259	14.0813	12.3936	27.3835	26.2906	31.6117
Arhgap30	4.30163	2.0456	3.2269	3.16154	10.2499	6.27812
Arhgdib	52.6099	29.8676	62.3707	39.9095	124.63	63.9491
Arhgef15	86.6344	109.418	52.7377	67.3874	36.6289	50.8279
Arhgef17	15.2395	14.2739	15.7954	13.5629	2.50189	5.51435
Arhgef28	18.5929	22.3125	11.0922	9.39044	5.69943	6.43005
Arl15	22.3085	23.9557	21.3138	12.5849	10.3921	8.64314
Arrb1	16.4447	20.2681	47.8215	72.0696	72.3597	98.0511
Arrdc3	9.6957	8.18992	17.3073	18.4741	39.4845	34.3983
Asah2	14.0197	12.5922	18.1049	22.8897	29.1729	22.0939
Asap3	1.82697	3.08661	5.60376	15.8599	10.9873	13.5828
Atoh8	17.9057	25.3121	14.9441	13.2358	4.63959	9.51594
Atp1b2	19.4712	14.7304	13.7033	17.5375	2.55305	10.486
Atp2a3	30.225	32.7795	22.4602	30.223	16.8927	15.9452
Avpi1	15.4015	16.7369	23.0838	25.8133	54.443	59.1497
Avpr1a	12.9477	8.03833	9.95213	8.05468	1.18849	3.41521
B4galt4	39.471	36.1654	27.7706	20.5518	17.7995	14.5093
B4galt6	1.16885	1.06965	3.58485	7.81127	24.3227	23.707
Bace2	11.1288	9.53085	22.6025	29.1413	47.422	50.4459
Basp1	13.6458	5.86677	4.20465	6.02171	30.2543	18.9477
Bcl11b	4.03752	1.78219	1.86951	2.74744	7.97421	6.5004
Bcl2l1	47.1868	48.9943	23.2928	21.9419	21.6978	19.2953
Bcl6b	35.0749	61.257	29.2741	25.0819	13.2726	13.0769
Bcr	24.4994	33.5647	47.2737	58.1434	60.6759	85.5631
Bend4	2.11803	3.39956	8.68376	15.1162	11.8739	12.617
Bgn	102.526	99.4149	335.871	298.713	615.458	718.98
Birc2	20.0834	15.7069	35.3513	35.986	44.7085	37.5833
Bmp4	8.47126	6.44529	24.3262	25.2562	54.8286	62.776
Bmp6	32.4884	35.3315	33.4084	48.1165	63.8294	90.9272
Bst1	1.34467	3.02849	18.6171	25.2123	66.6063	75.2644
Btbd3	32.9713	28.7643	45.7829	58.9975	57.9518	67.8078
Btg2	215.906	170.912	130.669	138.171	94.8578	91.4271
C030046E11Rik	16.7713	17.6762	12.1862	10.6913	7.31381	8.71707
C1qtnf9	223.491	214.736	216.505	185.645	89.9119	103.16
C1ra	17.2895	9.37486	41.5844	26.6576	31.6306	29.3305
Cables2	23.9032	17.6658	12.6258	11.6965	8.12612	5.41616
Cadm3	4.6585	2.56505	37.6543	58.6636	25.9052	45.4589
Cadps2	3.22868	5.95528	6.91889	12.2881	11.8741	17.8996

Calcrl	23.1215	20.65	37.2742	31.9614	68.2995	42.9407
Camk2d	11.6241	11.9495	16.9313	11.988	24.4	20.1473
Camk4	1.75522	1.29269	1.9132	3.90616	7.51772	6.15718
Car13	6.64667	2.20143	9.92504	8.19341	16.8071	11.2974
Car7	15.3561	25.5886	6.16081	4.06697	0	0
Carhsp1	37.4664	45.3321	36.1655	27.3162	22.1638	23.2089
Casd1	8.40538	6.74327	12.3737	7.9978	18.6098	13.4292
Cav1	681.25	619.95	524.82	416.722	231.266	212.124
Cav2	99.1572	74.12	80.6596	46.5428	47.715	33.1248
Ccdc3	2.16939	2.06721	4.3612	4.41677	11.2312	18.1474
Ccdc85a	30.9278	50.5485	23.726	24.5018	9.7065	14.0758
Ccdc85b	26.7906	38.2446	19.4599	22.7717	10.6583	17.1315
Ccdc88a	5.43727	4.84621	9.9988	8.54647	12.2891	10.5246
Ccdc88c	41.6913	65.2112	31.8587	35.7274	16.7172	24.1304
Ccr7	10.8702	5.98996	4.98627	6.63349	30.1821	13.8151
Ccser1	1.05165	1.44114	2.42732	6.06033	6.11865	5.15518
Cd14	4.43618	2.8541	7.82525	15.0366	20.9434	18.2949
Cd209d	0	0	0	0	10.8051	3.64549
Cd248	16.499	11.9305	15.2213	13.7905	0.746205	6.97577
Cd36	662.455	475.563	524.691	324.698	189.551	142.178
Cd55	4.4092	2.38338	13.5601	20.4234	55.574	39.0475
Cd63	97.7228	65.7398	129.351	103.662	192.792	202.243
Cd74	313.525	142.829	325.588	249.492	705.88	352.271
Cd81	552.2	569.064	357.042	316.214	246.376	271.264
Cd82	42.9271	40.5422	57.6867	60.6639	167.825	151.034
Cd9	91.6755	82.6544	139.943	107.627	161.286	183.751
Cd93	277.733	314.468	231.188	200.726	124.261	130.261
Cd97	40.6729	60.9071	27.429	24.0595	14.8776	13.4159
Cdc42ep1	61.3062	78.3103	30.435	33.4193	16.5576	22.3074
Cdc42ep2	43.6138	54.1224	32.4311	30.2137	15.0952	18.0686
Cdc42ep3	59.0123	70.3745	26.4764	26.142	23.323	18.4277
Cdh13	142.735	230.821	123.242	123.432	42.2	67.4996
Cdh4	1.13365	0.826206	2.65835	4.33692	3.6205	6.99592
Cdk14	8.5239	6.07373	6.27123	4.60249	0.372981	1.36425
Cdkn1a	216.315	250.894	193.421	157.07	106.071	149.978
Cdkn1c	27.7091	26.0462	7.42271	8.77172	10.3682	7.5496
Cdr2l	13.2345	18.5985	19.4196	30.7329	27.8089	36.3872
Ceacam1	32.7855	34.2354	22.2937	18.5696	9.56936	14.3391
Celf2	3.73673	1.60275	7.36367	2.96809	9.71755	6.32683
Celsr1	0.905793	0.7652	3.75844	6.57227	7.65206	14.1016
Cep85l	13.9962	15.0202	7.08738	11.9847	4.00823	4.83966
Cept1	10.0423	6.28298	17.3952	15.6447	25.3326	15.7738
Ces2e	14.7622	19.5635	3.49797	2.00478	2.28025	4.00343
Ces2g	6.94063	3.82134	8.77012	10.0349	16.3392	15.1121
Cfb	44.9282	49.3355	72.0283	87.9036	97.0739	136.329
Cfh	8.85559	2.99214	18.2698	6.83404	40.9386	28.3561

Chp2	5.50995	8.11108	28.8665	47.7328	47.0063	42.2712
Ckb	33.4465	26.7943	14.3968	15.9712	12.0156	10.6998
Clca1	2.32497	2.12742	1.56282	3.20467	18.7182	12.2452
Cldn15	23.3786	24.3642	5.74764	8.78017	4.28164	1.46105
Cldn5	527.39	658.471	230.766	169.023	46.6547	36.3853
Clec14a	47.4113	54.4621	68.6886	71.1136	92.3551	98.3197
Clic5	46.748	41.5855	22.0488	10.0355	1.96165	0.708388
Clstn1	90.6159	89.9746	53.6441	61.0376	46.7164	47.9943
Cmah	2.58132	1.88432	7.82353	7.93811	11.694	7.52256
Cmklr1	5.58196	4.36546	14.2976	10.0881	12.4306	23.6998
Cmpk2	16.3241	17.9209	9.48235	7.86549	5.8891	8.19989
Cnksr3	15.1465	22.8866	22.0968	41.8904	34.4907	49.6029
Col13a1	22.6315	30.7899	7.23569	6.69972	1.08714	0.446473
Col18a1	27.1527	48.1119	29.1634	37.5792	15.582	21.9184
Col4a2	921.072	1520.09	654.999	727.363	334.37	546.984
Col5a3	15.3119	13.9537	17.1821	10.8943	4.77344	6.27792
Col6a3	30.3682	27.5183	44.8588	36.9852	11.6734	18.5921
Col8a1	9.68015	10.9027	6.91225	4.50767	1.55055	1.22713
Colgalt2	16.2626	19.3933	11.583	8.90612	4.55996	5.73005
Coro2b	14.6662	20.5793	8.85983	6.75981	1.37571	1.53163
Cotl1	36.5421	32.7871	40.0644	43.4709	106.575	121.835
Cox6b2	0	0	0	0	6.33138	4.45511
Cp	15.3778	12.2857	30.0395	19.6993	55.5363	35.3151
Cpe	68.95	35.8913	85.7788	47.5947	197.692	250.989
Cpeb2	4.7005	6.6459	7.44672	5.89529	10.0089	13.5505
Cpq	10.4845	7.24448	10.2763	8.93745	24.2654	32.1982
Creb3l1	4.55722	6.24752	5.79229	5.46353	18.7395	13.197
Creb3l2	20.3813	27.1003	34.1305	35.9567	32.1726	58.2443
Csf2rb2	82.1189	89.7598	205.865	277.325	224.227	294.548
Csf3	3.88903	18.3096	61.177	92.4844	105.242	152.532
Csgalnact1	12.1915	13.2263	19.1776	26.013	26.293	25.7626
Csrp2	28.7361	18.1911	64.2402	90.6292	138.514	137.377
Ctgf	18.6324	15.1073	27.2257	38.428	81.9471	133.967
Ctla2a	156.209	155.414	207.634	231.74	597.996	530.883
Ctnnal1	3.7618	2.91273	20.9362	27.3482	51.286	55.879
Ctnnbip1	56.1523	50.8884	38.5641	30.596	20.2212	15.1369
Ctsd	266.593	196.352	335.233	321.977	501.5	455.632
Ctsh	21.6531	9.1019	16.9205	15.2912	138.464	118.686
Ctsl	179.186	115.633	314.137	298.672	609.339	580.864
Cx3cl1	51.8291	60.7237	41.95	37.1783	13.9571	16.7928
Cxcl1	232.805	205.651	323.967	194.566	88.244	91.9323
Cxcl12	671.216	651.69	366.305	254.869	66.7265	57.1551
Cxcl9	62.9174	40.2943	133.728	87.7709	121.968	77.5427
Cxx1a	33.6718	43.5069	14.7905	31.2568	14.3157	18.3382
Cyb5	74.5301	78.7743	114.036	106.556	149.669	182.828
Cygb	33.3107	25.5859	31.3981	25.9088	9.93179	10.939

Cyth3	23.7646	28.2449	29.8268	19.0879	8.62217	14.1286
Cyyr1	110.179	136.929	84.8189	95.1062	49.7694	76.9749
D8Ertd82e	22.0094	30.5328	9.47097	10.2577	3.32526	6.49023
Daam2	6.79856	5.21018	11.8219	7.33964	1.10161	3.36714
Dab2ip	67.5065	93.0461	50.9824	66.7183	34.2246	54.1113
Dach1	12.8261	21.1363	15.332	11.589	2.52992	4.1808
Daglb	3.77393	4.70562	7.45301	8.47121	9.25761	14.0006
Dcbld1	18.2897	17.8186	11.8202	9.76384	7.06378	7.82288
Dclk1	1.57848	0.1584	2.14096	3.82193	17.874	13.3662
Dcun1d3	5.3676	4.17328	8.69751	7.49394	12.535	8.96707
Ddah1	4.2759	4.29509	4.79028	5.86111	6.94238	17.1029
Ddah2	37.9612	66.8237	46.4088	26.8375	13.8844	26.2346
Dennd3	19.2848	31.4948	16.2683	22.0561	9.53169	12.9979
Des	52.5271	31.5928	44.6378	37.7196	6.75981	20.8968
Dgkh	14.8766	22.3187	12.8222	14.8558	6.37976	10.7383
Dhh	0.757363	3.41151	6.69876	5.42951	10.5795	11.128
Disp1	7.2221	10.1666	6.51076	4.99257	2.93831	4.32282
Disp2	0.318582	2.29651	3.22359	7.30427	11.4696	17.8048
Dlg4	4.2061	4.28357	7.27274	7.37855	10.8843	14.531
Dll1	18.0346	21.1692	35.4971	47.4719	30.1628	43.9361
Dll4	64.0609	97.0008	32.9209	37.6804	24.5361	29.413
Dock2	5.40392	2.37526	3.33434	2.39721	15.5356	5.94451
Dock8	6.45855	6.48651	7.83679	7.47475	13.9143	11.1355
Dok4	34.4679	45.1571	14.9536	16.5597	7.81962	14.7106
Dpysl3	16.7941	19.1426	48.4169	50.7896	48.931	56.7078
Dram1	4.87689	4.07487	11.1222	10.5826	22.2976	22.1959
Dsg2	1.63015	0.417288	4.88617	12.4155	28.8007	34.3616
Dusp1	394.994	518.021	280.941	319.004	145.681	216.464
Dusp3	59.0748	56.3867	49.1803	38.6032	32.9392	31.4111
Dusp8	15.8796	22.0816	16.4678	14.3763	5.83332	12.7838
Dysf	63.23	97.9858	39.4507	55.1223	26.0779	32.4392
E2f7	4.89225	9.79589	5.3594	4.36575	1.10976	3.4259
Edn1	8.03783	14.8703	13.2479	13.0867	31.5329	44.8025
Ednra	14.1683	9.65455	14.9898	12.3396	1.02185	5.48433
Ednrb	80.9003	64.0546	40.9846	21.0635	27.3399	24.9834
Eef1b2	81.0212	71.7589	105.084	106.367	158.336	177.923
Eepd1	35.8262	34.1819	19.6331	17.3767	8.31267	11.5373
Efhd1	7.67581	17.8621	4.73124	6.21015	0	0
Efnb1	71.597	85.3144	49.4254	49.378	28.9698	40.3365
Efnb2	57.807	70.7093	38.9342	25.8869	10.2427	15.3118
Efr3b	33.4964	35.4745	16.5246	13.8015	4.42594	3.67025
Egflam	8.95165	6.62976	9.9737	5.90802	0.7532	3.01439
Egln3	4.63849	1.49248	14.5159	12.6056	17.0795	15.4185
Ehd3	5.09738	6.00788	27.4828	50.1894	50.8701	66.6881
Ehd4	77.8276	91.1282	214.354	249.781	185.156	241.419
Eln	97.1816	156.118	226.159	273.104	243.45	428.896

Emp1	327.638	313.168	723.712	650.373	808.702	799.31
Enah	4.15006	6.00189	3.87001	4.08301	0.875913	1.70709
Endou	22.5162	19.0679	10.3053	6.97896	10.396	8.20389
Enpep	13.7022	7.94647	20.4007	5.18575	2.48563	1.50344
Enpp2	104.843	77.4403	415.951	385.5	555.718	488.066
Entpd1	74.9543	61.4373	115.976	105.697	136.647	125.933
Eogt	21.0127	22.5889	10.6891	13.0893	9.95791	10.8304
Epha4	10.8295	9.93876	4.4073	5.37916	3.34286	2.75278
Epsti1	10.5964	3.87854	9.85423	4.35219	28.9396	9.87604
Esm1	52.1949	56.5892	23.2401	9.0405	4.01948	0.422451
Etl4	37.1881	68.4271	31.3137	35.8088	12.6878	18.762
Etv6	20.2929	26.1461	39.0207	37.2832	42.182	45.8939
Extl1	1.04714	0.117935	7.39281	4.38695	11.979	12.5218
F2r	67.2736	63.2365	61.2228	68.7752	39.9818	25.3062
F8	1.61599	1.36576	17.2463	13.3689	39.6717	32.6254
F830016B08Rik	2.51532	2.8328	4.06664	8.90431	12.3127	7.52454
Fabp4	6162.26	5451.28	5738.09	4871.91	2261.6	2007.35
Fabp5	342.342	325.417	312.006	301.361	142.323	137.646
Fads3	9.48528	10.9834	8.61828	7.21805	1.14697	1.70193
Fam101b	142.264	171.795	87.0776	71.9445	27.2778	21.9631
Fam117b	24.2718	24.6558	13.4291	16.7648	12.0743	13.4039
Fam124a	1.70962	1.65037	7.68782	10.2328	6.98763	13.5237
Fam134b	1.20219	1.09986	1.47672	1.33971	9.70871	2.16666
Fam13c	22.2141	35.2181	27.5374	30.1416	12.5166	15.3921
Fam174b	1.65526	1.67768	3.05349	4.7461	23.9393	30.1635
Fam198b	34.8637	33.3815	26.383	13.489	11.9284	8.93639
Fam57b	20.1985	24.0455	10.3577	8.69887	0.275623	5.84121
Fam65b	0.85581	1.9569	2.67716	1.91684	7.54915	4.51475
Fas	29.6166	21.9278	14.7994	8.35908	8.72538	6.2548
Fat4	22.8562	40.9662	20.3019	26.0122	9.80974	17.4689
Fblim1	8.92935	6.67638	12.2912	10.6825	17.7512	23.7693
Fbln5	31.8069	47.7393	29.2425	43.3113	68.0713	85.9329
Fgl2	2.52673	1.42286	4.23166	10.5102	28.7475	29.7173
Fhod1	19.6845	26.1111	9.40027	8.31927	3.90989	6.85994
Filip1	29.1499	38.0144	25.7428	22.4396	13.0162	16.7425
Fjx1	3.31609	1.45224	5.66692	13.2069	24.5439	37.3562
Fkbp5	23.4761	20.6758	28.8637	31.8936	38.9852	44.7104
Flt1	234.202	281.395	182.749	175.425	104.93	142.294
Flt4	25.7912	30.2765	10.8067	11.7725	12.4577	11.1116
Fmnl3	68.5453	84.6578	44.3735	58.2861	32.7136	45.9069
Fn1	31.6684	57.9188	31.7885	35.2871	14.9966	14.8793
Fnbp1l	21.5388	19.9239	38.6902	33.133	39.9139	37.4941
Fndc1	1.6689	1.87972	3.56065	3.19944	9.95376	7.68312
Foxc1	8.97602	9.01311	28.219	35.7691	42.0664	63.0688
Foxf1	16.7798	13.0819	9.35889	5.69425	5.01887	5.41882
Foxp4	11.9085	17.879	24.5452	37.3714	35.5404	56.8838

Frmd6	12.6737	6.51647	16.3448	13.8705	20.6122	18.3654
Fryl	43.928	61.4302	42.2491	41.7274	22.6702	33.067
Fscn1	205.671	266.632	123.71	119.733	80.473	81.6093
Furin	57.4724	86.3084	44.8962	48.2123	32.4113	42.0791
Fut2	6.99598	24.8098	9.835	14.6085	1.06527	7.29533
Fxyd6	2.57957	2.61412	0.397522	4.83812	17.397	19.446
Fyb	4.22161	2.21273	4.50354	3.19955	16.7488	6.66812
Gaa	26.0041	19.9374	29.1995	33.9336	41.6303	59.4532
Gabarapl1	53.2609	44.6845	57.5395	67.6914	127.385	168.952
Galnt16	11.6794	9.18293	12.3466	8.86792	2.83639	3.23981
Galnt18	39.2077	49.9888	30.2748	40.1919	18.7998	24.8737
Gas1	25.6011	18.4336	22.7807	18.6654	7.40767	10.1444
Gas7	2.6244	3.84969	8.60965	13.7299	9.15522	14.9615
Gata2	20.7498	26.2518	7.65751	12.4798	6.74811	15.0523
Gata6	3.6976	2.15924	13.0426	11.6275	17.0497	29.1214
Gbp6	17.4245	18.1668	40.3378	45.0059	57.6571	59.6325
Gcnt1	1.51311	0.106416	1.14161	0.416949	9.87164	7.2939
Gem	53.6634	48.7486	83.49	89.3338	96.6269	101.801
Gfod1	17.0098	25.0922	9.24399	10.6341	6.25657	8.899
Gfpt1	4.13176	3.58565	6.95826	8.06361	12.3874	10.1607
Gja4	36.2115	48.8651	34.9476	25.0096	1.98406	5.95291
Gja5	17.4706	27.6203	21.7658	6.80606	1.60596	0.549779
Glipr2	3.61073	4.31999	7.9918	7.2192	16.5028	22.3201
Glul	190.356	199.491	136.852	112.245	108.335	81.539
Glycam1	0	0	0	1.06671	43.5616	85.3977
Gm13889	309.136	200.749	219.137	210.158	62.3782	98.0874
Gm15055	17.4986	7.87178	5.52604	0	0	0
Gm2a	11.1147	6.82811	10.2342	8.68898	25.7169	15.9459
Gm4951	15.0803	10.5671	27.8204	16.2612	32.3925	21.8331
Gm4980	3.90261	4.88343	6.44013	6.37536	12.2056	19.8285
Gm694	6.43475	10.5336	5.45636	1.29272	0	0
Gna11	45.1248	49.1612	32.29	31.0054	21.0761	25.8498
Golm1	12.7001	12.1027	21.1858	29.352	61.457	46.476
Gpc4	15.2543	13.264	15.1496	10.2225	2.21334	3.21531
Gpihbp1	630.189	709.115	241.58	180.473	78.191	56.6999
Gpr1	5.91065	4.62983	25.7409	23.5323	44.987	36.9846
Gpr126	0.389823	0.731783	3.18754	4.08212	11.9741	13.1226
Gpr182	23.3697	28.171	68.2452	67.0412	54.464	77.511
Gpr56	167.003	233.529	125.33	137.656	69.9181	105.38
Gprc5b	27.9515	32.5494	18.6091	22.4583	8.94048	11.1456
Gramd4	8.38702	9.28923	19.5898	19.0166	15.4086	19.65
Gucy1a3	14.5365	7.66802	17.2212	16.1269	2.36744	3.7879
Gucy1b3	20.5609	10.2915	19.6093	16.596	1.93342	5.92609
H2-Aa	115.631	44.8081	120.461	80.5968	305.455	122.745
H2-Ab1	172.391	79.7717	160.32	113.009	384.901	185.592
H2-Eb1	86.6866	33.6896	87.529	48.7496	233.644	102.099

Hamp	0	0	0	0	16.0218	4.1571
Hbb-bt	13.4915	15.1755	6.05387	0	0	0
Hdac5	34.5212	53.1156	23.4922	30.2317	14.4106	20.5802
Hdc	5.97052	5.46286	14.0621	26.5454	20.849	26.5009
Hecw2	8.5786	13.7285	7.42849	9.30923	2.98221	3.59008
Hes1	68.3429	57.7091	33.9889	22.4824	20.3059	16.0753
Hexa	49.8153	26.658	46.7305	44.0742	82.0762	80.1642
Hey1	31.316	30.4244	9.76419	10.8365	4.4002	1.20281
Heyl	6.81102	4.901	7.83798	4.27581	0.405723	1.76186
Hic1	15.2222	17.1673	5.47866	10.843	1.16111	6.07773
Hid1	9.85541	9.73086	13.2717	22.3294	25.8869	38.7634
Hif1a	51.5696	51.3026	107.924	94.9828	115.586	88.0714
Hip1r	27.373	34.5732	18.4517	23.9265	10.2034	15.3325
Hist1h4a	0	0	0	5.44744	14.5024	3.16968
Hs3st1	15.7668	18.6872	21.7505	28.3837	52.4149	71.7538
Hs3st3b1	3.38803	4.69051	5.51988	5.91324	10.6907	11.7645
Hspa12b	101.214	141.262	62.2565	67.9225	38.9484	57.8634
Hspa1a	49.4118	78.4023	42.2177	57.797	20.1152	34.4919
Hspa2	43.0604	43.1321	39.9708	44.533	19.2053	23.5996
Hspb8	2.8022	2.5816	16.0942	11.523	26.317	19.9526
Hsph1	9.50484	8.87356	17.6685	14.6168	23.2595	23.9002
Id1	392.626	391.314	163.186	141.49	58.4327	74.0658
Id3	343.234	363.511	263.547	226.784	148.649	205.879
Ier2	345.048	442.039	260.005	294.67	185.78	245.497
Ier5l	83.1482	103.15	62.5106	71.7345	33.4408	47.6421
Iffo2	13.2205	19.7425	10.4802	8.84234	7.854	6.55356
Igf2	10.332	18.2715	17.9575	13.4071	1.84685	3.36952
Igfbp3	419.793	463.045	111.549	75.346	23.8757	28.886
Igfbp4	142.321	170.665	293.602	366.877	392.951	554.703
Igfbp5	19.129	12.6497	33.8893	51.7574	44.9354	23.0167
Igfbp7	4404.33	6157.01	2807.95	3038.23	1119.57	1594.04
Ikzf1	5.20113	4.4633	3.799	4.27749	16.0186	9.61324
Il13ra1	21.2899	9.93546	34.4925	31.6436	37.0145	27.2062
Il1r1	28.104	20.6699	58.6532	59.1152	46.9109	49.517
Il27ra	4.00503	5.82578	10.5182	12.8822	22.2235	28.6107
Il2rg	79.7245	84.1955	138.226	166.31	195.542	217.01
Il6st	151.339	140.142	597.739	651.839	716.067	819.015
Impdh1	57.4173	64.4496	32.5815	28.0764	20.7635	26.2108
Inf2	20.671	25.1691	11.2862	10.9836	8.51927	9.67513
Insr	11.8095	16.98	21.1809	31.0123	35.6454	52.201
Iqgap1	75.7518	85.9671	114.275	133.444	149.398	182.776
Iqsec2	11.4054	17.6566	11.2908	7.23103	2.70217	6.02615
Irak2	17.2439	22.0404	9.46009	10.7143	10.7714	8.44919
Irf2bpl	60.2414	73.0394	27.6569	38.8098	24.5696	36.2498
Irf8	42.7766	26.9	60.4638	57.1572	99.0124	96.6729
Irs2	16.4505	15.1369	19.8038	15.8845	7.22671	8.00933

Isyna1	11.5317	9.59744	14.6774	18.2534	23.3779	34.3621
Itga1	70.215	73.6581	70.9458	52.6409	34.1179	30.4956
Itga2	1.122	1.72319	3.13073	4.94791	7.43825	6.09878
Itga3	12.9454	16.8616	23.6424	41.26	31.974	47.74
Itga6	124.966	125.909	88.3147	68.6232	64.6578	52.8953
Itga7	16.3087	11.7216	15.4804	13.7219	3.06873	6.30991
Itga9	7.22742	10.2435	5.81671	8.56401	16.0032	20.4919
Itgal	6.52899	3.21744	4.12882	4.67402	12.8232	9.03811
Itgb4	20.2727	34.3683	45.652	78.3814	50.9357	105.047
Itih5	63.5986	64.3516	59.2169	38.6135	9.00813	7.53547
Itpkb	159.514	198.305	104.901	115.295	61.1804	87.532
Itpr1	23.5292	32.6516	37.0252	50.6538	41.5755	61.5855
Jag2	59.1286	95.5795	40.48	41.3881	10.7975	17.5118
Jam3	88.5999	65.2926	63.2204	41.4489	35.59	37.0201
Jdp2	22.206	26.926	12.293	16.3347	8.17527	12.5398
Jup	71.6567	91.6467	28.3624	25.1728	10.9607	9.18802
Kank1	8.80762	13.1087	31.1177	42.3908	60.9133	82.801
Kank3	78.1957	102.484	46.1177	56.1273	31.132	49.3659
Kank4	6.67953	4.51386	2.32274	1.76726	0.636545	2.44455
Kazn	12.1192	17.7823	7.00245	9.06873	1.22619	2.57166
Kcna5	34.6478	34.7393	13.028	12.2517	1.25548	1.86224
Kcnb1	2.9909	3.70578	4.00943	7.66417	6.45902	10.5592
Kcne4	16.8599	7.96207	10.3158	11.1967	1.03875	2.84146
Kcnj15	3.82686	4.4685	17.3506	19.1026	29.4167	27.5368
Kcnj8	47.0671	22.7814	39.7112	17.9882	0	3.23495
Kctd12b	20.5107	17.3759	9.85037	4.32562	5.86388	1.92323
Kdr	264.55	313.268	154.248	142.371	83.582	109.01
Khnyn	17.6175	22.7301	9.41404	14.2605	8.45197	11.5844
Kifc3	32.3839	36.5323	20.7712	16.9835	4.95107	7.04134
Klf10	62.7434	58.9818	51.5076	34.1738	22.6041	18.9931
Klf11	12.463	18.1865	9.6481	9.9171	4.64953	8.0729
Klf3	39.2081	48.9613	20.604	36.3938	21.415	22.2442
Klhl29	2.27396	1.14582	4.86189	5.54068	6.92424	11.0309
Klhl3	2.7474	4.04571	5.52972	11.6556	14.8438	26.2863
Klhl36	4.86078	5.71159	8.45722	8.39204	15.4497	28.1491
Klhl6	6.51244	8.38149	8.58582	11.6974	18.3928	18.7735
Ksr1	8.02602	12.6076	14.2635	12.9237	16.0077	26.8094
Lama2	7.49126	6.59516	14.4473	10.392	1.7829	4.09531
Lama3	14.2532	25.5143	4.63988	7.38742	3.4593	2.49458
Lama4	73.6502	91.7939	59.4216	38.4686	27.6356	26.5203
Lamb1	85.4182	113.497	65.7764	49.5489	30.1213	33.0515
Laptm4b	25.4043	21.7949	47.3316	30.4278	47.5456	53.7762
Lbp	1.05867	0.993502	17.3644	23.5456	30.0735	35.6073
Lck	22.8227	10.749	18.2222	18.2278	36.8672	32.3858
Lcp1	50.7504	27.3299	52.6951	42.0659	103.658	77.007
Lef1	6.65099	4.42465	4.03433	3.44233	15.8516	12.3477

Lepr	6.52269	5.08182	16.5941	21.293	20.7735	19.843
Leprel2	10.9731	8.95476	14.4213	16.3097	29.3717	37.3951
Lgals3bp	154.11	187.865	103.344	71.0766	67.5521	40.8483
Lgmn	63.5276	32.8666	60.4576	57.0953	101.687	85.2563
Lhfp	31.7235	27.1591	40.4724	28.7613	10.591	12.5548
Lifr	28.5599	34.4844	96.2967	139.019	103.002	121.226
Limch1	28.3356	31.2939	19.2397	16.9472	11.4269	13.8937
Lims2	99.6543	133.965	55.7551	57.8708	27.7392	42.9654
Lipe	53.1513	68.97	33.6813	43.2482	18.3997	23.7971
Lipg	6.39935	3.21752	9.99954	13.4811	17.1616	13.6641
Lmo7	23.106	22.7424	22.8749	21.2632	11.5997	13.3339
Lnx2	20.853	19.9794	15.057	11.9627	4.04692	10.7294
Lox	1.62873	1.76861	6.69232	2.42923	16.8465	18.3869
Loxl4	1.40821	2.37979	6.6012	5.69356	7.15964	4.75666
Lpl	71.9837	42.9348	41.6769	21.7189	18.8823	6.88857
Lrg1	67.7594	73.8762	421.536	610.922	759.574	1191.09
Lrmp	5.049	3.93626	3.88431	7.28222	21.4254	12.2432
Lrrc16a	2.72715	2.79235	9.76063	10.6589	6.14199	11.9094
Lrrc3b	15.7564	17.1074	3.47092	2.17317	3.63555	0.826238
Ltbp2	1.47143	0.936747	12.5545	25.1047	123.48	202.419
Lurap1l	14.2489	18.2036	13.941	8.46544	4.72005	4.02355
Ly6a	771.599	749.148	438.277	402.432	270.177	233.068
Ly6c1	1299.78	1251.22	781.853	589.912	131.251	92.752
Lyve1	0	0	0	0.187943	16.3518	1.66957
Lzts2	59.3474	74.6167	31.4055	48.3095	18.0295	36.8062
Man1a	31.7774	50.4305	80.1625	90.8959	102.813	111.516
Man2b1	34.0773	27.6383	33.3888	33.5163	60.3719	54.5357
Map3k5	9.30987	12.7962	7.50244	9.654	3.49541	3.63537
Map3k6	26.7727	44.7833	29.8241	30.0625	13.894	24.1048
Map3k8	28.274	22.6287	61.5265	56.285	64.9492	63.9317
Map4k2	17.101	19.8744	6.21934	8.0993	4.99338	4.94518
Map4k3	25.5894	23.103	18.444	15.4195	8.14783	8.22537
Map6	14.1871	10.8566	7.96966	5.57848	0.275359	1.3679
Marcksl1	64.5169	74.2927	79.6134	81.8695	130.132	138.348
Marveld1	21.9493	31.1398	65.2526	79.5243	70.1705	98.3217
Mast4	27.4565	44.0987	25.1081	29.0544	12.0628	20.0601
Mb21d1	5.39299	3.15843	13.41	9.51328	14.345	9.51471
Mbp	1.20557	0.858292	1.49675	1.62389	8.81493	1.99976
Mcc	31.0971	33.8768	12.1327	12.1358	3.60551	4.26578
Mcf2l	73.3835	110.146	28.083	21.9576	3.29116	2.90252
Me1	2.68236	2.86991	5.76811	7.54222	19.1916	12.6175
Megf6	0.736284	1.03663	1.59905	2.84054	7.53817	9.38507
Mepce	22.3954	27.4014	17.626	17.53	11.4743	11.3793
Mertk	24.3156	15.9631	19.7001	13.0681	11.7113	7.18503
Met	1.20629	2.07379	4.9641	6.01851	11.5193	9.52426
Mgll	194.135	230.436	117.256	126.137	49.1527	53.2615

Mgp	260.902	216.286	588.884	290.06	499.529	529.112
Mical2	4.48199	6.32847	8.8069	5.35794	11.3633	12.4409
Micall2	5.73408	12.1818	6.80506	3.59234	1.49169	0.383134
Mlec	14.1733	15.8835	7.86342	5.57421	7.22563	6.42423
Mllt4	89.6402	97.5618	61.8845	64.1735	32.9073	40.2015
Mme	0.222549	0.0835326	1.6614	0.955431	9.01843	6.72797
Mmrn1	0	0	0	0.101874	15.5852	0.905543
Mnd1	7.34517	2.75537	9.52246	2.70564	0	0
Mpeg1	28.4377	4.91032	17.182	5.68041	57.8023	12.0436
Mpzl1	71.5772	59.1239	54.5554	38.0835	17.4504	20.5055
Mr1	8.1728	2.60104	9.83566	8.42562	19.0861	11.4873
Mras	3.63657	3.61387	7.71584	8.13656	11.1657	15.6205
Mt1	188.308	196.744	161.341	114.461	51.6661	79.1315
Mtss1l	17.1763	23.7467	11.1544	10.7201	5.32651	4.63255
Mxd4	143.343	158.997	85.8799	100.249	38.7461	64.5317
Myadm	186.26	232.971	121.839	100.007	51.7906	55.392
Myh10	10.9106	18.0688	8.36147	10.2995	5.55472	6.30257
Myo10	79.0824	123.537	52.5351	62.7026	33.9223	52.172
Myo1b	46.344	40.7097	42.8762	41.4898	14.2979	20.3522
Myo1e	30.3169	43.9711	26.4998	29.9934	18.1486	18.4586
Myof	7.30808	7.42834	17.6696	28.8161	26.3114	29.8862
Myrip	2.7845	3.23732	11.5755	15.0513	15.1513	20.7803
N4bp2	1.77771	2.50282	2.61259	4.00091	6.13909	6.46316
N4bp3	29.7457	39.1459	25.527	19.6446	11.1456	15.9257
Naaa	6.82563	2.91542	10.4981	9.96944	15.592	18.613
Naglu	9.54914	11.1594	20.781	20.8643	22.9691	30.5342
Nav1	23.655	41.4718	23.1591	25.3885	11.2105	20.6745
Nav2	7.14525	17.9634	4.62697	7.46917	2.74902	4.25181
Nbl1	40.7538	20.7237	39.7132	25.8177	7.16916	18.7904
Nck2	32.0149	35.1573	13.9543	17.7403	13.6826	18.2584
Nckap5l	17.6971	25.9811	14.9928	13.0062	9.6908	11.6519
Neat1	46.5532	139.292	44.4303	72.8979	26.6083	43.4593
Nes	44.7959	72.0225	25.0157	26.5945	4.04973	5.35442
Net1	8.66879	6.03117	21.236	22.1229	31.1139	29.5581
Nfatc4	5.83834	4.38732	6.9844	18.192	9.66158	21.9613
Nid2	43.9152	67.1966	24.1564	21.5058	9.75359	9.86212
Nmt2	3.79295	3.94563	9.23469	5.70004	13.1579	5.802
Nod2	12.9767	25.7859	4.6931	8.48137	4.37281	2.90757
Nos2	14.5995	14.9813	5.3118	2.50384	1.23725	0.741986
Notch2	4.01319	3.44622	4.50505	6.08735	8.51043	9.39087
Notch3	70.2989	71.2573	68.3442	73.4041	12.3905	28.9424
Notch4	88.4639	138.617	43.6397	45.9711	17.0889	21.3864
Nov	7.20161	8.32326	26.2369	34.2794	34.4832	32.6723
Nova2	13.1809	24.8945	8.90975	13.5891	5.86943	12.7776
Npc2	36.9568	26.8426	30.6446	32.1865	73.958	47.3695
Nr2f2	18.2931	12.5383	29.7685	29.8148	40.8885	36.7543

Nr4a1	179.891	210.092	140.819	158.638	78.491	92.8789
Nrp1	231.982	204.334	153.538	120.635	73.4802	68.4639
Ntn4	3.34015	7.52282	14.8414	10.4376	11.9626	19.4496
Nuak1	14.5427	13.41	26.6133	36.6572	25.5271	34.0155
Nxpe4	23.7217	20.5026	19.9148	17.6407	11.8419	9.32524
Odc1	106.969	97.6297	248.064	213.581	255.592	245.714
Osbpl5	4.33393	8.22079	6.65328	8.42494	11.4187	17.1027
P2ry2	32.8058	43.543	18.6771	20.8329	2.51548	6.60124
Palld	1.5842	1.37244	1.49693	5.37446	4.18382	9.67382
Palm	39.0866	54.6727	16.2086	17.5331	21.2972	21.1742
Pam	37.0684	35.3622	81.551	76.9884	135.145	152.744
Parp8	5.53737	4.47725	8.0708	9.70687	13.8178	10.8521
Pcdh1	89.4611	123.575	58.8051	66.5282	29.9275	41.3458
Pcdh12	32.6268	34.1782	18.1953	20.6238	13.1507	10.7369
Pcdh19	18.5926	15.5363	13.9995	12.4818	2.46826	4.84328
Pcdh7	1.94071	1.02354	16.8936	31.8756	51.3538	74.2388
Pcp4l1	54.3862	78.7304	27.1993	21.7632	5.57945	8.18902
Pde7b	5.11398	8.58075	6.0868	11.2762	23.1323	25.2676
Pdgfb	96.1661	133.565	52.857	55.5783	32.9684	34.7307
Pdgfrb	50.2478	43.749	50.167	42.1262	8.42896	14.6465
Pdia5	7.40144	11.4952	77.0828	82.5014	96.3111	118.19
Pdlim1	244.378	284.008	379.113	395.839	435.275	541.471
Peak1	25.1874	36.0302	23.5724	30.9844	13.3981	18.3672
Per1	81.1295	116.211	50.3501	70.2954	32.7054	56.7421
Per2	4.86935	6.54859	2.67477	5.2074	1.35006	2.28012
Pgm5	4.73738	6.04743	11.1333	15.1428	31.802	35.098
Phldb1	31.3063	61.399	29.1429	28.5872	14.6908	16.2622
Phlpp1	17.0693	26.9626	16.5976	13.844	7.634	7.34725
Pi16	9.23063	12.8802	15.4382	20.141	2.87568	2.55506
Piezo2	4.31913	8.32742	3.15965	3.30223	1.27786	0.648702
Pik3c2b	15.2617	23.5169	8.44686	9.17025	4.08719	5.19621
Pik3cb	8.27624	9.02396	5.40281	6.40605	3.14637	3.79767
Pik3r3	19.1909	16.8114	15.439	11.9413	11.3379	7.27446
Pik3r6	7.72556	12.3214	4.14742	8.48519	3.09097	3.30746
Pitpnc1	24.379	30.3097	17.0553	16.4468	10.9443	7.11857
Pkig	93.9044	93.0973	64.1335	56.1522	40.2714	52.0427
Pkm	165.8	202.029	225.883	251.989	272.797	408.737
Plcb1	24.0844	33.9806	33.266	28.7366	12.0116	15.3007
Plekha3	15.3393	16.4524	26.2619	23.8416	31.5737	30.9198
Plekha7	5.38193	5.83701	16.8238	24.7224	19.3803	36.3419
Plekhg5	44.5383	63.9273	24.611	30.9023	18.4496	29.2389
Plin2	47.9467	46.3154	34.8676	31.4145	17.8053	19.7794
Plscr1	28.3339	29.5251	51.746	61.5735	72.136	64.4036
Pltp	137.823	131.579	91.4131	100.711	37.7899	41.8069
Plxna2	30.4239	50.6157	29.9355	35.6144	18.5631	26.278
Plxna4	5.58697	11.9904	8.65981	15.8493	14.2536	20.2958

Plxnb2	20.2665	21.3128	37.6985	58.9362	53.8801	78.637
Podxl	342.71	375.561	162.065	154.515	44.2878	59.8202
Ppap2a	99.3182	129.183	60.6279	39.2015	28.8541	23.6544
Ppap2b	262.74	311.801	163.953	156.152	105.192	138.906
Pparg	37.9142	46.5985	30.9238	28.4318	11.376	10.433
Ppp1r10	38.804	57.3539	27.5092	29.5308	18.6406	23.2606
Ppp1r13l	6.85729	11.6628	4.5147	6.17251	1.80248	3.83973
Ppp1r14a	20.0916	24.0072	61.6091	111.376	141.921	196.495
Ppt1	20.0382	16.7228	25.5873	19.337	45.1031	28.0425
Prcp	31.7503	31.7466	57.2212	64.2966	77.5142	85.4809
Prdm16	5.01988	6.51937	2.13427	2.82849	1.25959	0.908178
Prex1	18.6375	19.0969	21.9268	25.8939	35.8727	39.2757
Prickle2	5.13697	6.20918	3.9233	3.56133	1.56107	1.75706
Prkag2	4.98487	3.76023	8.39953	13.7552	24.22	24.8628
Prkcg	21.6553	44.5299	9.19626	12.0826	2.10581	3.36209
Procr	35.6133	28.3872	51.5605	44.1706	99.1676	131.385
Prom1	9.08588	8.90306	5.495	5.55865	1.62613	0.639269
Prr7	34.3833	41.104	28.4781	31.2996	7.64617	23.9274
Prrg3	6.03945	8.50293	3.96908	3.64573	1.02736	1.55079
Prrx1	12.6719	8.59708	21.2254	10.7846	5.90674	4.00976
Psen2	19.4175	23.1512	57.689	52.6676	59.2916	87.2431
Psme2	147.936	147.843	111.33	102.505	64.4674	92.4561
Ptgfrn	17.6019	19.8253	14.8459	12.9354	7.65051	7.69378
Ptgs1	14.7677	13.3395	30.7326	40.882	67.1661	68.1273
Ptgs2	2.80503	1.05307	4.46446	3.09251	11.5874	10.9978
Ptk2b	4.86377	2.24093	6.98187	10.4896	17.063	21.2153
Ptk7	7.88331	8.99256	16.7511	17.8279	16.1854	23.9743
Ptn	12.8491	8.37663	52.0627	26.607	23.9187	25.3861
Ptp4a3	46.9739	47.3554	24.6369	26.1268	13.0948	16.1505
Ptpn14	14.6805	25.3723	25.2887	26.2393	40.4813	58.3486
Ptprc	13.6507	5.20976	9.05457	7.5144	38.0415	12.1377
Ptprg	46.5112	62.5482	36.7882	33.6405	16.1987	15.2777
Pxdn	81.0452	115.813	61.9792	71.7353	40.5113	48.4045
Rab11fip5	21.6258	30.9632	14.0442	19.351	10.9036	15.8959
Rab3il1	6.77421	8.71828	17.8654	23.482	23.0179	27.8686
Rab8b	22.9602	16.023	34.1201	38.5135	53.2868	38.4835
Rai14	23.9233	35.0087	36.2413	54.5661	54.4325	55.9791
Ralgapa2	2.65473	3.52942	5.06812	8.39423	7.20903	8.3221
Ralgds	46.6715	63.8034	76.4549	89.3957	83.3383	119.711
Rapgef1	77.9965	131.553	55.1264	72.071	32.4303	46.6266
Rapgef2	38.2965	56.5187	31.5849	31.9628	17.9918	18.6161
Rapgef3	30.1609	56.5561	19.2677	31.9151	10.512	23.1997
Rapgef4	14.1212	23.2074	9.31658	8.1679	3.15101	1.15679
Rarg	23.2112	27.0029	11.339	12.4825	8.1425	12.9088
Rasal1	2.02625	2.87558	5.97027	16.5347	21.0305	31.1085
Rasal3	2.82948	1.195	2.35308	1.55983	9.10637	4.73739

Rasd1	33.5804	48.659	28.8255	42.2092	3.77393	11.1463
Rasgef1b	7.92162	8.26244	21.6365	18.6098	35.918	23.8651
Rasgrf2	12.6927	18.5591	15.8866	9.43405	3.49318	2.05523
Rasgrp2	16.2673	22.7241	10.4572	7.49514	2.95126	3.4285
Rasgrp3	41.7713	47.4503	28.3873	25.4441	13.358	11.4899
Rassf1	75.6214	68.1984	54.9995	49.134	29.2629	35.422
Rbp1	5.4084	9.70701	5.97632	9.50587	23.2352	34.1095
Rbp7	171.606	223.797	48.0641	20.9861	0	0
Rcan1	36.2758	47.1321	368.743	389.26	421.534	556.283
Rcsd1	23.4192	18.6529	13.9307	17.2419	8.64662	7.97214
Rdh10	7.63872	7.05804	22.299	12.0976	24.1365	21.1032
Ret	3.35839	4.79873	23.5836	31.9716	10.6664	13.8431
Rfk	17.6181	14.9815	38.7056	32.3181	76.2232	75.9039
Rftn1	34.3616	49.3476	22.0452	25.5514	19.8584	17.1422
Rgcc	433.739	404.298	129.264	77.2135	19.1997	15.7934
Rgma	6.53769	5.59041	5.39093	5.20598	1.03913	1.89845
Rgs1	9.09804	4.91649	12.9353	3.61529	43.9702	6.76397
Rgs12	12.6305	20.6767	10.5747	10.3758	4.11316	6.02467
Rgs16	227.356	156.69	197.198	180.597	77.3769	107.302
Rgs2	11.6788	5.42541	8.97199	7.72689	25.4916	27.8938
Rgs5	445.873	206.052	425.922	230.328	64.0853	70.4643
Rhbdf2	21.8945	41.8121	15.8945	17.3178	14.2976	13.8778
Rin3	45.8539	63.0523	32.791	40.384	28.1547	27.6231
Rnd1	194.436	194.957	297.319	338.529	380.854	472.976
Rnf144a	25.1275	28.9241	27.2716	21.8288	14.4173	14.4809
Rnf19b	47.3207	45.1674	188.538	263.848	314.44	418.421
Rps3	232.949	225.659	420.3	400.87	666.221	701.985
Rps6ka2	10.3097	14.2711	6.0352	8.67555	2.81182	4.62931
Rrad	34.9626	38.0194	32.7313	36.6018	15.88	16.6705
Rsad2	41.0474	38.7606	25.8027	15.7578	20.1149	11.7666
Rtp3	13.6451	16.5954	7.55467	9.22999	2.34553	2.31688
Rusc2	29.4059	39.4058	16.9306	25.3032	10.6767	20.7219
S100a6	86.8503	131.777	87.2032	58.1633	35.8805	62.1497
S1pr3	36.0634	18.2078	33.1723	22.3572	5.88074	8.4392
Satb1	15.1504	7.59553	13.2555	10.285	27.2535	16.156
Scn1b	20.626	41.5196	49.1946	70.6992	66.4505	111.323
Sdc3	87.1535	80.8495	49.6581	38.9043	48.8477	45.7072
Sec14l1	75.617	96.286	58.1768	74.8549	43.1576	46.4103
Sele	16.3313	16.0079	310.447	344.839	354.997	439.459
Selp	10.1289	9.41112	468.974	698.706	692.859	1020.56
Sema3g	98.6581	152.697	49.106	39.0245	1.85019	1.49822
Sema4c	98.9801	119.027	66.9901	60.6412	27.9558	31.4089
Sema7a	266.802	297.623	109.565	86.5942	33.0062	28.0924
septin 4	150.14	161.183	121.505	85.6036	67.8158	71.6645
Serpina1e	0	0	1.083	1.54753	18.6489	13.0311
Serpina3f	6.99379	9.28069	27.3589	31.511	46.2695	44.3888

Serpina3g	12.9705	12.5555	42.747	50.4543	87.4475	96.9316
Serpinb1a	20.5522	12.1094	32.7601	29.2634	40.1041	39.78
Serpinb6b	3.21757	1.39347	2.28786	3.27662	31.9561	11.8743
Serpinb9	22.3134	13.1426	18.7093	15.2708	40.3641	37.1991
Serpine1	14.967	20.6196	34.6054	33.0081	39.3083	42.4309
Serpinh1	385.389	467.156	299.333	313.415	205.964	247.179
Sertad1	131.201	117.687	76.0605	72.6574	58.7814	66.3862
Sesn3	5.86898	4.72067	10.9683	10.8664	13.5198	16.6281
Setbp1	1.30138	1.9387	4.88858	5.92248	6.05451	9.30391
Sez6l2	13.4855	23.4612	8.56797	4.31615	1.15267	0.781388
Sgk1	288.21	294.759	218.926	166.523	131.284	118.197
Sh2b2	8.67319	8.19775	24.9922	31.4256	23.946	42.6416
Sh2b3	45.124	56.2817	30.688	33.706	16.2232	24.4794
Sh2d3c	40.6974	60.068	29.6087	27.6206	9.41784	18.8631
Sh3bgrl	54.9214	36.9705	109.274	70.6872	106.691	61.1288
Sh3rf1	14.2308	16.4914	8.45367	6.89169	4.58516	5.64493
Sh3tc2	5.47653	6.16811	0.869275	3.74744	1.88987	1.1107
Shb	34.3405	36.8574	50.3062	66.139	65.5581	93.6693
Shroom3	1.72775	1.80703	3.21632	5.87519	8.11023	11.0765
Sipa1	49.1881	71.7355	31.5108	28.5946	17.1179	30.2866
Sipa1l2	24.7848	37.6452	15.1799	13.6235	2.75313	5.60281
Sirpa	22.7576	7.25459	42.9285	43.1697	90.0762	73.9232
Slc12a2	27.2228	23.7265	17.8872	17.6472	13.2421	13.9176
Slc16a13	14.8022	37.3074	8.67095	11.8548	4.03825	3.97044
Slc16a2	10.9789	8.98223	6.99422	5.02433	4.29504	3.55327
Slc16a9	1.82847	0.411856	4.49212	4.30085	9.06764	7.52632
Slc1a5	28.861	25.8213	51.2613	67.0137	58.7693	59.6954
Slc20a1	31.9935	38.9226	24.9813	30.0385	19.2737	18.2254
Slc26a2	2.68559	2.39452	9.79115	9.25293	12.163	9.87078
Slc28a2	18.2588	17.199	10.1919	6.22221	3.16522	1.95229
Slc2a1	16.6023	28.8238	53.1813	78.0063	73.8087	113.174
Slc30a1	30.2204	28.8996	16.6172	20.653	15.3674	9.74243
Slc37a2	2.74583	0.883507	2.25713	0.972937	8.6477	2.97991
Slc39a14	14.0428	15.7187	13.1658	12.0406	4.61763	3.99392
Slc6a6	84.2788	119.43	41.4618	43.3488	16.4742	11.8429
Slc7a2	7.46182	3.37412	5.76025	3.54201	3.18965	0.96132
Slc9a3r2	259.835	317.044	133.699	134.707	53.3753	54.955
Slco2a1	9.73062	14.5721	16.5765	28.0579	46.1801	50.0984
Slco2b1	47.3078	50.0679	86.6184	104.918	129.052	158.144
Slco3a1	15.3918	23.4276	30.174	40.5438	31.4979	44.415
Smap2	49.7275	43.1968	65.6991	75.5665	88.112	86.6042
Smtn	52.7146	86.2408	41.5672	52.0798	26.9368	39.8453
Snai1	38.8342	51.0676	32.4685	24.2065	11.0694	26.1126
Sncg	62.9473	61.204	162.726	177.903	180.538	163.687
Snx5	18.4606	10.1802	20.7692	15.0025	51.5975	17.3755
Socs2	42.1028	33.74	80.1533	64.4146	109.6	99.5368

Sod2	23.4129	17.8339	44.9705	35.665	42.3833	43.6774
Sod3	6.67444	5.76212	23.292	19.8787	61.8837	100.004
Soga1	19.4487	34.5413	18.8183	20.3267	10.0924	15.7152
Sorbs1	18.9747	20.1738	13.4574	12.3093	6.16545	10.1733
Sorbs3	89.2336	106.963	44.5678	57.9002	31.904	45.2293
Sox13	35.5674	54.0563	25.3231	27.1391	16.0215	16.8859
Sox17	52.3959	54.2888	32.5909	37.1201	13.1873	17.0355
Sox18	122.758	187.769	73.2334	78.9089	56.1252	82.4643
Sparc	1680	1927.12	1163.34	1008.95	558.428	690.118
Spata13	25.6379	43.3158	20.2495	19.8678	10.2348	14.6004
Spats2	10.1623	10.4483	16.3254	16.5711	20.2713	26.178
Spint2	60.7175	33.218	104.429	151.987	168.369	244.991
Spns2	66.0476	77.941	96.8241	130.141	114.579	146.547
Spred2	42.3617	54.2751	30.1914	26.4035	12.524	18.9485
Spry1	87.4238	118.423	68.8739	62.6315	30.0229	52.8241
Spry4	39.2054	54.6698	35.0896	26.3444	10.2755	17.2942
Spsb1	9.19542	6.79601	8.82972	17.4276	15.834	20.2735
Ssu2	11.0057	15.6944	2.83493	2.83461	0	0
St3gal4	42.8667	30.3269	63.2362	57.4366	84.9554	81.3193
St5	18.6716	20.9529	12.4109	11.3702	8.29998	7.20726
St6gal1	5.6146	6.64259	10.7555	10.6967	26.2443	26.3887
St6galnac2	34.935	43.8778	31.0313	28.9024	12.8437	10.3072
Stab1	28.2681	42.7886	45.3388	78.6475	111.069	206.964
Stard8	24.69	23.6419	9.36978	13.0875	4.3038	9.19246
Steap3	6.10004	7.412	11.35	18.4106	12.871	18.8733
Steap4	62.2027	30.0002	55.7051	27.5278	4.79019	4.78333
Stk10	23.7116	26.9905	20.2498	15.5724	11.5374	14.5901
Stk32c	5.58802	7.45764	16.8803	15.9793	18.909	24.7288
Sulf1	5.29777	5.67929	12.9479	11.5462	39.3256	56.3713
Sulf2	6.62046	3.53426	6.13491	8.66643	14.9316	18.7656
Susd2	9.39463	6.68813	21.7826	19.0907	17.6513	25.7523
Sybu	9.6178	11.5943	4.6498	3.83743	0.431271	1.13668
Syne1	5.07337	12.109	3.88869	4.3273	0.431401	0.947521
Syne2	15.6581	29.2958	17.0726	21.9947	5.1049	8.90118
Syngr2	65.8011	69.1933	96.7542	117.483	157.404	180.731
Syt15	11.7294	12.8874	33.6295	38.2908	24.1263	35.8012
Tagln	43.2809	43.057	171.488	230.943	101.753	156.095
Tanc1	36.3201	53.2757	37.1554	38.6513	17.9646	27.7234
Tank	6.93374	12.2173	20.5287	12.963	26.5712	20.287
Tbc1d30	1.0438	0.92372	3.31578	4.84902	3.72896	5.84766
Tbl1xr1	4.5984	4.3065	12.1948	7.68811	16.9728	10.9434
Tbx3	17.5217	22.061	12.9154	14.6072	1.91971	4.2193
Tcf15	65.9902	62.8434	28.0422	33.0264	10.5945	11.5688
Tes	11.1625	12.3643	16.3233	23.0074	47.9707	44.097
Tet3	19.9279	28.9519	19.1625	17.1999	11.1958	13.9163
Tfpi2	6.28389	5.30627	38.7931	19.0702	33.8565	31.3621

Tgfbi	14.2353	5.77867	87.7878	105.874	224.698	249.254
Thbd	196.8	226.238	122.18	111.253	73.665	97.6398
Thbs1	67.4272	78.4979	188.855	297.63	244.429	378.786
Thrsp	37.8487	44.8151	18.2934	15.2008	1.41814	5.76443
Thsd7a	2.72996	3.11768	10.8629	14.7934	8.06649	12.6052
Timeless	19.3928	22.5812	11.0812	11.299	7.11131	5.03673
Timp2	41.5681	35.6176	92.7004	95.6255	137.429	153.565
Tinagl1	355.536	422.222	290.896	300.804	120.843	209.252
Tiparp	12.3347	8.63883	22.7485	18.627	26.9756	19.6086
Tll1	3.89995	4.09965	14.8903	17.7704	17.4669	18.0963
Tlr2	9.88603	8.1761	21.8399	17.7153	31.6222	24.2158
Tmcc2	21.3288	37.1103	22.3597	21.8274	11.6945	17.1474
Tmem158	6.69798	9.05014	7.02074	9.7548	26.5326	53.5049
Tmem173	54.8868	49.6524	86.3393	93.6054	95.9253	125.294
Tmem2	35.2171	47.986	57.7935	69.6838	66.7932	87.2564
Tmem44	20.588	24.7868	14.786	18.5607	7.97583	12.3269
Tmem98	16.8863	14.7435	24.4857	25.4839	47.0697	48.5517
Tmod2	1.90139	2.04642	5.56783	6.00756	6.99896	6.66547
Tmsb10	323.039	347.958	348.112	393.842	715.364	675.502
Tnfrsf10b	62.9009	73.7845	43.766	38.2456	26.1964	33.8539
Tnfrsf11a	3.31399	5.26384	13.817	17.8919	16.5181	20.0758
Tnfrsf21	37.212	25.6039	38.0287	19.393	11.4675	9.96771
Tnfrsf9	6.05588	2.71845	10.2462	10.0626	44.692	58.1671
Tnks1bp1	52.0694	64.9688	47.7966	47.628	22.5856	36.4223
Tox	3.09289	1.2191	3.08958	3.24012	10.8479	9.24274
Tpm1	86.3512	81.4207	119.84	142.122	144.773	179.545
Tpst1	44.9439	41.3851	33.7445	16.3628	10.7926	7.58861
Traf1	10.6201	8.48028	19.0113	21.0839	25.7301	34.801
Trak1	30.3493	47.9732	26.7005	27.9613	14.8773	16.9764
Trf	27.3289	13.8706	23.3894	9.7643	65.9049	29.5968
Tril	17.3164	26.7503	12.1831	6.65486	2.0434	1.71322
Trp53i11	124.787	162.106	68.8968	78.2698	63.9374	78.5107
Trp53inp2	26.4543	30.5361	32.1824	20.5737	15.0705	14.3093
Tsc22d1	207.118	158.173	188.816	184.539	80.0225	87.3891
Tsc22d3	44.1734	46.8426	32.834	26.8208	17.8452	16.1508
Tsku	4.74607	6.19848	14.4723	13.9847	15.0104	20.1329
Tspan13	152.74	110.009	91.3862	67.8195	68.6951	38.2645
Tspan2	11.202	9.77489	12.545	4.11666	5.4834	3.46223
Tspan5	20.1427	17.2466	23.3124	19.168	39.8812	41.2341
Tssc4	20.661	34.6469	14.7281	15.9855	5.44556	15.0598
Tuba1a	128.783	139.015	111.632	93.2169	64.9025	63.8901
Txnip	157.747	147.099	108.88	108.912	68.4146	70.649
Uaca	165.843	203.597	143.824	128.87	49.3341	63.3727
Ubd	35.2418	20.7762	82.5572	110.007	286.101	252.981
Ugcg	45.5459	31.8794	67.2424	57.6656	86.4734	69.0705
Unc119b	16.0732	22.5273	8.0433	7.0887	4.25772	4.90089

Unc5b	26.3142	43.8059	13.8571	9.80176	4.75179	1.77191
Upp1	71.5453	85.6668	100.801	116.865	123.01	177.508
Vamp5	33.4568	31.8082	48.2237	61.7725	65.9495	68.4549
Vash1	37.8261	60.7865	33.0374	29.1796	15.4417	19.4486
Vav1	3.82185	2.55924	4.19784	1.36648	12.9971	5.28547
Vav2	6.99502	6.89324	11.1252	16.2517	18.4062	17.6279
Vcam1	71.6624	40.1373	164.393	159.445	364.08	250.89
Vldlr	0.357248	0.172419	1.72094	2.47538	10.4248	10.2945
Vps13d	15.2325	23.4631	15.1503	18.6307	8.14319	12.9096
Vstm4	12.4191	7.99195	12.8914	6.93666	1.84744	0.790103
Vtn	39.343	24.8436	26.2236	25.8098	5.72273	7.54693
Wbp1l	31.2728	38.4489	48.0404	61.8464	74.0152	113.791
Wdfy4	2.76442	1.84879	2.25031	1.04737	6.4148	5.12529
Wipf3	0.210035	0.591448	0.483568	1.73698	8.48889	12.0396
Xbp1	64.7856	59.6958	95.9906	82.0932	118.709	116.827
Xdh	71.9495	69.3246	45.2964	42.7812	21.368	18.7472
Xylt2	15.6409	19.3337	8.98298	10.5157	8.18684	8.22489
Ypel2	6.27446	11.0911	15.6413	20.3666	13.9514	21.3573
Zbtb16	10.5895	22.8208	10.232	10.3868	2.38352	6.29413
Zbtb46	28.2379	33.9485	17.7589	22.9611	9.63224	18.2674
Zdbf2	0.5626	0.372739	1.21677	2.29169	4.37284	5.18453
Zfp366	30.3041	50.5591	22.9197	21.3949	12.6262	13.1958
Zfp423	11.9262	27.355	28.8455	45.2004	34.3435	63.3211
Zfp521	6.47084	9.14911	18.7883	29.0626	24.6878	38.3358
Zfp57	2.98292	0.740869	5.08082	8.94675	4.89114	11.7781
Zfp703	23.9545	32.1735	40.8329	54.2579	51.6	74.6459
Zfp827	4.97878	6.23093	1.61106	2.9878	2.44955	1.89853
Zfpm2	1.37327	3.86677	4.60823	9.55641	5.64335	11.1906

*, EC subset I: Ly6C^{+}Selp^{-}; EC subset II: Ly6C^{+}Selp^{+}; TPEC: Ly6C^{-}Selp^{+}

Supplementary Table 3: Shared and unshared signature genes of thymic TPECs and LN HECs.

Signature genes shared between TPECs and HECs*	Unshared TPEC signature genes	Unshared HEC signature genes*
1700025G04Rik	2200002D01Rik	0610009B22Rik
1810011O10Rik	4930506M07Rik	1110008F13Rik
9430020K01Rik	6430548M08Rik	1110008L16Rik
A4galt	Abca1	1110032A03Rik
AU021092	Abcb1a	1110036O03Rik
AW112010	Abhd17c	1110037F02Rik
Abca2	Actn1	1190002H23Rik
Abca7	Adamts12	1200009I06Rik
Abcc9	Adamts18	1200009O22Rik
Ablim3	Adamts7	1600012F09Rik
Acer3	Adcy6	1700029G01Rik
Darc	Adk	1700029I01Rik
Acss1	Adra2a	1810030N24Rik
Acta2	Ahdc1	2010110P09Rik
Adarb1	Amigo2	2610019F03Rik
Adora2a	Angpt2	2610024G14Rik
Aebp1	Angptl2	2610030H06Rik
Afap1l1	Angptl4	2610305D13Rik
Agap1	Ano1	4922501C03Rik
Akap12	Anpep	4930420K17Rik
Akr1c14	Anxa1	4931406C07Rik
Alpl	Aplnr	4931406P16Rik
Apln	Apol10b	4933409K07Rik
Apoe	Arc	5730471H19Rik
Aqp7	Arhgap26	5730494M16Rik
Arhgap18	Arhgap30	6230427J02Rik
Arhgdib	Arhgef28	6330578E17Rik
Arhgef15	Arrdc3	6430527G18Rik
Arhgef17	Atoh8	8430408G22Rik
Arl15	Avpi1	9030617O03Rik
Arrb1	Avpr1a	9830001H06Rik
Asah2	B4galt4	9930014A18Rik
Asap3	Basp1	A130022J15Rik
Atp1b2	Bcl11b	A2ld1
Atp2a3	Bcl2l1	A630007B06Rik
B4galt6	Bcr	A930039A15Rik
Bace2	Bend4	Abca3
Bcl6b	Bmp4	Abcb7

Bgn	Btbd3	Abcd3
Birc2	C030046E11Rik	Abce1
Bmp6	Cadm3	Abcg2
Bst1	Calcrl	Abhd2
Btg2	Camk2d	Acacb
C1qtnf9	Carhsp1	Acad11
C1ra	Casd1	Acer2
Cables2	Cav1	Acsl4
Cadps2	Cav2	Acvr2a
Camk4	Ccdc3	Acy3
Car13	Ccdc85a	Ada
Car7	Ccdc85b	Adam19
Ccdc88a	Ccr7	Adamts1
Ccdc88c	Ccser1	Adcy5
Cd36	Cd14	Adi1
Cd55	Cd209d	Adrb2
Cd63	Cd248	Adss
Cd74	Cd9	Agpat5
Cd81	Cd93	Agphd1
Cd82	Cdk14	Agrn
Cd97	Cdkn1c	Ahr
Cdc42ep1	Celf2	AI597468
Cdc42ep2	Cep85l	AI987944
Cdc42ep3	Ces2e	Aim2
Cdh13	Cfh	Ak2
Cdh4	Chp2	Akap13
Cdkn1a	Ckb	Aldh18a1
Cdr2l	Clca1	Aldh2
Ceacam1	Clec14a	Aldh3b1
Celsr1	Cmklr1	Aldh9a1
Cept1	Cnksr3	Alg2
Ces2g	Col18a1	Alg3
Cfb	Col5a3	Alg8
Cldn15	Col6a3	Alox12
Cldn5	Colgalt2	Amigo3
Clic5	Cox6b2	Ankrd29
Clstn1	Cp	Ankrd50
Cmah	Cpq	Antxr1
Cmpk2	Creb3l1	Anxa6
Col13a1	Creb3l2	Aoc3
Col4a2	Csgalnact1	Aox1
Col8a1	Csrp2	Ap1s2
Coro2b	Ctgf	Ap3m1
Cotl1	Ctla2a	Apbb2
Cpe	Ctnnal1	Apc
Cpeb2	Ctsd	Apex1

Csf2rb2	Ctsh	Apobec1
Csf3	Cxcl1	Apold1
Ctnnbip1	Cxcl9	Aqp1
Ctsl	Cxx1a	Aqp11
Cx3cl1	Cyb5	Ar
Cxcl12	Cygb	Arap2
Cyth3	Daam2	Arfip2
Cyyr1	Dcun1d3	Arhgap28
D8Ertd82e	Ddah1	Arhgef3
Dab2ip	Dgkh	Arl2bp
Dach1	Disp1	Arl4d
Daglb	Disp2	Arl6
Dcbld1	Dlg4	Armcx2
Dclk1	Dll1	Armcx6
Ddah2	Dock2	Arsa
Dennd3	Dock8	Arsb
Des	Dpysl3	Art3
Dhh	Dram1	Asap1
Dll4	Dusp3	Asns
Dok4	E2f7	Atic
Dsg2	Edn1	Atl3
Dusp1	Ednra	Atm
Dusp8	Eepd1	Atp13a3
Dysf	Efnb1	Atp1b1
Ednrb	Egflam	Atp6v0a1
Eef1b2	Egln3	Atpaf1
Efhd1	Ehd4	Azin1
Efnb2	Eln	B3gnt2
Efr3b	Enah	B3gnt3
Ehd3	Enpep	B4galt5
Emp1	Enpp2	BC003331
Endou	Entpd1	BC018465
Esm1	Eogt	BC021614
Etl4	Epha4	BC028528
F8	Epsti1	BC057079
Fabp4	Etv6	Bcap29
Fabp5	Extl1	Bcar3
Fam101b	F2r	Bcl2
Fam134b	F830016B08Rik	Bclp2
Fam13c	Fads3	Bcs1l
Fat4	Fam117b	Bdkrb2
Fhod1	Fam124a	Bet1
Filip1	Fam174b	Bhlha15
Fkbp5	Fam198b	Bmp1
Flt1	Fam57b	Boc
Flt4	Fam65b	Brp44l

Fmnl3	Fas	Btbd11
Fn1	Fblim1	Btnl9
Fnbp1l	Fbln5	BY080835
Foxp4	Fgl2	C1ql3
Fscn1	Fjx1	C1rb
Gaa	Fndc1	C1rl
Gas7	Foxc1	C1s
Gcnt1	Foxf1	C230081A13Rik
Gem	Frmd6	C330006D17Rik
Gfpt1	Fryl	C4b
Gja4	Furin	C630004H02Rik
Gja5	Fut2	C77370
Glipr2	Fxyd6	Cad
Glul	Fyb	Cald1
Glycam1	Gabarapl1	Cand2
Gm2a	Galnt16	Car4
Golm1	Galnt18	Caskin2
Gpc4	Gas1	Casp3
Gpihbp1	Gata2	Ccbp2
Gpr182	Gata6	Ccdc33
Gprc5b	Gbp6	Ccdc86
Hamp	Gfod1	Ccl21a
Hdac5	Gm13889	Ccnd2
Hecw2	Gm15055	Ccng1
Hey1	Gm4951	Cd79b
Hic1	Gm4980	Cdca7l
Hip1r	Gm694	Cdh2
Hspa12b	Gna11	Cdk18
Hspa1a	Gpr1	Cdkn2aipnl
Hspa2	Gpr126	Ces2b
Id1	Gpr56	Ces2d-ps
Id3	Gramd4	Cgnl1
Ier2	Gucy1a3	Ch25h
Ier5l	Gucy1b3	Chrnb1
Iffo2	H2-Aa	Chrnb4
Igf2	H2-Ab1	Chst1
Igfbp3	H2-Eb1	Chst2
Il1r1	Hbb-bt	Chst4
Il27ra	Hdc	Cks1b
Il2rg	Hes1	Clca2
Il6st	Hexa	Cldn11
Iqsec2	Heyl	Clint1
Irak2	Hid1	Clptm1l
Isyna1	Hif1a	Clu
Itga1	Hist1h4a	Clybl
Itga2	Hs3st1	Cmbl

Itpkb	Hs3st3b1	Cnn2
Jag2	Hspb8	Cnpy2
Jup	Hsph1	Col4a1
Kank1	Igfbp4	Colec12
Kcnj15	Igfbp5	Copa
Kctd12b	Igfbp7	Copb2
Kdr	Ikzf1	Copg
Klf3	Il13ra1	Cops4
Klhl29	Impdh1	Copz1
Klhl36	Inf2	Coro1a
Lama3	Insr	Coro1c
Lama4	Iqgap1	Creg1
Lamb1	Irf2bpl	Crem
Laptm4b	Irf8	Crip2
Lbp	Irs2	Crispld1
Lcp1	Itga3	Csf1
Lgals3bp	Itga6	Csnk1e
Limch1	Itga7	Csrnp2
Lims2	Itga9	Ctage5
Lipg	Itgal	Ctla2b
Lrg1	Itgb4	Cttnbp2
Lrrc16a	Itih5	Cuedc1
Ltbp2	Itpr1	Cul4b
Ly6a	Jam3	Cul7
Ly6c1	Jdp2	Cxcl10
Lyve1	Kank3	Cxcl11
Man1a	Kank4	Cxcr4
Man2b1	Kazn	Cxcr7
Map4k2	Kcna5	Cxx1c
Map4k3	Kcnb1	Cyb5b
Marveld1	Kcne4	Cyp27a1
Mast4	Kcnj8	Cyp2d22
Mcc	Khnyn	Cyp51
Mcf2l	Kifc3	D030025P21Rik
Me1	Klf10	D19Wsu162e
Mepce	Klf11	D1Ertd622e
Mertk	Klhl3	D630042P16Rik
Met	Klhl6	Dab1
Mgll	Ksr1	Dab2
Mgp	Lama2	Dap
Micall2	Lck	Dcaf12l1
Mlec	Lef1	Dctd
Mpzl1	Lepr	Ddit4
Mxd4	Leprel2	Ddost
Myadm	Lgmn	Ddr2
Myo10	Lhfp	Ddx1

Myo1e	Lifr	Ddx26b
N4bp2	Lipe	Ddx54
Naaa	Lmo7	Ddx60
Naglu	Lnx2	Decr1
Nav1	Lox	Dennd2c
Nav2	Loxl4	Dennd2d
Nckap5l	Lpl	Dennd4a
Nes	Lrmp	Depdc6
Nfatc4	Lrrc3b	Derl2
Nid2	Lurap1l	Dgkd
Nmt2	Lzts2	Dhcr24
Nos2	Map3k5	Dhdh
Notch3	Map3k6	Dhrs11
Notch4	Map3k8	Dhx33
Nova2	Map6	Dkc1
Nr2f2	Marcksl1	Dkk2
Nr4a1	Mb21d1	Dnahc6
Nrp1	Mbp	Dnajc10
Odc1	Megf6	Dnajc13
Osbpl5	Mical2	Dnm3
P2ry2	Mllt4	Doc2b
Palm	Mme	Dock10
Pam	Mmrn1	Dock5
Parp8	Mnd1	Dpp7
Pcdh1	Mpeg1	Dram2
Pcdh7	Mr1	Dtymk
Pde7b	Mras	Dusp5
Pdgfb	Mt1	Ece2
Pdgfrb	Mtss1l	Echdc1
Pdia5	Myh10	Eda2r
Per1	Myo1b	Efhd2
Pgm5	Myof	Efna1
Phldb1	Myrip	Egfl8
Phlpp1	N4bp3	Egr2
Pik3c2b	Nbl1	Egr3
Pik3cb	Nck2	Eif1
Pik3r3	Neat1	Eif2s3x
Pik3r6	Net1	Eif3g
Pitpnc1	Nod2	Eif5a
Pkig	Notch2	Elovl6
Plcb1	Nov	Elp2
Plekha3	Npc2	Eml1
Plekha7	Ntn4	Endod1
Plekhg5	Nuak1	Enpp3
Plin2	Nxpe4	Enpp5
Plxna2	Palld	Entpd2

Plxnb2	Pcdh12	Entpd4
Podxl	Pcdh19	Epas1
Ppap2a	Pcp4l1	Epb4.1l4a
Ppap2b	Pdlim1	Ephx1
Pparg	Peak1	Ephx2
Prcp	Per2	Eprs
Prdm16	Pi16	Eps8l2
Prickle2	Piezo2	Ercc1
Prkag2	Pkm	Erlec1
Prrg3	Plscr1	Ern1
Ptgs2	Pltp	Esrrg
Ptp4a3	Plxna4	Ets1
Ptprg	Ppp1r10	Ets2
Rab3il1	Ppp1r13l	Etv1
Ralgds	Ppp1r14a	Evl
Rapgef1	Ppt1	Exoc3l
Rapgef3	Prex1	Exoc3l2
Rapgef4	Prkcg	Extl2
Rarg	Procr	F2rl3
Rasal1	Prom1	Fads1
Rasd1	Prr7	Fads2
Rasgrf2	Prrx1	Fah
Rasgrp3	Psen2	Fam102a
Rbp7	Psme2	Fam107b
Rcsd1	Ptgfrn	Fam114a1
Rdh10	Ptgs1	Fam120a
Rfk	Ptk2b	Fam20a
Rftn1	Ptk7	Fam33a
Rgs5	Ptn	Fam38b
Rhbdf2	Ptpn14	Fam46c
Rnf144a	Ptprc	Fam55b
Rnf19b	Pxdn	Fam69a
Rps6ka2	Rab11fip5	Fam84b
Rrad	Rab8b	Fam98a
Rsad2	Rai14	Fasn
Rtp3	Ralgapa2	Fbn1
Sdc3	Rapgef2	Fbxo36
Sec14l1	Rasal3	Fdft1
Sele	Rasgef1b	Fermt2
Selp	Rasgrp2	Fgd6
Sema3g	Rassf1	Fgfr1
Sema4c	Rbp1	Fgfr2
Sema7a	Rcan1	Fhl1
Septin4	Ret	Fkbp10
Serpina1e	Rgcc	Fkbp1b
Serpina3f	Rgma	Flrt3

Serpinb1a	Rgs1	Fnbp1
Serpinb9	Rgs12	Fndc3a
Serpinh1	Rgs16	Frmd4a
Sertad1	Rgs2	Frmd4b
Sesn3	Rin3	Fuca1
Setbp1	Rnd1	Fuca2
Sez6l2	Rps3	Fundc2
Sgk1	Rusc2	Fut7
Sh2b2	S100a6	Fut8
Sh2b3	S1pr3	Fyn
Sh2d3c	Satb1	Fzd6
Sh3rf1	Scn1b	G6pc3
Shroom3	Serpina3g	Galns
Sipa1	Serpinb6b	Galnt12
Sipa1l2	Serpine1	Galntl2
Sirpa	Sh3bgrl	Galntl4
Slc16a13	Sh3tc2	Gart
Slc16a9	Shb	Gas6
Slc1a5	Slc12a2	Gatsl3
Slc20a1	Slc16a2	Gbas
Slc26a2	Slc28a2	Gbe1
Slc2a1	Slc30a1	Gcc2
Slc6a6	Slc37a2	Gchfr
Slc9a3r2	Slc39a14	Gcom1
Slco2b1	Slc7a2	Gga2
Smtn	Slco2a1	Ggt5
Snai1	Slco3a1	Ggta1
Snx5	Smap2	Gipc3
Socs2	Sncg	Glb1
Sorbs1	Sod2	Glb1l
Sorbs3	Sod3	Glo1
Sox13	Soga1	Glt25d2
Sox17	Spats2	Gm10851
Sox18	Spns2	Gm13051
Sparc	Spred2	Gm13251
Spata13	Spsb1	Gm14137
Spint2	Ssu2	Gm16486
Spry1	St3gal4	Gm5077
Spry4	St5	Gm5972
Stard8	St6gal1	Gm8995
Steap3	St6galnac2	Gm9766
Susd2	Stab1	Gm9943
Syne1	Steap4	Gmds
Syne2	Stk10	Gmppa
Tagln	Stk32c	Gnat1
Tbl1xr1	Sulf1	Gne

Tbx3	Sulf2	Gnl3l
Tcf15	Sybu	Gnptab
Tfpi2	Syngr2	Golga7
Tgfbi	Syt15	Golgb1
Thbd	Tanc1	Golph3
Thsd7a	Tank	Golph3l
Timp2	Tbc1d30	Golt1b
Tinagl1	Tes	Gorasp2
Tmcc2	Tet3	Gpa33
Tmem173	Thbs1	Gpr124
Tmem2	Thrsp	Gpr35
Tmem44	Timeless	Gpr4
Tnfrsf11a	Tiparp	Gpr68
Tnfrsf9	Tll1	Gpr81
Tox	Tlr2	Gpr97
Tpst1	Tmem158	Gprc5a
Traf1	Tmem98	Gpx8
Trp53i11	Tmod2	Grb10
Tsc22d1	Tmsb10	Grin2d
Tsc22d3	Tnfrsf10b	Gspt1
Tuba1a	Tnfrsf21	Gstm1
Txnip	Tnks1bp1	Gstz1
Uaca	Tpm1	Gypc
Ubd	Trak1	H13
Ugcg	Trf	H28
Unc119b	Tril	H2-T24
Unc5b	Trp53inp2	H47
Vav2	Tsku	Hap1
Vldlr	Tspan13	Hba-a1
Xbp1	Tspan2	Hba-a2
Xdh	Tspan5	Hdac3
Zfp366	Tssc4	Hdac9
Zfp521	Upp1	Hddc2
Zfpm2	Vamp5	Hdlbp
	Vash1	Hexim1
	Vav1	Hist1h4f
	Vcam1	Hlx
	Vps13d	Hmbox1
	Vstm4	Hmbs
	Vtn	Hmcn1
	Wbp1l	Hmgcs2
	Wdfy4	Hn1l
	Wipf3	Hnrnph1
	Xylt2	Hoxd8
	Ypel2	Hpvc-ps
	Zbtb16	Hsd17b10

	Zbtb46	Hsd17b7
	Zdbf2	Hsd3b7
	Zfp423	Hspa13
	Zfp57	Hspb1
	Zfp703	Hspg2
	Zfp827	Hunk
		Hvcn1
		Hyal2
		Hyou1
		Ibtk
		Ica1
		Id4
		Ide
		Idh3a
		Idi1
		Ifi204
		Ifit1
		Ifit2
		Ifit3
		Ifngr2
		Ift52
		Il10rb
		Il17rd
		Il4i1
		Il6
		Imp3
		Inhbb
		Inpp4b
		Inpp5k
		Ints2
		Ints9
		Ipo4
		Irf7
		Iscu
		Itgb5
		Itih3
		Jak1
		Junb
		Kars
		Kat2a
		Kcne3
		Kcng3
		Kcnh1
		Kcnk5
		Kcnq1
		Kdelr3

		Kdm4d
		Kdm6b
		Klf2
		Klf4
		Klf9
		Klhl13
		Kremen1
		L3mbtl2
		Lactb2
		Lamc1
		Lats2
		Ldb2
		Ldhb
		Ldlr
		Ldlrad3
		Lhfpl2
		Lhpp
		Lhx2
		Lipa
		Litaf
		Llph
		Lmcd1
		Lmna
		LOC100504608
		Lpar6
		Lpcat4
		Lpin1
		Lrp3
		Lrp5
		Lrpprc
		Lrrc1
		Lrrc32
		Lrrc33
		Lrrc59
		Lrrc8b
		Lrrc8c
		Lrrk2
		Lss
		Ltbp4
		Ly6c2
		Ly96
		Lyn
		Lztfl1
		Madcam1
		Maged1
		Magix

		Magt1
		Map1lc3a
		Map4k4
		Mapk6
		Marcks
		Mast2
		Mat2a
		Mbnl3
		Mdn1
		Mecr
		Mef2c
		Megf9
		Meox2
		Mest
		Metap1
		Mettl1
		Mex3b
		Mex3d
		Mfge8
		Mgat2
		Mgst3
		Mina
		Mir15a
		Mir22
		Mir23a
		Mir27a
		Mirlet7e
		Mki67ip
		Mmachc
		Mmd
		Mmp15
		Morc4
		Morn2
		Mrpl38
		Mrps18b
		Msx1
		Mt2
		Mthfd2l
		Mtmr10
		Mustn1
		Mycn
		Myh11
		Myl9
		Mylip
		Myo19
		Myo1d

		Naa25
		Nans
		Nat10
		Nceh1
		Ncf2
		Ncrna00085
		Nebl
		Nedd9
		Nefh
		Neo1
		Nfil3
		Nfkbie
		Nicn1
		Nipsnap1
		Nme1
		Nme2
		Nnt
		Nol6
		Nos3
		Notch1
		Npnt
		Npr2
		Npr3
		Nr4a2
		Nr4a3
		Nrarp
		Nsdhl
		Nt5dc2
		Nt5e
		Nucb2
		Nudcd1
		Nudt19
		Nxph1
		Oas1a
		Oas1g
		Oas2
		Obfc2a
		Ogfrl1
		Oit1
		Olfml2a
		Orai2
		Otud1
		Pa2g4
		Pabpc4
		Palmd
		Papss2

		Parm1
		Pcolce
		Pcsk5
		Pctp
		Pde10a
		Pde1b
		Pde9a
		Pdgfa
		Pdgfd
		Pdia6
		Pdk4
		Peg10
		Pemt
		Pex10
		Pgcp
		Pglyrp1
		Pgm3
		Phkb
		Pign
		Pir
		Pkd2l2
		Pkdcc
		Plat
		Plau
		Plcb4
		Plcd1
		Plcg2
		Plekha1
		Plekhb2
		Plekho1
		Plk2
		Plscr4
		Plxdc2
		Plxnb1
		Plxnd1
		Pmepa1
		Pmp22
		Pno1
		Pnpla7
		Pnpla8
		Pnpo
		Poglut1
		Pole
		Polg2
		Polr1a
		Pomt1

		Pou2af1
		Ppap2c
		Ppic
		Ppm1j
		Ppm1k
		Ppp1r14b
		Ppp1r2
		Pqlc3
		Prdm1
		Prepl
		Prex2
		Prkcd
		Prmt5
		Prnd
		Prps2
		Prr5l
		Prss23
		Psme3
		Pter
		Ptgds
		Ptger4
		Ptpn3
		Ptprb
		Ptprk
		Ptprm
		Pvrl1
		Pvrl2
		Pwp1
		Pxmp2
		Qpctl
		Rab27a
		Rab32
		Rabl3
		Ramp3
		Rap1gap
		Rars
		Rasd2
		Rasgef1a
		Rasgrp1
		Rassf6
		Rbm47
		Rbms1
		Rbpms2
		Rcc2
		Rdh1
		Rdh9

		Relt
		Renbp
		Rex2
		Rfwd3
		Rgl1
		Rgnef
		Rgs19
		Rhbdf1
		Rhob
		Rhoc
		Rhod
		Rhpn2
		Rin2
		Ripk3
		Rnf122
		Rnf141
		Rnf181
		Robo1
		Robo2
		Rpn1
		Rprl1
		Rsph3a
		Rtn4ip1
		S100a11
		Samsn1
		Sat1
		Sc4mol
		Sc5d
		Scarb1
		Scarna17
		Sccpdh
		Scd1
		Scfd1
		Scgb3a1
		Scube1
		Scube3
		Sdf2l1
		Sdpr
		Sec11c
		Sec22b
		Sec23b
		Sec24d
		Sec31a
		Sec61a1
		Selm
		Sema5a

		Sema6a
		Sema6d
		Serf1
		Serp1
		Serpina1a
		Serpina1b
		Serpina1c
		Serpina3n
		Septin6
		Serpinb8
		Serpinf1
		Serping1
		Sfxn1
		Sgce
		Sh3gl3
		Shmt1
		Sil1
		Sirt1
		Slamf8
		Slc10a6
		Slc12a8
		Slc16a1
		Slc16a11
		Slc22a23
		Slc24a6
		Slc25a1
		Slc25a16
		Slc25a23
		Slc25a25
		Slc26a10
		Slc29a3
		Slc2a12
		Slc2a13
		Slc33a1
		Slc35a3
		Slc37a1
		Slc38a1
		Slc38a2
		Slc38a9
		Slc39a11
		Slc41a3
		Slc43a2
		Slc44a2
		Slc45a4
		Slc7a7
		Slit3

		Smad6
		Smad7
		Smpdl3a
		Smyd5
		Snai2
		Snap23
		Sncaip
		Snd1
		Snora31
		Snord14c
		Snord14e
		Snord57
		Sntb1
		Snx20
		Snx33
		Sort1
		Sox7
		Sp100
		Sparcl1
		Spcs3
		Sphk1
		Spna2
		Sqle
		Srgap3
		Srm
		Srp54b
		Srp54c
		Srp68
		Srp72
		Srr
		Sspo
		Ssr1
		Ssr3
		Ssr4
		Sstr4
		St3gal1
		St8sia4
		St8sia6
		Stab2
		Stap2
		Stard4
		Stard9
		Stau2
		Stc1
		Stom
		Ston1

		Stra6
		Stt3a
		Stxbp2
		Styx
		Sult1a1
		Sun2
		Surf4
		Sykb
		Syn2
		Synpo
		Syt1
		Taf1d
		Taf4b
		Tarbp1
		Tatdn1
		Tbc1d1
		Tbc1d4
		Tbc1d8
		Tbc1d8b
		Tbxa2r
		Tc2n
		Tcerg1l
		Tcf20
		Tcf3
		Tcf7l1
		Tcfcp2l1
		Tek
		Tenc1
		Tfb2m
		Tfg
		Tfpi
		Tgfb1i1
		Tgfb2
		Tgfbr1
		Tgfbr2
		Timm23
		Timp1
		Timp3
		Timp4
		Tle3
		Tlr1
		Tlr4
		Tm6sf1
		Tmc8
		Tmed3
		Tmem150c

		Tmem154
		Tmem167
		Tmem180
		Tmem184b
		Tmem184c
		Tmem19
		Tmem20
		Tmem204
		Tmem214
		Tmem8
		Tmem97
		Tmx3
		Tnc
		Tnf
		Tnfrsf11b
		Tnfsf15
		Tnip1
		Tnk2
		Tns1
		Tob1
		Tpp1
		Tppp3
		Tra2a
		Trabd
		Tram1
		Trpv4
		Tshr
		Tspan12
		Tspan14
		Tspan3
		Tspan6
		Tspan8
		Tsr1
		Ttc9c
		Ttl
		Ttll1
		Ttll4
		Tubb2a
		Txndc11
		Uba5
		Ubl7
		Ufsp2
		Uggt1
		Uhrf1bp1l
		Unc93b1
		Uprt

		Uso1
		Usp10
		Usp11
		Usp14
		Usp3
		Usp9x
		Utp15
		Vamp7
		Vegfb
		Vegfc
		Vgll4
		Vps13c
		Vrk2
		Vwf
		Wbscr17
		Wbscr27
		Wdr36
		Wdr41
		Wdr77
		Wrb
		Wsb1
		Wscd1
		Wwtr1
		X99384
		Xrcc5
		Yipf5
		Yipf6
		Zbp1
		Zbtb4
		Zc4h2
		Zdhhc18
		Zdhhc2
		Zdhhc9
		Zfhx2
		Zfp106
		Zfp503
		Zfp715
		Zfp9
		Zyx

*, The signature gene list for HEC is from the published study: Lee M, et al. Transcriptional programs of lymphoid tissue capillary and high endothelium reveal control mechanisms for lymphocyte homing. Nature immunology 15, 982-995 (2014).

Supplementary Table 4: Antibody information.

Antibody	Clone number	Catalog number
anti-B220	RA3-6B2	eBioscience, 12-0452
anti-CD4	GK1.5	eBioscience, 25-0041
anti-CD8	53-6.7	eBioscience, 47-0081
anti-CD11b	M1/7	eBioscience, 12-0112
anti-CD11c	N418	Biolegend, 117307
anti-CD25	PC61	Biolegend, 102029
anti-CD31	MEC13.3	Biolegend, 102507
anti-CD44	IM7	Biolegend, 103006
anti-CD45	30-F11	eBioscience, 45-0451
anti-CD45.1	A20	Biolegend, 110705
anti-CD45.2	104	eBioscience, 45-0454
anti-c-Kit	2B8	eBioscience, 17-1171
anti-Collagen IV	Polyclonal	Cosmo Bio Co., LTD., LSL-LB-1407
anti-EpCAM	G8.8	eBioscience, 17-5791
anti-Gr-1	RB6-8C5	Biolegend, 108408
anti-Ly6C	HK1.4	Biolegend, 128017
anti-NK1.1	PK136	eBioscience, 12-5941-81
anti-P-selectin	RB40.34	BD, 561923
anti-Ter-119	Ter-119	eBioscience, 12-5921

Supplementary Table 5: Primers used for quantitative real-time PCR.

Gene	Forward primer	Reverse primer
b-Actin	ACACCCGCCACCAGTTCGC	ATGGGGTACTTCAGGGTCAGGGTCAGGATA
Bst 1	CCTATCCCACGAGAGGGTTT	CACAGGATTCCACATTGGG
Ccl25	CCGGCATGCTAGGAATTATCA	GGCACTCCTCACGCTTGTACT
Cd55	CTAACACAGGTGGTGACCGTT	TCTTCGTAACTCTTCGTTGGC
Glycam-1	GAGGTGCAACCACCTCAGA	GATACGACTGGCACCAGAGA
Gapdh	TTCACCACCATGGAGAAGGC	GGCATGGACTGTGGTCATGA
Icam-1	GCTGTTTGAGCTGAGCGAGATCGG	TGAGGTCCTTGCCTACTTGCTGCC
Lyve-1	CTTGCAGCTATGGATGGGTT	TCGGATGAGTTGTGGCAATA
Pcdh7	GTGGGAGCAGGAGACAACAT	CGAAATGGCTGTTTGCTGTA
P-selectin	TCCAGGAAGCTCTGACGTACTTG	GCAGCGTTAGTGAAGACTCCGTAT
Ubd	CACCTGTGTTGTCCGTTCAG	GAGACCTTGGTTTGGGACCT
Vcam-1	TGGTGAAATGGAATCTGAACC	CCCAGATGGTGGTTTCCTT
